# Development, Implementation, and Evaluation Methods for Dashboards in Health Care: Scoping Review

**DOI:** 10.2196/59828

**Published:** 2024-12-10

**Authors:** Danielle Helminski, Jeremy B Sussman, Paul N Pfeiffer, Alex N Kokaly, Allison Ranusch, Anjana Deep Renji, Laura J Damschroder, Zach Landis-Lewis, Jacob E Kurlander

**Affiliations:** 1 Department of Internal Medicine, University of Michigan, 2800 Plymouth Road, NCRC Building 14, Ann Arbor, MI, 48109, United States, 1 734 430 5359; 2 Institute for Healthcare Policy and Innovation, University of Michigan, Ann Arbor, MI, United States; 3 Veterans Affairs Ann Arbor Center for Clinical Management Research, Ann Arbor, MI, United States; 4 Department of Psychiatry, University of Michigan, Ann Arbor, MI, United States; 5 Department of Medicine, University of California Los Angeles Health, Los Angeles, CA, United States; 6 Department of Learning Health Sciences, University of Michigan, Ann Arbor, MI, United States

**Keywords:** dashboard, medical informatics, quality improvement, electronic health record, scoping review, monitoring, health care system, patient care, clinical research, emergency department, inpatient, clinical management

## Abstract

**Background:**

Dashboards have become ubiquitous in health care settings, but to achieve their goals, they must be developed, implemented, and evaluated using methods that help ensure they meet the needs of end users and are suited to the barriers and facilitators of the local context.

**Objective:**

This scoping review aimed to explore published literature on health care dashboards to characterize the methods used to identify factors affecting uptake, strategies used to increase dashboard uptake, and evaluation methods, as well as dashboard characteristics and context.

**Methods:**

MEDLINE, Embase, Web of Science, and the Cochrane Library were searched from inception through July 2020. Studies were included if they described the development or evaluation of a health care dashboard with publication from 2018‐2020. Clinical setting, purpose (categorized as clinical, administrative, or both), end user, design characteristics, methods used to identify factors affecting uptake, strategies to increase uptake, and evaluation methods were extracted.

**Results:**

From 116 publications, we extracted data for 118 dashboards. Inpatient (45/118, 38.1%) and outpatient (42/118, 35.6%) settings were most common. Most dashboards had ≥2 stated purposes (84/118, 71.2%); of these, 54 of 118 (45.8%) were administrative, 43 of 118 (36.4%) were clinical, and 20 of 118 (16.9%) had both purposes. Most dashboards included frontline clinical staff as end users (97/118, 82.2%). To identify factors affecting dashboard uptake, half involved end users in the design process (59/118, 50%); fewer described formative usability testing (26/118, 22%) or use of any theory or framework to guide development, implementation, or evaluation (24/118, 20.3%). The most common strategies used to increase uptake included education (60/118, 50.8%); audit and feedback (59/118, 50%); and advisory boards (54/118, 45.8%). Evaluations of dashboards (84/118, 71.2%) were mostly quantitative (60/118, 50.8%), with fewer using only qualitative methods (6/118, 5.1%) or a combination of quantitative and qualitative methods (18/118, 15.2%).

**Conclusions:**

Most dashboards forego steps during development to ensure they suit the needs of end users and the clinical context; qualitative evaluation—which can provide insight into ways to improve dashboard effectiveness—is uncommon. Education and audit and feedback are frequently used to increase uptake. These findings illustrate the need for promulgation of best practices in dashboard development and will be useful to dashboard planners.

## Introduction

Health care systems must process and make sense of more incoming data than ever before. Understanding and acting on these data are essential to almost every aspect of the health care enterprise, from direct patient care and clinical research, in which real-time data are critical to safe, appropriate, and timely care, to the C-suite, where health systems are held financially accountable for the outcomes of their patients [1-4]. This process can be resource intensive. One large academic medical center reported expending roughly 180,000 person-hours and US $5 million dollars to prepare and report 162 quality metrics on inpatient and emergency department performance in a single year [5]. Increasingly, business intelligence tools are used to reduce this burden by streamlining data aggregation and reporting to facilitate continuous monitoring and improvement of key metrics [6-8].

Health care “dashboards,” which analyze and present dynamic data about individuals and systems in readily interpretable ways to provide high-level and current snapshots of important metrics, have become one of the most common tools in this armamentarium. In modern health systems, they are widely seen as indispensable and are commonly used for clinical management, population health management, and quality improvement [6,9,10]. Despite dashboards’ ubiquity in health care, there is little research on them and how they have been used in practice [11,12]. Fundamental questions such as how they are developed, implemented, and evaluated have largely gone unexplored [6,13-15]. Yet consideration of each of these stages is critical to the successful implementation of any innovation, including dashboards. Indeed, health care systems are complex entities, containing diverse stakeholders with multiple overlapping and sometimes conflicting information needs [16,17]. Consequently, the development and dissemination of a dashboard is not a straightforward or linear process, but rather has been described as an “unpredictable, messy, and iterative process” involving multiple stakeholders [18].

In this scoping review, we apply the lens of implementation science—which addresses how to improve uptake of an innovation by accounting for contextual factors of the setting—to dashboards in health care settings, asking the questions of how developers have approached the interconnected steps of development, implementation, and evaluation. Specifically, we investigate the methods used to identify factors affecting uptake, strategies used to increase uptake, and evaluation methods. With this approach, we hope to draw attention to the need for systematic approaches to dashboard development and dissemination that incorporate principles of implementation science, identify common practices, and ultimately accelerate the science of dashboards.

## Methods

### Overview

In this scoping review, we followed Arksey and O’Malley’s [19] and Levac et al’s [20] frameworks for scoping review methodology to identify and map relevant literature. Methods and results are reported according to the PRISMA-ScR (Preferred Reporting Items for Systematic Reviews and Meta-Analyses extension for Scoping Reviews) checklist [21].

The conceptual framework for this review was the Generic Implementation Framework [22], which describes the core activities required for implementation of a practice. According to the Generic Implementation Framework, the process of implementation consists of three nonlinear and recursive stages: (1) identification of key factors, namely barriers and facilitators to uptake; (2) selection of strategies to increase uptake; and (3) evaluation. At the center of the process are the innovation itself and the context, which impact each of these 3 stages. In this review, we operationalized this framework as the following overarching questions:

1. What methods have been used to identify factors affecting dashboard uptake?
2. What strategies have been used to increase uptake?
3. What evaluation methods have been used?

Additionally, we investigated the topic and users of the dashboard and the context.

### Study Selection and Screening

The search strategy was developed with a research librarian and previously reported [23]. Briefly, in July 2020, we searched MEDLINE, Embase, Web of Science, and the Cochrane Library databases from inception through July 2020, using key terms, medical subject headings, and Boolean operators, with no date, language, or other restrictions applied. All records were uploaded into Covidence screening software for deduplication and dual reviewer screening of titles and abstracts. All studies describing use of a dashboard within a health care setting were included for full-text independent review by 2 study team members (authors: DH, ADR, AR, and ANK; other study team members: Rebecca Goldberg, Marisa L Conte,and Oliver Jintha Gadabu).

Studies were eligible if they described how a dashboard was developed, implemented, or evaluated in a health care setting; were published in English since 2018; and were used successfully in routine workflow [23]. Although we had initially planned to include any studies published in English since 2015, because of unplanned staffing and resource limitations, the inclusion criteria were updated to focus solely on the more recent years 2018‐2020. Exclusion criteria were implementation of the dashboard only in a pretesting environment and use solely for public health disease tracking or undergraduate medical education. Any disagreements on eligibility were resolved through discussion, with adjudication by a third author when needed. In the full-text screening stage, for any clinical trial registrations (eg, ClinicalTrials.gov NCT number), ClinicalTrials.gov was visited and reviewed for linked publications, which were imported into Covidence for deduplication and full-text screening.

### Data Extraction and Coding

A data extraction form was developed a priori [23]. In meetings, the extraction form was iteratively refined and a codebook of response options for categorical variables (eg, health care setting, dashboard purpose) was developed (Multimedia Appendix 1). Data were extracted using Qualtrics.

For all included dashboards, data were extracted on health care context, dashboard purpose, intended end user, and design features; methods used to identify factors affecting uptake; strategies used to increase uptake; and evaluation methods, using predefined attributes (see Table 1 for definitions of common purposes, and Multimedia Appendix 1 for full codebook definitions). For purposes of data extraction, a list of methods for identifying factors affecting uptake was informed by existing guidelines for curating health data and designing health informatics interventions for practice improvement (eg, use of theoretical frameworks, end user involvement, formative usability testing, benchmarking based on established guidelines) [24,25]. Strategies used to increase uptake were informed by the Expert Recommendations for Implementing Change strategy list [26], a widely used compendium of implementation strategies.

**Table 1.** Dashboard purpose definitions from study codebook^a^.

Dashboard purpose	Codebook definition
**Clinical purposes**	
Direct patient care	A dashboard used when providing direct, immediate care to a patient in any health care setting.
Population health management	A dashboard used to identify patients in a clinic panel, department, or unit who are at risk for an adverse event or in need of intervention (eg, dashboard identifies patients with potentially unsafe prescribing).
Care coordination	A dashboard that supports care coordination by pulling information from multiple data sources and allowing both the patient and a health care provider to view the dashboard, and/or allowing the patient to enter self-reported health data into the dashboard to complement electronic health record information for the clinician to use for care planning and decision-making.
**Administrative purposes**	
Performance monitoring	A dashboard that provides data on individual provider or unit/site performance. These dashboards often show performance trends over time as well as offer the user the ability to compare their performance to that of peers or to averages within their department.
Utilization tracking	A dashboard used to provide data on health care utilization, either at the level of the patient (eg, how often they visit, how long visits take, where the patient is seen) or at the level of the department or organization (eg, services per day/month/year, services by category or unit, top services provided by cost or in a given time period).
Resource management	A dashboard used to support resource management by providing data to support adequate staffing, ensure appropriate and adequate supplies are available, and monitor bed management and patient transfers.

a Definitions for the most commonly reported dashboard purposes are displayed here. All codebook definitions are reported in Multimedia Appendix 1.

### Analysis

Due to the large number of categories for dashboard purposes and end users, we created larger super-categories for these domains so data could be summarized at a higher level. For both domains, these super-categories were “administrative/nonclinical,” “clinical,” “both administrative/nonclinical and clinical,” or “research” (Tables S1 and S2 in Multimedia Appendix 2).

For dashboard purpose, the following attributes were considered: (1) clinical (direct patient care, population health management, and care coordination) and (2) administrative (performance monitoring; utilization tracking; resource management; financial tracking; alert or best practice advisory tracking; facilitating clinical or quality registry use; supporting education or training; and facility management). Some dashboards were categorized as both clinical and administrative (eg, used for performance monitoring and population health management). Dashboards used solely to support clinical research activities were categorized as research.

For end users, the following attributes were considered: (1) nonclinical (leadership, administrators, and individuals involved in quality improvement efforts) or (2) clinical (frontline clinicians, pharmacists, clinician trainees, remote monitoring staff, and clinical research teams). Some dashboards were categorized as having both nonclinical and clinical end users (eg, used by leadership or administration and frontline clinicians).

Extracted data for variables of interest are reported as counts and percentages. Additional data on the methods used to identify factors affecting uptake, implementation strategies used, and evaluation type are reported by purpose category (eg, clinical or administrative). Data on dashboard development and design characteristics are described narratively in online appendices and summarized in the text. Citations are provided in the text for results with ≤20 references, though all extracted data are available in Multimedia Appendix 3 for download online and can be filtered by variable of interest to identify any relevant studies.

## Results

### Study Screening Process

**Figure 1.**
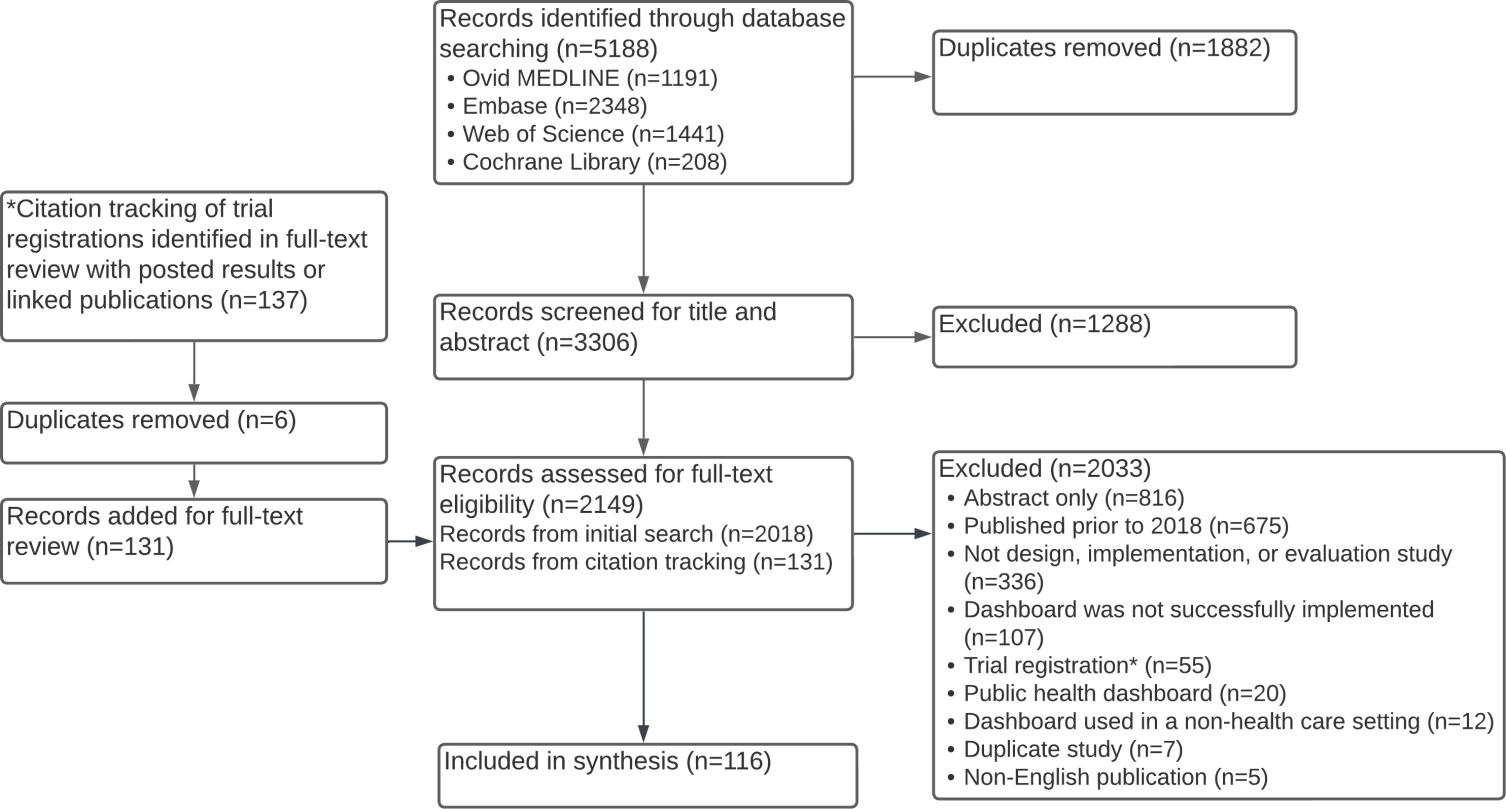
Preferred Reporting Items for Systematic Reviews and Meta-Analyses extension for Scoping Reviews (PRISMA-ScR) flow diagram.

A total of 3306 unique studies were identified and underwent title and abstract review; in all, 1288 articles were excluded, and the full texts of the remaining 2149 studies were screened (Figure 1). Ultimately, 116 studies that described 118 unique dashboards were included.

### Dashboard Characteristics and Context

Most dashboards were used in North America (79/118, 66.9%) or Europe (18/118, 15.2%, predominantly the United Kingdom; Table 2 and Table S3 in Multimedia Appendix 2). Seven US dashboards originated from the Veterans Affairs Health System [27-33]. The most prevalent settings were inpatient (n=45, 38.1%), outpatient clinics (n=42, 35.6%), and emergency services (n=18, 15.2%) [27,34-50]; in addition, 12/118 (10.1%) were used in >1 health care setting [34-36,38-40,42,46,47,51-53]. Frontline clinicians (97/118, 82.2%, predominantly physicians) and leadership or administrators (50/118, 42.4%) were frequent end users, often in combination (28/118, 23.7%; Table S2 in Multimedia Appendix 2). Patients were sometimes included as end users (12/118, 10.2%) [54-65].

**Table 2.** Setting, purpose, and end users of included dashboards.

Characteristic	Dashboards (n=118), n (%)
**Publication year**	
* *2018	38 (32.2)
2019^a^	39 (33)
2020^b, c^	41 (34.7)
**Health care setting** ^**d**^	
Inpatient setting	45 (38.1)
Outpatient clinic	42 (35.6)
Emergency services	18 (15.2)
Other setting or unclear	12 (10.2)
Imaging or radiology facility	10 (8.5)
Surgical departments	7 (5.9)
Clinical laboratory	5 (4.2)
**Settings reported**	
* *1 setting	106 (89.8)
* *>1 setting	12 (10.1)
**Geographic location** ^e^	
North America	79 (66.9)
Europe	18 (15.2)
Asia	11 (9.3)
Africa	6 (5.1)
Australia	4 (3.4)
South America	0 (0)
**Purpose[Table-fn T2_FN11]** ^d^ ^, ^ ^f^	
***Clinical purposes***	
Direct patient car*e*	47 (39.8)
Population health management	37 (31.4)
Care coordination	22 (18.6)
***Administrative purposes***	
Performance monitoring	51 (43.2)
* *Utilization tracking	30 (25.4)
Resource management	22 (18.6)
Financial tracking	4 (3.4)
Facility management	0 (0)
Other nonclinical purpose^g^	6 (5.1)
***Other purposes***	
Clinical trial support tool	1 (0.8)
Other/unclear	0 (0)
**Number of purposes**	
1 purpose	34 (28.8)
2 or more purposes	84 (71.2)
**Intended end user[Table-fn T2_FN11]** ^d^ ^, ^ ^f^	
***Clinical end users***	
*Frontline clinicians*	97 (82.2)
Medical doctor or advanced practice provider	76 (64.4)
Registered nurse or medical assistant	34 (28.8)
Other or not specified	38 (32.2)
*Pharmacists or pharmacy staff*	12 (10.2)
*Patients*	12 (10.2)
*Clinician trainees*	5 (4.2)
*Remote monitoring staff*	4 (3.4)
*Clinical research teams*	2 (1.7)
***Nonclinical end users***	
Leaders and/or administrators	50 (42.4)
Quality improvement stakeholders	3 (2.5)
***Other***	8 (6.8)
End user not reported	2 (1.7)
**Number of end users**	
1 end user	54 (45.8)
2 or more end users	64 (54.2)

a One included study from 2019 described 2 dashboards (Woo et al [66]).

b One included study from 2020 described 2 dashboards (Stevens et al [67]).

c Databases were searched in July 2020 and only studies published and indexed in the databases searched by this date were screened for inclusion.

d Characteristics are reported by prevalence of selection of each response across dashboards without missing data for the variable of interest. As characteristics are reported by prevalence of selection, totals may be greater than 100%.

e Geographic location by country is available in Table S3 in Multimedia Appendix 2.

f Mapping of all purpose and user responses from data extraction to nonclinical, clinical, or both nonclinical and clinical groups is available in Tables S1 and S2 in Multimedia Appendix 2, respectively.

g Nonclinical purposes of education or training, facilitate use of clinical or quality registries, and tracking of alerts or best practice advisories were grouped as other. Definitions for all dashboard purposes are available in Multimedia Appendix 1.

The purpose was categorized as solely clinical in 43/118 (36.4%), solely administrative in 54/118 (45.8%), and both clinical and administrative in 20/118 (16.9%) studies [37,45,47,50,51,60,67-80] (Table 3). The most prevalent purposes of dashboards were performance monitoring (n=51, 43.2%), direct patient care (n=47, 39.8%), population health management (n=37, 31.4%), and utilization tracking (n=30, 25.4%; Table 2; definitions in Table 1). However, the majority of dashboards (n=84, 71.2%) met criteria for 2 or more purposes (Table 2). In dashboards with purpose(s) categorized as solely administrative (n=54), most included clinical end users (44/54, 81.5%); few were used solely by nonclinical staff (8/54, 14.8%) [35,36,81-85]; clinical users were almost always included as end users, regardless of purpose and setting (Table 2, cross tab of purpose × user group in Table S4 in Multimedia Appendix 2).

**Table 3.** Methods used to identify factors affecting uptake and key design characteristics.

Development characteristic	Overall (n=118), n (%)	Dashboard purpose group^a^, n (%)		
		Nonclinical (n=54)	Clinical (n=43)	Both nonclinical and clinical (n=20)
**Methods used to identify factors affecting uptake**				
Use of theoretical framework^b^	24 (20.3)	14 (25.9)	8 (18.6)	1 (5)
End user involvement in design^b^	59 (50)	26 (48.1)	23 (43.5)	10 (50)
Formative usability testing^b^	26 (22)	9 (16.7)	15 (34.9)	2 (10)
Benchmarks or metrics informed by regulatory guidelines^b^	43 (36.4)	23 (42.6)	13 (30.2)	7 (35)
**Software used for dashboard development** ^c^				
Software not reported	72 (61)	26 (48.1)	30 (69.8)	15 (75)
Custom coding build	14 (11.9)	6 (11.1)	7 (16.3)	1 (5)
Tableau	10 (8.5)	9 (16.7)	0 (0)	1 (5)
Microsoft Excel	6 (5.1)	6 (11.1)	0 (0)	0 (0)
Qlikview	4 (3.4)	3 (5.6)	0 (0)	1 (5)
Other software reported^d^	21 (17.8)	9 (16.7)	9 (20.9)	3 (15)
**Dashboard delivery channel** ^c^				
Website	41 (34.7)	15 (27.8)	21 (48.8)	4 (20)
Embedded within the electronic health record	17 (14.4)	3 (5.6)	9 (20.9)	5 (25)
Site intranet or SharePoint	13 (11)	10 (18.5)	2 (4.6)	1 (5)
Shared by email	12 (10.2)	11 (20.4)	0 (0)	1 (5)
Printed and posted in setting	12 (10.2)	6 (11.1)	2 (4.6)	4 (20)
Software app on phone, tablet, or computer	7 (5.9)	4 (7.4)	2 (4.6)	1 (5)
Other	10 (8.5)	5 (9.2)	5 (11.6)	0 (0)
Delivery channel not reported	29 (24.6)	13 (24.1)	8 (18.6)	8 (40)
**Dashboard data update frequency reported** ^c^				
Real time	31 (26.3)	11 (20.4)	13 (30.2)	7 (35)
Near–real time (5‐60 min)	11 (9.3)	4 (7.4)	2 (4.6)	4 (20)
Daily	16 (13.6)	10 (18.5)	4 (9.3)	2 (10)
Weekly, monthly, or quarterly	12 (10.2)	11 (20.4)	0 (0)	1 (5)
Various update times	5 (4.2)	2 (3.7)	1 (2.3)	2 (10)
Other	5 (4.2)	5 (9.3)	0 (0)	0 (0)
Update frequency not reported or unclear	38 (32.2)	11 (20.4)	23 (53.5)	4 (20)

a One dashboard that was categorized as “other” rather than nonclinical, clinical, or both, is not represented in the table. This dashboard was web-based with data reported in near–real time and reported use of a theoretical framework, but it did not report any end user involvement in design; formative usability testing; benchmarks or metrics informed by regulatory guidelines; software used to develop the dashboard; or any visual elements used in the dashboard display.

b Dashboard-level details on use of theory or frameworks, involvement of end users in dashboard development, formative usability testing, dashboard metrics informed by professional guidelines or by payor-specific or licensing agency–specific quality metrics, and details on software used to develop dashboards are available in Tables S5-S10 in Multimedia Appendix 2.

c Characteristics are reported by prevalence of selection of each response across dashboards without missing data for the variable of interest. As characteristics are reported by prevalence of selection, totals may be greater than 100%.

d Software responses selected for 3 or more dashboards are shown here, with software reported to be used for 2 or fewer dashboards reported as “other” in this table. Dashboard-level details are available in Table S5 in Multimedia Appendix 2.

### Dashboard Design Characteristics

The software or coding languages were reported for 46/118 dashboards (39%), with custom coding (14/118, 11.9%) [27,34,62,64,66,75,86-92], Tableau (10/118, 8.5%) [39,40,45,46,93-98], Microsoft Excel (6/118, 5.1%) [29,53,84,85,99,100], and Qlikview (4/118, 3.4%) [35,51,81,101] most commonly used (Table 3, details available in Table S5 in Multimedia Appendix 2). Dashboards developed using custom coding often described use of specific programs or coding languages, including SQL, JavaScript, and CSS. Overall, dashboards were most often available to end users as websites (41/118, 34.7%) or as tools embedded directly into the electronic health record (EHR; 17/118, 14.4%) [37,42,43,51,68,72,73,88,102-110] (Table 3, combinations reported in Table S6 in Multimedia Appendix 2). However, clinical dashboards were more likely to be web-based (21/43, 48.8%) or embedded in the EHR (9/43, 20.9%) [43,88,102-105,107,109,110], while dashboards with administrative purposes were more likely to be shared by email (11/54, 20.4%) [29,40,44,84,85,94,98,108,111-113], available via intranet or SharePoint (10/54, 18.5%) [27,39,40,46,86,87,90,93,94,114], or posted directly within the setting (6/54, 11.1%) [53,95,98,100,115,116].

Of dashboards that reported on updating frequency (80/118, 67.8%), most were updated in real time (31/118, 26.3%) or near–real time (5‐60 minutes; 11/118, 9.3%; Table 3) [37,41,42,67,68,74,95,103,117-119]. Dashboards used solely for administrative purposes were more likely to take 24 hours or more to update (21/43, 48.8%), while the majority of clinical dashboards updated every 24 hours or less (19/43, 44.2%; Table 3) [34,48,54,65,88,89,102-105,107,109,110,119-124].

### Methods Used to Identify Factors Affecting Uptake

**Figure 2.**
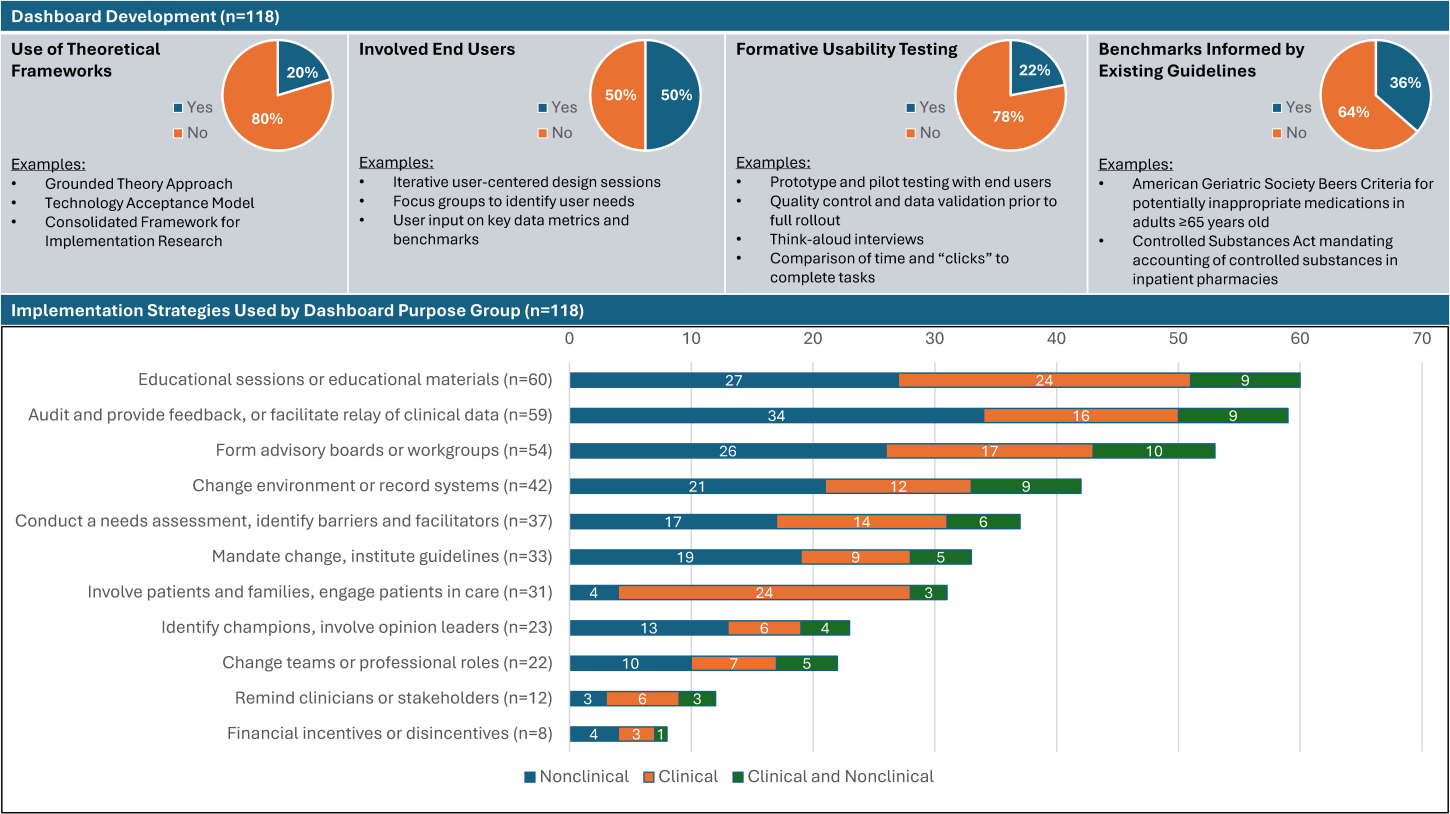
Strategies reported in dashboard development and implementation.

Half of included dashboards (59/118, 50%; Table 3, Figure 2) described steps to engage intended end users in the design process. User involvement included dashboard metric selection, data validation, and formation of work groups to iteratively review and revise dashboard prototypes, among other strategies (Table S7 in Multimedia Appendix 2). Fewer dashboards described formative usability testing (26/118, 22%; Table 3, Figure 2, Table S8 in Multimedia Appendix 2).

A theoretical or quality improvement framework was used to guide dashboard development, implementation, or evaluation efforts in 24 of 118 (20.3%) dashboards (Table 3, Figure 2). None of the frameworks were used in more than 2 studies. Reported theories and frameworks varied widely and included behavior change theories (eg, stages of change model [125], disruptive behavior pyramid theory [126], active choice principles [127]), technical frameworks (Unified Theory of Acceptance and Use of Technology [43], technology acceptance model [28]), implementation science–specific frameworks (eg, the Consolidated Framework for Implementation Research [76]; Expert Recommendations for Implementing Change [31]; Reach, Effectiveness, Adoption, Implementation, and Maintenance [105]), and a clinical governance framework [83], among others (dashboard-level details are available in Table S10 in Multimedia Appendix 2).

### Dashboard Data Content

Nearly one-third of health care dashboards used payor or accreditation organization reporting standards or professional guidelines as dashboard benchmarks or metrics of interest (43/118, 36.4%; Table 3, Figure 2). Dashboards designed for performance monitoring included metrics related to value-based payment and quality payment programs [72,128,129], or state- or national-level reporting mandates or guidelines [44,73,74,111]. When used for direct patient care or population health management, clinical guidelines were often used to identify patients for intervention or guide decision support (see [120,123,127] for examples; see Table S9 in Multimedia Appendix 2 for complete data). Of dashboards that reported on visual elements, tables (66/118, 55.9%), graphs (64/118, 54.2%), and color coding (61/118, 51.7%) were common display elements, as shown in Table 3.

### Dashboard Implementation

Most dashboards reported at least 1 implementation strategy (114/118, 96.6%; Table 4). Common implementation strategies and representative examples in citations included: (1) educational sessions or educational materials (60/118, 50.8%), which ranged from peer-led clinician education [38,107,109] to patient education on using the dashboard [59,122]; (2) audit and feedback or relay of clinical data (59/118, 50%), typically through one-on-one discussions between a clinician and a supervisor or academic detailer focused on how to improve performance or reach specific benchmarks [27,29,44,73,77,89,100,126]; and (3) formation of advisory boards or work groups, or engagement of stakeholders (54/118, 45.8%), which were often multidisciplinary groups of clinical staff, site leaders, and sometimes patients, who participated in dashboard development, implementation, or formative usability testing [35,38,46,58,68,91,95,97,102,105,129]. Other strategies included changing the physical environment or record systems (42/118, 35.6%; eg, placement of physical reminders or relevant supplies) as well as needs assessments or efforts to identify implementation barriers and facilitators (37/118, 31.4%). Although many implementation strategies were used at similar rates across dashboards with clinical and nonclinical purposes, audit and feedback was most often used alongside administrative dashboards (34/54, 63%; Table 4), especially those used for performance monitoring or utilization tracking. Conversely, when dashboards were used for clinical purposes, involving patients or families was more commonly reported (24/43, 55.8%), often to engage patients in shared decision-making (Table 4, Figure 2).

**Table 4.** Strategies used to increase dashboard uptake and evaluation methods.

Characteristics of implementation or evaluation	Overall (n=118)	Purpose group^a^		
Nonclinical (n=54)	Clinical (n=43)	Clinical and nonclinical (n=20)
**Strategies to increase uptake** ^b^				
Audit and provide feedback or facilitate relay of clinical data	59 (50)	34 (63)	16 (37.2)	9 (45)
Conduct educational sessions or disseminate educational materials	60 (50.8)	27 (50)	24 (55.8)	9 (45)
Conduct a needs assessment, identify barriers and facilitators	37 (31.4)	17 (31.5)	14 (32.6)	6 (30)
Form advisory boards or work groups	54 (45.8)	26 (48.1)	17 (39.5)	10 (50)
Identify champions, involve local opinion leaders	23 (19.5)	13 (24.1)	6 (14)	4 (20)
Mandate change, institute guidelines	33 (28.0)	19 (35.2)	9 (20.9)	5 (25)
Change teams or professional roles	22 (18.6)	10 (18.5)	7 (16.3)	5 (25)
Change environment or record systems	42 (35.6)	21 (38.9)	12 (27.9)	9 (45)
Involve patients and families, prepare patients to be active in care	31 (26.3)	4 (7.4)	24 (55.8)	3 (15)
Financial incentives or disincentives	8 (6.8)	4 (7.4)	3 (7)	1 (5)
Remind clinicians or other stakeholders	12 (10.2)	3 (5.6)	6 (14)	3 (15)
Other strategy reported	5 (4.2)	2 (3.7)	3 (7)	0 (0)
No adjunct implementation strategies reported	4 (3.4)	2 (3.7)	1 (2.3)	1 (5)
**Number of implementation strategies reported**				
* *0 implementation strategies	4 (3.4)	2 (3.7)	1 (2.3)	1 (5)
1‐3 implementation strategies^a^	67 (56.8)	28 (51.8)	26 (60.5)	12 (60)
4‐6 implementation strategies	37 (31.4)	20 (37)	13 (30.2)	4 (20)
7‐10 implementation strategies	10 (8.5)	4 (7.4)	3 (7)	3 (15)
**Evaluation type** ^c^				
**Quantitative evaluations only^a^**	60 (50.8)	29 (53.7)	20 (46.5)	10 (50)
Using dashboard/electronic health record data alone	41 (34.7)	25 (46.3)	11 (25.6)	5 (25)
Using survey alone	9 (7.6)	1 (1.9)	5 (11.6)	3 (15)
Using both dashboard/electronic health record and survey data	10 (8.5)	3 (5.6)	4 (9.3)	2 (10)
**Qualitative evaluations only**				
Using interview or focus group data	6 (5.1)	1 (1.9)	5 (11.6)	0 (0)
**Mixed method evaluations**				
Using both quantitative and qualitative data	18 (15.2)	7 (13)	8 (18.6)	3 (15)
**No evaluation reported**				
No evaluation reported	34 (28.8)	17 (31.5)	10 (23.3)	7 (35)

a One dashboard that was categorized as “other” rather than nonclinical, clinical, or both, is not represented in the table. This dashboard reported use of one implementation strategy (form advisory boards or workgroups) and included a quantitative evaluation with both electronic health record or dashboard data and survey data.

b Implementation strategies are reported by prevalence of selection of each strategy across included dashboards (n=118). Reported combinations of adjunct implementation strategies used will be reported separately.

c Evaluation type is reported as the combination of evaluation types selected.

### Dashboard Evaluation

Most dashboards included results from an evaluation of either the dashboard’s effect, using the dashboard as a tool for measuring change, or of the dashboard as both intervention and measurement tool (84/118, 71.2%; Table 4). Most evaluations were quantitative, using data from the dashboard or EHR alone (41/118, 34.7%), from the dashboard or EHR in combination with survey data (10/118, 8.5%) [35,41,47,56,57,70,86,88,92,96], or from surveys alone (9/118, 7.6%) [28,31,34,50,55,60,63,69,110]. An additional 18 studies reported mixed methods evaluations, which included interviews, focus groups, or analysis of chart notes [29,30,43,49,58,75-77,84,90,93,100,102,104,105,117,130,131]; only 6 reported results of qualitative assessments of end user perceptions of dashboards without a quantitative evaluation [52,59,119,132-134] (Table 4). When dashboards had an administrative purpose, evaluations more often were conducted using dashboard/EHR data (25/54, 46.3%).

## Discussion

### Principal Findings and Comparison With Prior Work

This scoping review of 118 dashboards used in health care settings provides an overview of the methods used to identify factors affecting uptake, strategies used to increase uptake, and evaluation methods. Creation of a dashboard does not ensure that it is used or that its aims are achieved. As with any new practice, effective development, implementation, and evaluation are interrelated steps that ultimately determine whether the practice achieves its goals, which requires careful attention to contextual factors and the content and aims of the dashboard itself. Our first principal finding is that most dashboards are foregoing steps during the development process to help ensure dashboards are suited to the needs of end users—for example, including such end users in the design process and conducting formative usability testing. Second, we have identified the most common implementation strategies used alongside dashboards, which are likely to be useful in planning future dashboard rollouts. Third, we found that 7/10 dashboards underwent an evaluation, predominantly quantitative evaluations, while only 2/10 included qualitative evaluation.

Despite the proliferation of dashboards, we found major opportunities to improve the development process of dashboards. Half of dashboards (59/118, 50%) did not involve end users in the development process and even fewer (26/118, 22%) included formative usability testing, both of which are effective strategies to improve usability and adoption [135,136]. It is recommended that dashboard developers involve stakeholders in an iterative development process and identify performance metrics that are meaningful, reliable, and timely [6,18,137,138]. This corroborates findings of a prior systematic review of safety dashboards, which found a minority used formative usability testing [139], and another recent scoping review, which found that, even when completed, usability testing is often incomplete [135]. The complexity of dashboards, exemplified by the multiplicity of purposes, end users, and settings often incorporated into a single dashboard, heightens the importance of thoughtful and deliberate usability testing in dashboard development [6,18]. When developing dashboards for clinicians, who are often overworked and burned out, usability testing will be crucial to making dashboard use as efficient and palatable as possible [140,141]. Physicians are also likely to be more engaged if health IT tools are perceived to provide direct benefit in carrying out their work [136].

We found a wide range of implementation strategies that have been paired with dashboards, often in combination: education; audit and feedback or relay of information; engagement of working groups, stakeholders, or advisory boards; changing the environment or electronic record systems; and conducting local needs assessments. Knowledge of possible implementation strategies is essential since the mere existence of a dashboard does not ensure its adoption. Prior studies involving dashboard implementation in the US Veterans Affairs health care system found most facilities used an array of implementation strategies to achieve desired quality and safety outcomes [31,142]. To improve the care of patients with cirrhosis, pairing a clinical dashboard with patient outreach was a particularly successful combination [142]. In our review, many studies similarly leveraged multiple strategies simultaneously to support uptake of dashboards and evidence-based practices. These findings can serve as a starting point for those planning implementation of a new dashboard. Ultimately, the choice of specific implementation strategies should depend on a thorough understanding of the local barriers and facilitators, in keeping with implementation theory [143,144].

It is encouraging that a large proportion of dashboards carried out at least some quantitative evaluation. Doing so likely requires little extra effort by evaluators since the necessary data may often be contained in the dashboard itself. Fewer dashboards performed qualitative evaluations, including methods like focus groups and semistructured interviews, which may add substantial value by providing deeper insights into the results of quantitative findings (the why) and point the way toward future dashboard enhancements to increase impact and sustainability [145].

### Strengths and Limitations

Strengths of our study include the comprehensiveness of the data elements extracted, including health care context, dashboard content and design characteristics, methods to identify factors affecting uptake, strategies used to increase uptake, and evaluation components. In addition, we included all studies in which a dashboard was implemented in a health care setting, which allowed us to capture the full scope of health care dashboards. Most prior reviews of dashboards in health care focused narrowly on specific settings [12,146], end users [147], and purposes [148,149]. By contrast, our inclusion criteria imposed few restrictions, leading to generalizability to a wider array of settings.

There are also some limitations. First, we excluded non-English publications, which limits the generalizability to other international settings. Second, our search ended in 2020 and thus represents a sample of published literature and did not capture the most recent trends in dashboards. Since our goals were not purely quantitative synthesis, this did not prohibit us from achieving the goal of broadly surveying dashboard development, implementation, and evaluation. Third, for studies in which the dashboard was not the focus (eg, when a dashboard was only a single part of a larger multicomponent intervention), the studies may not have included a complete description of the dashboard or the development, implementation, or evaluation process; thus, these elements may have been underreported.

### Implications

These limitations notwithstanding, our findings have implications for implementation of dashboards and research on dashboards in health care. Given the complexity of many dashboards, often with multiple purposes, settings, and end users simultaneously, stakeholder involvement in dashboard design, metric selection and iterative usability testing will be critical to ensure smooth and efficient operability for all end users. Usability testing may be particularly important for clinical care dashboards, not only because they have the potential to impact patients, but also because clinicians are already overloaded with administrative and documentation tasks and are increasingly burned out [150-152]. Relatively simple usability testing by novices can pay dividends, with the potential to increase adoption and effectiveness [153]. In a similar vein, dashboard evaluations should holistically consider potential impacts, including not only the performance indicator or quality measure of interest, but also those important to end users, like impact on workflow and efficiency. Finally, dashboard designers should be aware of the wide range of implementation strategies that have been used alongside dashboards and leverage implementation science and existing theory where possible to promote dashboard adoption and sustainability.

Future research priorities should include a quantitative review of the impact of dashboards on performance indicators, which was not covered in this scoping review; qualitative evaluations of the impact of dashboards on job satisfaction; and comparative research on the effectiveness of different development process and implementation strategies used with dashboards. The development of best practice statements or reporting checklists for publications on dashboard design may be useful. These will help to improve our understanding of how and why implementation strategies impact the effectiveness of these efforts [154,155].

### Conclusions

In this scoping review of implementation practices associated with dashboards used in health care settings, we have found major opportunities to ensure that dashboards meet the needs of end users and the clinical context; identified the most common strategies used to increase uptake; and demonstrated that quantitative evaluation methods significantly outnumber qualitative methods as part of dashboard evaluations. These findings will help to ensure that planners of future dashboards take steps to maximize implementation success and clarify the agenda needed to move the science of dashboards in health care forward.

## Supplementary material

10.2196/59828 Multimedia Appendix 1 Study codebook and examples.

10.2196/59828 Multimedia Appendix 2 Supplementary tables and additional findings.

10.2196/59828 Multimedia Appendix 3 Study data file.

10.2196/59828 Checklist 1 Preferred Reporting Items for Systematic Reviews and Meta-Analyses extension for Scoping Reviews (PRISMA-ScR) checklist.

## Abbreviations

EHR: electronic health record
PRISMA-ScR: Preferred Reporting Items for Systematic Reviews and Meta-Analyses extension for Scoping Reviews
