# Supplementary material for: Development, Implementation, and Evaluation Methods for Dashboards in Health Care: Scoping Review
Source: JMIR Med Inform. 2024 Dec 10;12:e59828. doi: 10.2196/59828 (PMC11651422; doi:10.2196/59828)
Supplement: Multimedia Appendix 1 [file medinform-v12-e59828-s001.docx]

Dashboards in Healthcare Settings: A Scoping Review

Supplement 1. Study Codebook and Examples for Eligibility Assessment and Data Extraction

Contents

[Study Codebook 2](#_Toc165537913)

[Healthcare Setting Codes and Definitions 2](#_Toc165537914)

[Dashboard Purpose Codes and Definitions 3](#_Toc165537915)

[Intended End-User Codes and Definitions 5](#_Toc165537916)

[Adjunct Implementation Strategy Codes and Definitions 6](#_Toc165537917)

[Other Relevant Definitions 8](#_Toc165537918)

[Examples for Adjunct Implementation Strategies 10](#_Toc165537919)

[Examples for Visual Elements 13](#_Toc165537920)

[References 16](#_Toc165537921)

# Study Codebook

| Variable | Code | Codebook Definition |
| --- | --- | --- |
| Healthcare Setting Codes and Definitions | | |
| Healthcare Setting | Inpatient Setting[1] | Inpatient care is the treatment and/or care that is provided to a patient in a hospital or other institution and which requires at least one overnight stay. Inpatients have to go through the full admission procedure and occupy a bed in an inpatient department of the institution. |
| Healthcare Setting | Outpatient Clinic[2] | Outpatient care services are the health care services that are provided to patients who are not hospitalized for an overnight stay. The outpatient care services are delivered in different settings like a physician or therapist's office, a clinic, or a day surgery center. Outpatient care covers diagnosis, observation, treatment, and rehabilitation. It is usually provided to persons that are able to ambulate or walk about. |
| Healthcare Setting | Emergency Services[3] | Emergency department means the part of a licensed hospital facility open 24 hours a day to provide care for anyone in need of emergency treatment. Also includes any other site where emergency medical services are provided, such as ambulances. |
| Healthcare Setting | Imaging or Radiology Facility[4] | A medical imaging center is a facility staffed by board certified, fellowship trained radiologists who specialize in diagnostic and preventative medicine. A medical imaging center offers a variety of diagnostic scans and tools to help protect your health and detect disease at the earliest stages. A medical imaging center offers services such as X-Rays, MRIs, CT scans and PET scans. |
| Healthcare Setting | Operating Room or Surgical Suite | Dashboard used in an operating room or surgical suite where procedures are performed or to inform clinicians or leaders within surgical or anesthesia departments of outcomes or other process measures associated with care provided during surgical operations or other procedures. |
| Healthcare Setting | Clinical Laboratory[5] | Clinical laboratories are healthcare facilities providing a wide range of laboratory procedures which aid the physicians in carrying out the diagnosis, treatment, and management of patients. These laboratories are manned by medical technologists (clinical laboratory scientists) who are trained to perform various tests of samples of biological specimens collected from its patients. Most of the clinical laboratories are situated within or near hospital facilities to provide access to both physicians and their patients. |
| Healthcare Setting | Other Setting or Unclear | Dashboard was used in a healthcare setting not described or listed above or it was unclear what type of setting the dashboard was used in. |
| Healthcare Setting | Long-Term Care Facility[6]* | Long-term care usually involves provision of custodial and non-skilled care, such as assisting with normal daily tasks like dressing, bathing, and using the bathroom. This care component is often provided in combination with help with basic medical services such as help with wound dressing, pain management, medication, health monitoring, prevention, rehabilitation, or services of palliative care. Long-term care can be provided at home, in the community, in assisted living accommodation, or in nursing homes. |
| Healthcare Setting | Home or Community Care[7]* | Community care can be defined as the blend of health and social services provided to individuals or families at home or in the community for the purpose of promoting, maintaining or restoring health or minimizing the effects of illness and disability and thus achieving maximum independence and control over their own lives. The traditional main users of community care services are people that have a continuing need for care on a longer-term basis as a result of the effects of old age, mental illness, mental handicap or physical disability. The spectrum of community care ranges from domiciliary support provided at home or through sheltered housing and group homes within the community with increasing levels of care. |
| Healthcare Setting | Hospice[8]* | Hospice care is defined as a program that provides palliative care and attends to the emotional and spiritual needs of terminally ill patients at an inpatient facility or in the patient's home. Hospice care is most frequently provided in the patient's home, in long term care facilities or residential institutions. |
| Healthcare Setting | Dialysis Center[9]* | Freestanding dialysis centers provide chronic maintenance dialysis to end stage renal disease patients on an outpatient basis, including dialysis services in the patient's place of residence. A certified end stage renal disease facility is a facility that provides outpatient maintenance dialysis services, or home dialysis training and support services, or both. A dialysis center may be an independent or hospital-based unit. |
| Dashboard Purpose Codes and Definitions | | |
| Purpose - Clinical | Direct Patient Care[10] | A dashboard used when providing direct, immediate care to a patient in any healthcare setting. “Direct patient care is defined as hands on, face-to-face contact with patients for the purpose of diagnosis, treatment and monitoring.” |
| Purpose - Clinical | Population Health Management[11] | A dashboard used to identify patients in a clinicians panel or within a department or unit that are at risk for an adverse event and in need of intervention (i.e., dashboard identifies patients with potentially inappropriate prescribing or unsafe drug combinations with the aim of preventing the adverse event before it occurs). Population health is a general approach to assessing and managing health at the level of the whole population. Reduction of health-care inequities, prevention of illness, and contextual improvement are all central objectives of the population health approach. Rather than focusing on treating illnesses after they emerge, population health interventions are characterized by a strong emphasis on primary prevention, or preventing illness before it develops. As such, modifiable risk factors for illness are a common focus of population health intervention such as smoking, obesity, high blood pressure, physical inactivity, and unhealthy diet as well as preventable disease such as heart disease, stroke, certain cancers, and diabetes. |
| Purpose - Clinical | Care Coordination[12] | A dashboard that supports care coordination by pulling information from multiple data sources and allowing both the patient and a healthcare provider to view the dashboard, and/or allowing the patient to enter self-reported health data into the dashboard to complement EHR information for the clinician to use for care planning and decision-making. Care coordination involves deliberately organizing patient care activities and sharing information among all of the participants concerned with a patient's care to achieve safer and more effective care. This means that the patient's needs and preferences are known ahead of time and communicated at the right time to the right people, and that this information is used to provide safe, appropriate, and effective care to the patient. |
| Purpose – Non-Clinical | Performance Monitoring[13] | A dashboard that provides data on individual provider or unit/site level performance. These dashboards often show performance trends over time as well as offer the user the ability to compare their performance to that of peers or to averages within their department. |
| Purpose – Non-Clinical | Utilization Tracking[14] | A dashboard used to provide data on healthcare utilization, either at the level of the patient (e.g., how often they visit, how long visits take, where the patient is seen, etc.) or at the level of the department or organization (e.g., services per day/month/year, services by category or unit, top services provided by cost or in a given time period). “Health Care Utilization is the quantification or description of the use of services by persons for the purpose of preventing and curing health problems, promoting maintenance of health and well-being, or obtaining information about one’s health status and prognosis.” |
| Purpose – Non-Clinical | Resource Management[15,16] | A dashboard used to support resource management by providing data to support adequate staffing, ensure appropriate and adequate supplies are available, and monitor bed management and/or patient transfers. “Healthcare resource management refers to activities to direct and control the supply and use of the healthcare resources required to perform healthcare activities.” |
| Purpose – Non-Clinical | Financial Tracking[17] | A dashboard used to track healthcare key performance indicators (KPIs) such as costs of different visits or procedures in order to provide budget analysis support and insight into cost trends. “Healthcare finance KPIs are quantifiable measurements. They can help hospital finance and department leaders effectively gauge financial and operational performance against set goals. When used right, healthcare finance KPIs create successful internal measurements that allow for a comparison into what is and isn’t working within a practice. KPIs also lend visibility to trending regulatory compliance. A few of those KPI are governed for regulatory reporting so seeing them on a consistent basis can help raise flags earlier.” |
| Purpose – Non-Clinical | Facility Management[18] | These dashboards may be used to oversee construction or maintenance projects, maintain building functionality and safety (i.e., HVAC systems, gas, refrigeration), or support security efforts. “Healthcare facilities management is the maintenance and oversight of a healthcare facilities' development, maintenance, security and operations. This includes any building that provides healthcare services, such as clinics, long-term facilities, surgical centers and hospitals, and covers every aspect of facility upkeep and ranges from overseeing large construction projects and maintaining federal compliance down to everyday cleaning, maintenance and upkeep.” |
| Purpose – Non-Clinical | Education/Training | A dashboard used to support education or training of early career clinicians by supporting and facilitating feedback from supervisors or tracking metrics for ACGME compliance. |
| Purpose – Non-Clinical | Facilitate Clinical or Quality Registry Use | A dashboard that is used to facilitate and support use of clinical or quality registries to promote participation in these groups which monitor outcomes for specific populations to inform safe, effective care and improve adherence to evidence-based practices. |
| Purpose – Non-Clinical | BPA Tracking | A dashboard used for providing information on rates at which best practice advisories or pop-up alerts are generated and displayed to clinicians and whether the alerts are accepted or overridden to inform efforts to improve these decision support tools and reduce alert fatigue for clinicians. |
| Purpose – Other | Clinical Trial Support Tool | A dashboard used to support and facilitate clinical research activities either through identification of eligible participants for recruitment, monitoring of enrolled participants, or tracking scheduled research activities to ensure protocol compliance and adherence to study timelines. |
| Purpose – Subject to Interpretation | Other Purpose | Any dashboard that does not fit into a category described above. |
| Intended End-User Codes and Definitions | | |
| Intended End-User – Clinical | Front-Line Clinician | The end-user is a physician (MD, DO), advance practice provider (APP, [NP or PA]), nurse (RN, LPN), medical assistant, or other frontline clinical care staff member (e.g., respiratory therapist, radiology technician). |
| Intended End-User – Clinical | Pharmacist or Pharmacy Staff | The end-user is a licensed pharmacist, pharmacy technician or other pharmacy support staff member. |
| Intended End-User – Clinical | Patients | The end-user is a patient. Patients should only be considered as end-users if they are provided with data visualizations or feedback from the dashboard or an mhealth application linked to their healthcare providers dashboard. As patients are defined as end-users only when they receive clinical information or communicate with a provider through the dashboard, they will be considered clinical users. |
| Intended End-User – Clinical | Clinician Trainees | The end-user is a trainee clinician working under a licensed supervisor. This may include medical residents, medical fellows, nursing students, pharmacy students, and students in other programs that provide direct patient care (e.g., physical therapy, occupational therapy, etc.).  **Only use when dashboard is being targeted directly to residents/fellows/trainees to support education or development. If dashboard is used solely for clinical care and is available to non-residents, use “front line clinician” for end-user** |
| Intended End-User – Clinical | Remote Monitoring Staff | The end-user is a clinician who virtually or remotely monitors patient care activity and provides ongoing support or outreach to patients or their on-site clinical care teams as needed (e.g., cardiac rehabilitation monitors, transplant surgery pharmacists monitoring post-operative patients for adverse events). |
| Intended End-User – Clinical | Clinical Research Teams | The end-user is a clinical research coordinator, principal investigator or co-investigator, other research team member, or study sponsor using the dashboard for a specific clinical research project. |
| Intended End-User – Non-Clinical | Leaders and/or Administrators | The end-user is a manager, healthcare administrator, or in a leadership position in the healthcare setting where the dashboard is being used |
| Intended End-User – Non-Clinical | Quality Improvement (QI) Stakeholders | The end-user is someone using the dashboard solely as part of a quality improvement collaboration, is a member of a steering committee or workgroup, or is a project or implementation champion or coach working in the setting in which the dashboard is used. |
| Intended End-User – Subject to Interpretation | Support Staff | The end-user is a support staff member to a front line clinician. Includes clerks, schedulers, and other health care staff members not included in any lists above. |
| Intended End-User – Subject to Interpretation | Other Users | The end-user described does not fit into any above category **If selecting other, give more details about the intended end-user, if available, in the “intended end-user description” tab |
| Adjunct Implementation Strategy Codes and Definitions | | |
| Adjunct Implementation Strategy | Audit and provide feedback, or facilitate relay of clinical data[19] | - Collect and summarize clinical performance data over a specified time period and give it to clinicians and administrators to monitor, evaluate, and modify provider behavior - Provide as close to real-time data as possible about key measures of process/outcomes using integrated modes/channels of communication in a way that promotes use of the targeted innovation |
| Adjunct Implementation Strategy | Conduct educational sessions, or disseminate educational materials[19] | - Hold meetings targeted toward different stakeholder groups (e.g., providers, administrators, other organizational stakeholders, and community, patient/consumer, and family stakeholders) to teach them about the clinical innovation - Have a trained person meet with providers in their practice settings to educate providers about the clinical innovation with the intent of changing the provider’s practice - Develop and format manuals, toolkits, and other supporting materials in ways that make it easier for stakeholders to learn about the innovation and for clinicians to learn how to deliver the clinical innovation and distribute educational materials (including guidelines, manuals, and toolkits) in person, by mail, and/or electronically |
| Adjunct Implementation Strategy | Conduct a needs assessment, identify barriers and facilitators[19] | - Assess various aspects of an organization to determine its degree of readiness to implement, barriers that may impede implementation, and strengths that can be used in the implementation effort - Collect and analyze data related to the need for the innovation |
| Adjunct Implementation Strategy | Form advisory boards or workgroups[19] | - Recruit and cultivate relationships with partners in the implementation effort - Create and engage a formal group of multiple kinds of stakeholders to provide input and advice on implementation efforts and to elicit recommendations for improvements |
| Adjunct Implementation Strategy | Identify champions, involve local opinion leaders[19] | - Identify and prepare individuals who dedicate themselves to supporting, marketing, and driving through an implementation, overcoming indifference or resistance that the intervention may provoke in an organization - Inform providers identified by colleagues as opinion leaders or “educationally influential” about the clinical innovation in the hopes that they will influence colleagues to adopt it - Identify early adopters at the local site to learn from their experiences with the practice innovation |
| Adjunct Implementation Strategy | Mandate change, institute guidelines[19] | - Have leadership declare the priority of the innovation and their determination to have it implemented - Create new institutional guidelines that require clinicians or other stakeholders to engage in efforts, change practices, and/or use the dashboard or other project-specific tools |
|  | Change teams or professional roles[19] | - Change who serves on the clinical team, adding different disciplines and different skills to make it more likely that the clinical innovation is delivered (or is more successfully delivered) - Shift and revise roles among professionals who provide care, and redesign job characteristics |
| Adjunct Implementation Strategy | Change environment or record systems[19] | - Evaluate current configurations and adapt, as needed, the physical structure and/or equipment (e.g., changing the layout of a room, adding equipment) to best accommodate the targeted innovation **Could include placing a large monitor in the healthcare setting that displays dashboard to anyone in department, or moving medical equipment to increase response times in emergencies** - Change records systems to allow better assessment of implementation or clinical outcomes |
| Adjunct Implementation Strategy | Involve patients and families, prepare patients to be active in care[19] | - Engage or include patients/consumers and families in the implementation effort - Prepare patients/consumers to be active in their care, to ask questions, and specifically to inquire about care guidelines, the evidence behind clinical decisions, or about available evidence-supported treatments - Develop strategies to increase patient/consumer and family feedback on the implementation effort |
| Adjunct Implementation Strategy | Financial incentives or disincentives[19] | - Work to incentivize the adoption and implementation of the clinical innovation - Pay providers or care systems a set amount per patient/consumer for delivering clinical care - Provide financial disincentives for failure to implement or use the clinical innovations |
| Adjunct Implementation Strategy | Remind clinicians or other stakeholders[19] | - Develop reminder systems designed to help clinicians (or other stakeholders) to recall information and/or prompt them to use the clinical innovation |
| Adjunct Implementation Strategy | Other strategy reported | Any other strategies that are described as part of the overall development or implementation effort that may be likely to contribute to the overall success of the intervention (e.g., signed commitments or pledges from clinicians, translation of patient-facing materials to a second language to reduce patient-level barriers). |
| Other Relevant Definitions | | |
| Other Definitions | End-User[20] | The ultimate consumer of a finished product. For this study, the end-user is the individual who views the data on the dashboard and interprets the information provided by the dashboard. |
| Other Definitions | Formative Usability Testing[21,22] | Usability testing refers to evaluating a product or service by testing it with representative users. Typically, during a test, participants will try to complete typical tasks while observers watch, listen and takes notes. The goal is to identify any usability problems, collect qualitative and quantitative data and determine the participant's satisfaction with the product. Formative usability testing is considered to have been completed if the testing was done in a hands-on fashion during the formative stages of dashboard design or was solely done prior to use of the dashboard in routine practice with real data. Usability testing may consist of questionnaires, assessments of task completion, think-aloud demos, interviews or focus groups, or heuristic testing which involves use of guidelines to evaluate whether the dashboard is user-friendly. |
| Other Definitions | Delivery Channel[23] | The delivery channel refers to the environment where the audience or consumer of the content will discover and access it. Audiences, as the ultimate end-users of content, will rely on services, platforms, or devices to access content, which could be a web browser, a mobile app, a kiosk, or a speaker, among other options. |
| Other Definitions | Benchmark[24] | - A criterion of quality or service in health care, usually expressed as a measurable standard. - A reference standard or basis for comparison that serves as a definition of a norm. |

*Due to the small number of dashboards identified that were used in long-term care settings, home or community health care settings, hospice facilities, and dialysis centers, any dashboards used in these settings are represented with “other settings” in the main results.

# Examples for Adjunct Implementation Strategies

| Adjunct Implementation Strategy | Example | Source of Example |
| --- | --- | --- |
| Audit and provide feedback, or facilitate relay of clinical data | "In addition, trained NHS clinical pharmacists will visit the practices regularly for at least 12 weeks, access the dashboard, and advise on changes in medication prescribing/monitoring in collaboration with practice staff." | 706 – Williams 2018[25] |
| Conduct educational sessions, or disseminate educational materials | "General education sessions (rounds) provided R/O MI laboratory consensus standards during the initial planning period and introduced impending changes to order sets. The results of institutional discordant test pairs analysis and baseline unit compliance (Figures 1 and 2) were presented (in interrupted time sequence) to prospective peer leaders to facilitate their recruitment. Peer leader volunteers then used the baseline data and subsequent alterations in practice patterns to motivate peers, using figural dashboards." | 833 – Ducatman 2017[26] |
| Conduct a needs assessment, identify barriers and facilitators | "We first developed a programme theory for our intervention, seeking to identify the factors that would promote the use of timely data audit and feedback to facilitate behaviour change (‘intensive feedback’), as well as any potential barriers to the success of such an intervention (box 1)." | 648 – Patel 2018[27] |
| Form advisory boards or workgroups | "First, we joined a national bronchiolitis QI collaborative,12 and second gathered a CM bronchiolitis workgroup (October 2015)— including representatives from ED, hospitalists, nursing, respiratory therapy, information technology (IT), pharmacy, and critical care—to determine local QI interventions." | 956 – Hester 2019[28] |
|  | "HAC work groups were formed, each led by a clinical subject matter expert in partnership with a quality and process improvement specialist, front line staff, faculty, and parent partners. Each HAC team identified and approved the appropriate bundle elements and organized them into a standard bundle format (‍Fig 4) as a way for staff to leverage a consistent and reliable tool for evidence-based practice. " | 511 – Hyman 2017[29] |
| Identify champions, involve local opinion leaders | "At least 1 key leader was identified at each facility who was required to review the report, identify prescribing variation, and take appropriate action to enhance patient safety. These actions may include notifying providers and providing feedback to promote safe prescribing, while maintaining an individualized approach to treatments." | 482 – Lin 2017[30] |
| Mandate change, institute guidelines | "In February 2017 our fifth initiative introduced a formal policy eliminating the transfer of unused medications, including controlled substances, between patients. The policy stipulated that dispensed but unused medicines, even full vials, must be wasted rather than transferred to another patient." | 782 – Dolan 2019[31] |
| Mandate change, institute guidelines | "Finally, in collaboration with the CHSPS Network, CHCO adopted and implemented “bundles” of evidence enabled preventive care guidelines for the set of defined HACs. ….. Our organization adapted these bundle elements as practice recommendations and aligned organizational policies and procedures to them." | 511 – Hyman 2017[29] |
| Change teams or professional roles | "As the major regional referral laboratory for COVID-19, only a fraction of the COVID-19 testing performed was for established UW Medicine patients. The responsibility for communicating results is challenging for patients without an associated UW Medicine primary care provider to perform the follow-up. In response, UW Medicine implemented a centralized results notification process, which uses remote workers instead of front-line clinicians. All ambulatory results are routed to a centralized EHR inbox pool staffed by trained professionals...Another critical improvement was to give front desk staff the ability to access updated appointment templates and cancellation options…UW Medicine moved quickly to launch telemedicine capability in the ICUs, to allow intensivists to engage with patients via tele-ICU carts, rolling devices with two high definition cameras and a screen on a pole with wheels." | 966 – Grange 2020[32] |
| Change environment or record systems | "In August 2016 we began our second step—the development and placement of printed laminated signs on all the computers in the OR. These signs reminded providers to reconcile their documentation of medications administered with the documentation in the AMDS screen, before closing the EMR." | 792 – Dolan 2019[31] |
|  | "Fourth, (February 2016) we published a local modification of the AAP 2014 bronchiolitis guideline and a companion order-set to our intranet and EHR (Cerner)." | 956 – Hester 2019[28] |
|  | "Electronic Changes to Laboratory Test Orders - The EM peer leader and hospital administration including the chief medical information officer (CMIO) initially selected a minimally interruptive “soft stop” that changed a default order set that had included CKMB and CK. " | 833 – Ducatman 2017[26] |
| Involve patients and families, prepare patients to be active in care | "We convened our Patient Advisory Board, comprising the leaders of support groups we engaged in step 1, for a PD session to elicit prototype input. During the 2-hour PD session, we presented board members with dashboard priorities and preferences from step 1 that informed QOL Tracker. We stepped through prototype screenshots to illustrate how patients would use QOL Tracker to submit EPIC surveys and generate personalized dashboards. We then asked board members for input on visual formats, conveying data variability (eg, error bars or shaded ranges), and pictograph style for patients with limited graphic literacy52 (figure 3). Findings built a consensus on refinements to prepare QOL Tracker for heuristic evaluation." | 917 – Hartzler 2016[33] |
|  | "The current system was developed using an iterative process based on our previous research. Patient focus groups, surveys, and pilot studies were conducted and applied to develop a system that was patient centric, acceptable to patients, and could provide optimal comprehensive monitoring without being overly intrusive or difficult to use on a day-to-day basis. After preliminary focus group sessions, a prototype mHealth application was developed and demonstrated to 99 renal transplant recipients. ... We also conducted a 60- patient interventional pilot study to assess the benefit of using mHealth to monitor home-based blood pressure and glucose values." | 940 – Taber 2019[34] |
| Financial incentives or disincentives | "Physicians with >3 quarters of noncompliance were subject to decreased financial compensation at the department chair’s discretion, representing the apex of the intervention pyramid." | 1080 – Stachelek 2020[35] |
|  | “The sixth intervention involved linking physician’s compensation to QI initiatives. In March of 2017 anesthesiologists were notified that controlled substance documentation discrepancies would be part of a raft of QI and productivity targets tied to their at-risk compensation, with the potential for a 5% reduction of their existing compensation for greater than two controlled substance errors per quarter year. In July 2017 the intervention was implemented and up to the time of writing of this article no anesthesiologist exceeded the two-error threshold." | 792 – Dolan 2019[31] |
| Remind clinicians or other stakeholders | "Pre-intervention, sedation minimization, and ventilator liberation were reviewed daily during a multidisciplinary huddle. Post-intervention, the dashboard was used during the multidisciplinary huddle, throughout the day by respiratory therapists, and text alerts were sent to bedside providers.........In addition to the dashboard, text-message alerts are sent to bedside providers when patients newly meet SBT and SAT eligibility." | 1052 – Anderson 2019[36] |
|  | "In August 2016 we began our second step—the development and placement of printed laminated signs on all the computers in the OR. These signs reminded providers to reconcile their documentation of medications administered with the documentation in the AMDS screen, before closing the EMR." | 792 – Dolan 2019[31] |

# Examples for Visual Elements

| Dashboard Visual Element | Example | Source of Example |
| --- | --- | --- |
| Color Coding or “Traffic Light Coding” | 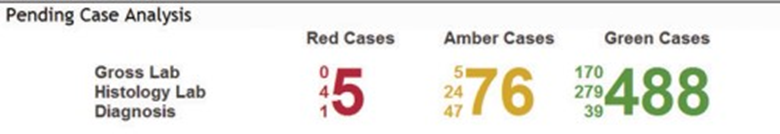 | 817 – Halwani 2016[37] |
|  | 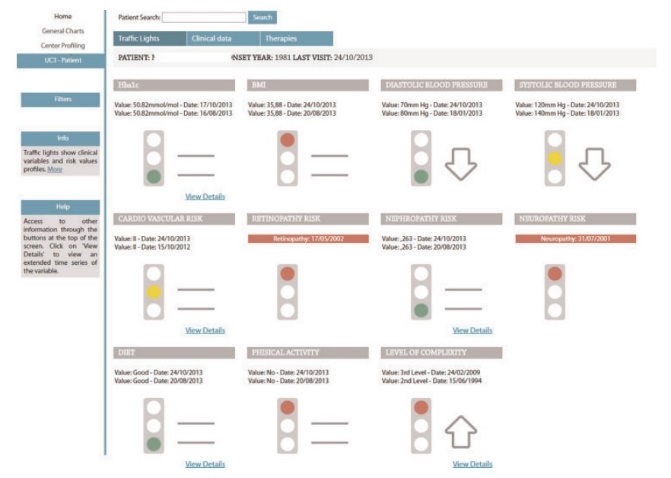 | 642 – Dagliati 2018[38] |
|  | 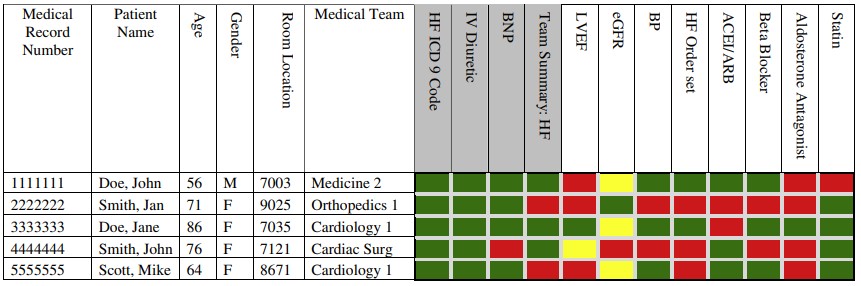 | 647 – McGough 2017[39] |
| Graphs | 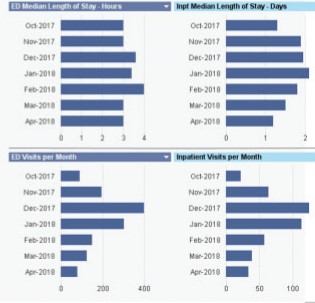 | 3721 – Burningham 2020[40] |
|  | 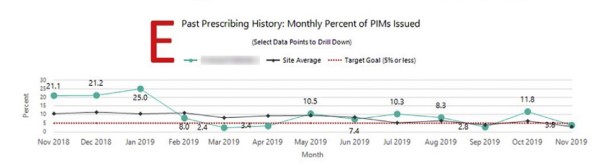 | 956 – Hester 2019[28] |
| Tables | 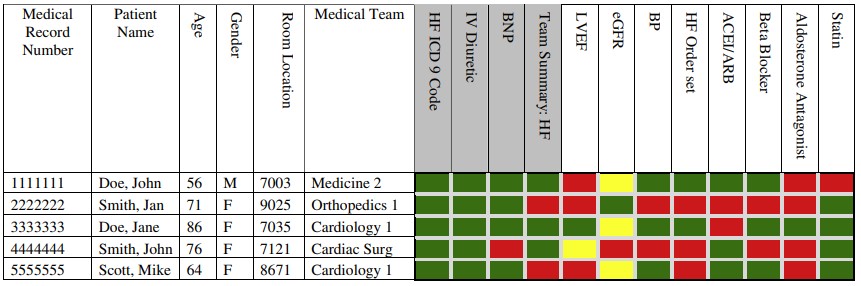 | 647 – McGough 2017[39] |
| Gauges | 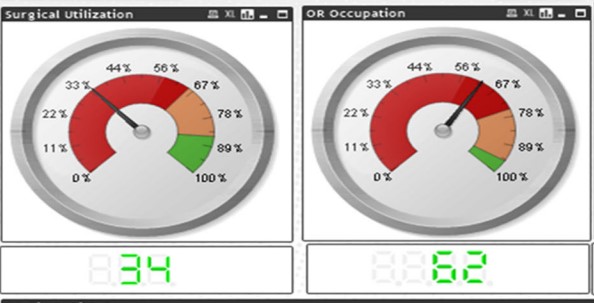 | 4027 – Nouei 2015[41] |
| Other | 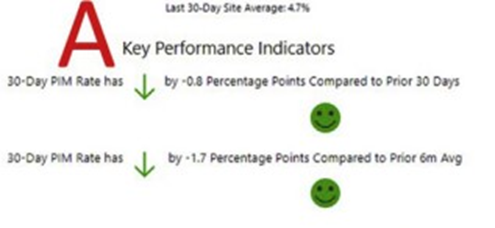 | 3721 – Burningham 2020[40] |

# References

1. Lenz G. Inpatient CareInpatient care. In: Kirch W, editor. Encyclopedia of Public Health Dordrecht: Springer Netherlands; 2008. p. 793–795. doi: 10.1007/978-1-4020-5614-7_1824

2. Lenz G. Outpatient CareOutpatient care. In: Kirch W, editor. Encyclopedia of Public Health Dordrecht: Springer Netherlands; 2008. p. 1068–1070. doi: 10.1007/978-1-4020-5614-7_2486

3. Emergency department Definition. Law Insider. Available from: https://www.lawinsider.com/dictionary/emergency-department [accessed May 5, 2022]

4. What is a medical imaging center? Medical Imaging of Fredericksburg. 2017. Available from: https://mifimaging.com/2017/11/12/what-is-a-medical-imaging-center/ [accessed May 5, 2022]

5. Bayot ML, Brannan GD, Naidoo P. Clinical Laboratory. StatPearls Treasure Island (FL): StatPearls Publishing; 2022. PMID:30570979

6. Lenz G. Long-Term CareLong-term care. In: Kirch W, editor. Encyclopedia of Public Health Dordrecht: Springer Netherlands; 2008. p. 865–867. doi: 10.1007/978-1-4020-5614-7_2009

7. Lenz G. Community CareCommunity care. In: Kirch W, editor. Encyclopedia of Public Health Dordrecht: Springer Netherlands; 2008. p. 139–141. doi: 10.1007/978-1-4020-5614-7_473

8. Kirch W, editor. Hospice CareHospice care. Encyclopedia of Public Health Dordrecht: Springer Netherlands; 2008. p. 682–682. doi: 10.1007/978-1-4020-5614-7_1562

9. Freestanding Dialysis Centers | Ohio Department of Health. Available from: https://odh.ohio.gov/know-our-programs/end-stage-renal-disease-dialysis/freestandingdialysiscenters [accessed May 5, 2022]

10. Centers for Disease Control and Prevention. NHSN Healthcare Personnel Safety Component Key Terms. 2013. Available from: https://www.cdc.gov/nhsn/pdfs/hps-manual/exposure/6-hps-key-terms.pdf

11. Zehr C, Hall PA. Population Health. In: Gellman MD, Turner JR, editors. Encyclopedia of Behavioral Medicine New York, NY: Springer; 2013. p. 1504–1506. doi: 10.1007/978-1-4419-1005-9_1169

12. Agency for Healthcare Research and Quality, Rockville, MD. Care Coordination. Care Coordination. 2018. Available from: https://www.ahrq.gov/ncepcr/care/coordination.html [accessed May 5, 2022]

13. Day DV, Gronn P, Salas E. Leadership capacity in teams. The Leadership Quarterly 2004 Dec 1;15(6):857–880. doi: 10.1016/j.leaqua.2004.09.001

14. Carrasquillo O. Health Care Utilization. In: Gellman MD, Turner JR, editors. Encyclopedia of Behavioral Medicine New York, NY: Springer; 2013. p. 909–910. doi: 10.1007/978-1-4419-1005-9_885

15. Olsen JC. What is resource management? J Healthc Resour Manag 1995 Jan;13(1):11–13. PMID:10142824

16. Healthcare Resource Management. Available from: https://contsys.org/concept/healthcare_resource_management [accessed May 5, 2022]

17. Multiview Financial Software. Healthcare Finance KPIs You Should Be Tracking. 2022. Available from: https://multiviewcorp.com/blog/healthcare-finance-kpis-you-should-be-tracking [accessed May 5, 2022]

18. Accruent. What is Healthcare Facilities Management (HFM)? Available from: https://www.accruent.com/resources/blog-posts/what-healthcare-facilities-management-hfm [accessed May 5, 2022]

19. Powell BJ, Waltz TJ, Chinman MJ, Damschroder LJ, Smith JL, Matthieu MM, Proctor EK, Kirchner JE. A refined compilation of implementation strategies: results from the Expert Recommendations for Implementing Change (ERIC) project. Implementation Science 2015 Feb 12;10(1):21. doi: 10.1186/s13012-015-0209-1

20. End user Definition & Meaning - Merriam-Webster. Available from: https://www.merriam-webster.com/dictionary/end%20user [accessed May 5, 2022]

21. Affairs AS for P. Usability Testing. Department of Health and Human Services; 2013. Available from: https://www.usability.gov/how-to-and-tools/methods/usability-testing.html [accessed May 5, 2022]

22. Maramba I, Chatterjee A, Newman C. Methods of usability testing in the development of eHealth applications: A scoping review. Int J Med Inform 2019 Jun;126:95–104. PMID:31029270

23. Delivery channel | Glossary. Kontent by Kentico. Available from: https://kontent.ai/glossary/delivery-channel/ [accessed May 5, 2022]

24. benchmark. The Free Dictionary. Available from: https://medical-dictionary.thefreedictionary.com/benchmark [accessed May 5, 2022]

25. Williams R, Keers R, Gude WT, Jeffries M, Davies C, Brown B, Kontopantelis E, Avery AJ, Ashcroft DM, Peek N. SMASH! The Salford medication safety dashboard. Journal of innovation in health informatics England; 2018;25(3):183–193. doi: 10.14236/jhi.v25i3.1015

26. Ducatman AM, Tacker DH, Ducatman BS, Long D, Perrotta PL, Lawther H, Pennington K, Lander O, Warden M, Failinger C, Halbritter K, Pellegrino R, Treese M, Stead JA, Glass E, Cianciaruso L, Nau KC. Quality Improvement Intervention for Reduction of Redundant Testing. Academic pathology United States; 2017;4(101698648):2374289517707506. doi: 10.1177/2374289517707506

27. Patel S, Rajkomar A, Harrison JD, Prasad PA, Valencia V, Ranji SR, Mourad M. Next-generation audit and feedback for inpatient quality improvement using electronic health record data: a cluster randomised controlled trial. BMJ quality & safety England; 2018;27(9):691–699. doi: 10.1136/bmjqs-2017-007393

28. Hester G, Lang T, Madsen L, Tambyraja R, Zenker P. Timely Data for Targeted Quality Improvement Interventions: Use of a Visual Analytics Dashboard for Bronchiolitis. Applied clinical informatics Germany; 2019;10(1):168–174. doi: 10.1055/s-0039-1679868

29. Hyman D, Neiman J, Rannie M, Allen R, Swietlik M, Balzer A. Innovative Use of the Electronic Health Record to Support Harm Reduction Efforts. Pediatrics United States; 2017;139(5). doi: 10.1542/peds.2015-3410

30. Lin LA, Bohnert ASB, Kerns RD, Clay MA, Ganoczy D, Ilgen MA. Impact of the Opioid Safety Initiative on opioid-related prescribing in veterans. Pain United States; 2017;158(5):833–839. doi: 10.1097/j.pain.0000000000000837

31. Dolan JE, Lonsdale H, Ahumada LM, Patel A, Samuel J, Jalali A, Peck J, DeRosa JC, Rehman M, Varughese AM, Fernandez AM. Quality Initiative Using Theory of Change and Visual Analytics to Improve Controlled Substance Documentation Discrepancies in the Operating Room. Applied clinical informatics Germany; 2019;10(3):543–551. doi: 10.1055/s-0039-1693688

32. Grange ES, Neil EJ, Stoffel M, Singh AP, Tseng E, Resco-Summers K, Fellner BJ, Lynch JB, Mathias PC, Mauritz-Miller K, Sutton PR, Leu MG. Responding to COVID-19: The UW Medicine Information Technology Services Experience. Applied clinical informatics Germany; 2020;11(2):265–275. doi: 10.1055/s-0040-1709715

33. Hartzler AL, Izard JP, Dalkin BL, Mikles SP, Gore JL. Design and feasibility of integrating personalized PRO dashboards into prostate cancer care. Journal of the American Medical Informatics Association : JAMIA England; 2016;23(1):38–47. doi: 10.1093/jamia/ocv101

34. Taber DJ, Pilch NA, McGillicuddy JW, Mardis C, Treiber F, Fleming JN. Using informatics and mobile health to improve medication safety monitoring in kidney transplant recipients. American journal of health-system pharmacy : AJHP : official journal of the American Society of Health-System Pharmacists England; 2019;76(15):1143–1149. doi: 10.1093/ajhp/zxz115

35. Stachelek GC, McNutt T, Thompson CB, Smith K, DeWeese TL, Song DY. Improvements in Physician Clinical Workflow Measures After Implementation of a Dashboard Program. Practical radiation oncology United States; 2020;10(3):151–157. doi: 10.1016/j.prro.2019.11.014

36. Anderson BJ, Do D, Chivers C, Choi K, Gitelman Y, Mehta SJ, Panchandam V, Gudowski S, Pierce M, Cereda M, Christie JD, Schweickert WD, Gabrielli A, Huffenberger A, Draugelis M, Fuchs BD. Clinical Impact of an Electronic Dashboard and Alert System for Sedation Minimization and Ventilator Liberation: A Before-After Study. Critical care explorations United States; 2019;1(10):e0057. doi: 10.1097/CCE.0000000000000057

37. Halwani F, Li WC, Banerjee D, Lessard L, Amyot D, Michalowski W, Giffen R. A real-time dashboard for managing pathology processes. Journal of pathology informatics India; 2016;7(101528849):24. doi: 10.4103/2153-3539.181768

38. Dagliati A, Sacchi L, Tibollo V, Cogni G, Teliti M, Martinez-Millana A, Traver V, Segagni D, Posada J, Ottaviano M, Fico G, Arredondo MT, De Cata P, Chiovato L, Bellazzi R. A dashboard-based system for supporting diabetes care. Journal of the American Medical Informatics Association : JAMIA England; 2018;25(5):538–547. doi: 10.1093/jamia/ocx159

39. McGough P., Kline S., Simpson L. Team care approach to population health and care management. International Journal of Health Governance 2017;22(2):93–103. doi: 10.1108/IJHG-11-2016-0048

40. Burningham Z, Jackson G, Kelleher J, Stevens M, Morris I, Cohen J, Maloney G, Vaughan C. The Enhancing Quality of Prescribing Practices for Older Veterans Discharged From the Emergency Department (EQUIPPED) Potentially Inappropriate Medication Dashboard: a Suitable Alternative to the In-Person Academic Detailing and Standardized Feedback Rep. 2020; doi: 10.1016/j.clinthera.2020.02.013

41. Nouei MT, Kamyad AV, Soroush AR, Ghazalbash S. A comprehensive operating room information system using the Kinect sensors and RFID. J Clin Monitor Comp 2015;29(2):251–261. doi: 10.1007/s10877-014-9591-5
