# Supplementary material for: Development, Implementation, and Evaluation Methods for Dashboards in Health Care: Scoping Review
Source: JMIR Med Inform. 2024 Dec 10;12:e59828. doi: 10.2196/59828 (PMC11651422; doi:10.2196/59828)
Supplement: Multimedia Appendix 2 [file medinform-v12-e59828-s002.docx]

Dashboards in Healthcare Settings: A Scoping Review

Supplementary Tables

Contents

[Supplementary Table 1. Dashboard Purpose Responses Mapped to Purpose Groups 2](#_Toc178158234)

[Supplementary Table 2. End-Users Responses Mapped to End-User Groups 4](#_Toc178158235)

[Supplementary Table 3. Geographic Location of Healthcare Dashboards by Country 6](#_Toc178158236)

[Supplementary Table 4. Cross Tabulation of Dashboard Purpose Group and User Group 7](#_Toc178158237)

[Supplementary Table 5. Software or Coding Frameworks Used to Develop and Implement Healthcare Dashboards 8](#_Toc178158238)

[Supplementary Table 6. All Reported Combinations of Dashboard Delivery Channels 13](#_Toc178158239)

[Supplementary Table 7. How End-Users were Involved in the Design, Implementation, or Evaluation of Healthcare Dashboards 14](#_Toc178158240)

[Supplementary Table 8. Reports of Formative Usability Testing 23](#_Toc178158241)

[Supplementary Table 9. Dashboard Metrics Informed by Payor, Government, or Professional Guideline Standards 27](#_Toc178158242)

[Supplementary Table 10. Theoretical Frameworks Used to Guide Design, Implementation, or Evaluation of Healthcare Dashboards* 40](#_Toc178158243)

[References 43](#_Toc178158244)

# Supplementary Table 1. Dashboard Purpose Responses Mapped to Purpose Groups

| Dashboard Purpose(s) Reported | Purpose Group – n (% of column total) | | | |
| --- | --- | --- | --- | --- |
|  | Administrative (n=54) | Both Administrative  and Clinical (n=20) | Clinical (n=43) | Research (n=1) |
| Performance monitoring (n=20) | 20 (37.0%) |  |  |  |
| Care coordination, Direct patient care (n=16) |  |  | 16 (37.2%) |  |
| Population management, Direct patient care (n=14) |  |  | 14 (32.6%) |  |
| Utilization tracking, Performance monitoring (n=12) | 12 (22.2%) |  |  |  |
| Resource management, Utilization tracking (n=5) | 5 (9.3%) |  |  |  |
| Population management, Performance monitoring (n=5) |  | 5 (25.0%) |  |  |
| Resource management, Utilization tracking, Performance monitoring (n=5) | 5 (9.3%) |  |  |  |
| Population management (n=5) |  |  | 5 (11.6%) |  |
| Direct patient care (n=3) |  |  | 3 (7.0%) |  |
| Resource management (n=3) | 3 (5.6%) |  |  |  |
| Population management, Care coordination (n=3) |  |  | 3 (7.0%) |  |
| Resource management, Performance monitoring (n=2) | 2 (3.7%) |  |  |  |
| Population management, Direct patient care, Utilization tracking (n=2) |  | 2 (10.0%) |  |  |
| Financial tracking, Performance monitoring (n=2) | 2 (3.7%) |  |  |  |
| Performance monitoring, support training and education (n=2) | 2 (3.7%) |  |  |  |
| Direct patient care, Resource management, Utilization tracking (n=2) |  | 2 (10.0%) |  |  |
| Population management, Care coordination, Direct patient care (n=2) |  |  | 2 (4.6%) |  |
| Population management, Direct patient care, Resource management (n=2) |  | 2 (10.0%) |  |  |
| Population management, Utilization tracking, Performance monitoring (n=1) |  | 1 (5.0%) |  |  |
| Care coordination, Direct patient care, Facilitate and support use of clinical quality registries (n=1) |  | 1 (5.0%) |  |  |
| Facilitate and support use of clinical quality registries (n=1) | 1 (1.8%) |  |  |  |
| Direct patient care, Utilization tracking, Performance monitoring (n=1) |  | 1 (5.0%) |  |  |
| Population management, Resource management (n=1) |  | 1 (5.0%) |  |  |
| Financial tracking, Resource management (n=1) | 1 (1.8%) |  |  |  |
| Clinical trial screening or recruitment (n=1) |  |  |  | 1 (100.0%) |
| Monitor firing of and response to interruptive alerts (n=1) | 1 (1.8%) |  |  |  |
| Direct patient care, Resource management (n=1) |  | 1 (5.0%) |  |  |
| Population management, Financial tracking, Utilization tracking (n=1) |  | 1 (5.0%) |  |  |
| Direct patient care, Utilization tracking (n=1) |  | 1 (5.0%) |  |  |
| Direct patient care, Monitor firing of and response to interruptive alerts (n=1) |  | 1 (5.0%) |  |  |
| Population management, Direct patient care, Performance monitoring (n=1) |  | 1 (5.0%) |  |  |

# Supplementary Table 2. End-Users Responses Mapped to End-User Groups

| End-User(s) Reported | End-User Group – n (% of column total) | | | |
| --- | --- | --- | --- | --- |
|  | Non-Clinical (n=9) | Both Non-Clinical and Clinical (n=42) | Clinical (n=65) | Not reported (n=2) |
| Front-line clinical staff (n=39) |  |  | 39 (60.0%) |  |
| Front-line clinical staff, Leadership or management (manager, administrator) (n=28) |  | 28 (66.7%) |  |  |
| Front-line clinical staff, Patients (n=10) |  |  | 10 (15.4%) |  |
| Leadership or management (manager, administrator) (n=6) | 6 (66.7%) |  |  |  |
| Clinician – Pharmacist (n=3) |  |  | 3 (4.6%) |  |
| Front-line clinical staff, Clinician - Pharmacist (n=3) |  |  | 3 (4.6%) |  |
| Front-line clinical staff, Leadership or management (manager, administrator), Clinician - Pharmacist (n=3) |  | 3 (7.2%) |  |  |
| Clinician - Pharmacist, Leadership or management (manager, administrator) (n=2) |  | 2 (4.8%) |  |  |
| Not reported (n=2) |  |  |  | 2 (100.0%) |
| Front-line clinical staff, Leadership or management (manager, administrator), Other (n=2) |  | 2 (4.8%) |  |  |
| Front-line clinical staff, Remote monitoring staff (n=2) |  |  | 2 (3.1%) |  |
| Front-line clinical staff, Clinician - Trainee (Resident or fellow) (n=2) |  |  | 2 (3.1%) |  |
| Front-line clinical staff, Support staff (schedulers, clerks), Leadership or management (manager, administrator) (n=2) |  | 2 (4.8%) |  |  |
| Leadership or management (manager, administrator), Other (n=1) | 1 (11.1%) |  |  |  |
| Clinical research teams (n=1) |  |  | 1 (1.5%) |  |
| QI stakeholders (n=1) | 1 (11.1%) |  |  |  |
| Front-line clinical staff, Leadership or management (manager, administrator), QI stakeholders (n=1) |  | 1 (2.4%) |  |  |
| Leadership or management (manager, administrator), QI stakeholders (n=1) | 1 (11.1%) |  |  |  |
| Front-line clinical staff, Clinical Research Teams (n=1) |  |  | 1 (1.5%) |  |
| Clinician - Trainee (Resident or fellow), Leadership or management (manager, administrator) (n=1) |  | 1 (2.4%) |  |  |
| Front-line clinical staff, Leadership or management (manager, administrator), Patients, Other (n=1) |  | 1 (2.4%) |  |  |
| Remote monitoring staff (n=1) |  |  | 1 (1.5%) |  |
| Leadership or management (manager, administrator), Remote monitoring staff (n=1) |  | 1 (2.4%) |  |  |
| Clinician - Trainee (Resident or fellow) (n=1) |  |  | 1 (1.5%) |  |
| Front-line clinical staff, Clinician - Trainee (Resident or fellow), Leadership or management (manager, administrator) (n=1) |  | 1 (2.4%) |  |  |
| Front-line clinical staff, Patients, Other (n=1) |  |  | 1 (1.5%) |  |
| Front-line clinical staff, Clinician - Pharmacist, Other (n=1) |  |  | 1 (1.5%) |  |

# Supplementary Table 3. Geographic Location of Healthcare Dashboards by Country

| **Geographic Location** | **Dashboards**  (n=118) |
| --- | --- |
| **North America** | **79 (66.9%)** |
| *US – Non-VA* | 69 (58.7%) |
| *US – VA* | 7 (5.9%) |
| *Canada* | 3 (2.5%) |
| *US – Non-VA and Canada* | 0 (0.0%) |
| **South America** | **0 (0.0%)** |
| *Colombia* | 0 (0.0%) |
| *Argentina* | 0 (0.0%) |
| **Europe** | **18 (15.2%)** |
| *United Kingdom* | 8 (6.8%) |
| *The Netherlands* | 4 (3.4%) |
| *Italy* | 1 (0.8%) |
| *France* | 1 (0.8%) |
| *Norway* | 1 (0.8%) |
| *Ireland* | 0 (0.0%) |
| *Denmark* | 2 (1.7%) |
| *Sweden* | 1 (0.8%) |
| **Asia** | **11 (9.3%)** |
| *Iran* | 2 (1.7%) |
| *India* | 2 (1.7%) |
| *Taiwan* | 2 (1.7%) |
| *South Korea* | 2 (1.7%) |
| *Bangladesh* | 1 (0.8%) |
| *Japan* | 1 (0.8%) |
| *Oman* | 1 (0.8%) |
| **Africa** | **6 (5.1%)** |
| *South Africa* | 3 (2.5%) |
| *Nigeria* | 1 (0.8%) |
| *Namibia* | 1 (0.8%) |
| *Malawi* | 0 (0.0%) |
| *Mali* | 1 (0.8%) |
| ***Australia or New Zealand*** | **4 (3.4%)** |
| *Australia* | 4 (3.4%) |

# Supplementary Table 4. Cross Tabulation of Dashboard Purpose Group and User Group

|  | **End-User Group – n (% of column)** | | | |
| --- | --- | --- | --- | --- |
| **Purpose Group** | **Non-Clinical (n=9)** | **Both Non-Clinical and Clinical (n=42)** | **Clinical (n=65)** | **Not reported (n=2)** |
| Administrative (n=54) | 8 (88.9%) | 28 (66.7%) | 16 (24.6%) | 2 (100.0%) |
| Both Administrative and Clinical (n=20) | 1 (11.1%) | 10 (23.8%) | 9 (13.9%) | 0 (0.0%) |
| Clinical (n=43) | 0 (0.0%) | 4 (9.5%) | 39 (60.0%) | 0 (0.0%) |
| Research (n=1) | 0 (0.0%) | 0 (0.0%) | 1 (1.5%) | 0 (0.0%) |

# Supplementary Table 5. Software or Coding Frameworks Used to Develop and Implement Healthcare Dashboards

| Study Author, Publication Year – Study ID [Reference] | Software Grouping | Software Details |
| --- | --- | --- |
| Burns, 2020 - 1053 [62] | Custom Build | “This runs a Ruby on Rails application to query recent exam records in the SQL Server, analyze and/or aggregate the retrieved data, format it, and pass the results to the user’s web browser. Ancillary resources used to construct the web interface include HTML5, Cascading Style Sheets (CSS), JavaScript , NGINX, and Passenger.” |
| Laurent, 2020 – 1172 [30] | Custom Build | "The web interface was implemented using HTML, CSS and JavaScript, while PHP and Oracle were used on the server side. The visualization of the dashboards was rendered using Chart.js and D3.js libraries...The application was implemented on an Apache web server [37] running on a secure, private virtual server (Windows Server 2012 R2 Datacenter Edition)." |
| Burningham, 2020 – 3721 [7] | Custom Build | “The SQL Server Agent was used to automate and schedule the EQUIPPED dashboard ELT process for repeated execution. SQL Server Reporting Services was used in producing the user interface, performance measures, and required visuals and in adding interactive functionality to the dashboard. Data were extracted from the VA CDW." |
| Choi, 2018 – 4899 [63] | Custom Build | "The foundation of this system is our departmental server which receives real-time Health Level 7 (HL7) order and report data from our radiology information system (RIS) (Siemens). Data is filtered by a Mirth Connect HL7 engine (Mirth Corporation) and stored in a MySQL (Oracle) database with associated metadata. This server also includes an Apache web server (Apache Software Foundation) to receive feedback data and for display of a dashboard for feedback consumption." |
| Durojaiye, 2018 – 686 [64] | Custom Build | "The back end of the dashboard was developed in the Ruby programming language using the Rails framework, which connected to a PostgreSQL database. The front end was developed using Hypertext Markup Language (HTML) 5.0, Cascading Style Sheets (CSS) 3.0, and JavaScript. These three are all open Web standards. Bootstrap (21), UI-Bootstrap (22), and Bootstrap-Select(23) libraries were used to style the Web pages, while AngularJS (24) was used to provide interactivity and enable asynchronous communication with the server. Spin.js (25) was used as the progress indicator during communication with the server. Easy Pie Chart (26), Angular-Chart (27), Chart.js (28), and Chart-Horizontal.js (29) JavaScript charting libraries were used for data visualization. All of these libraries are free and open source. The dashboard was hosted on a GlassFish Web server that is part of the Analytical Informatics platform and is accessible on the institution’s network to all radiology residents and the program director." |
| Williams, 2018 – 706 [56] | Custom Build | “The system is deployed to two servers (see Figure 1), running Windows Server 2012, in the secure data centre of the SRFT. One server runs the main patient database using SQL Server 2012, and the other runs SMASH â€“ the web application that the users access. SMASH is rendered on the client side with AngularJs,[31] and is served with data via a RESTful API running on NodeJS[32] and Express.[33]” |
| Jameie, 2019 – 728 [27] | Custom Build | “The web-based dashboard was written using the ASP.Net framework and C# programming language. The android application was implemented in Java using Android Studio, version 1.3.2.” |
| Woo, 2019 - 884a [57] | Custom Build | “The dashboards run on an Apache web server which is part of a technology stack that utilizes Hypertext Preprocessor (PHP). There are numerous embedded functions that are provided within the PHP language to allow for data extrapolation from various databases. In the case of the dashboards, data are extracted in real time from the Sunquest (v7.3) Laboratory Information Systems Database (InterSystems Cache) via Open Database Connectivity and are refreshed every 2 min. Data are queried from Sunquest using Structured Query Language, the results of which are processed using PHP and rendered in a browser in the form of a dashboard, using Hypertext Markup Language for document definition and Cascading Style Sheets for display presentation. Data are hosted internally by our institution’s web servers and are accessed on a webpage via Microsoft Group Policy shortcuts. Secure Sockets Layer (SSL) certificates were installed on the web server to secure data both at rest and when in transit to client workstations.” |
| Woo, 2019 - 884b [57] | Custom Build | “The dashboards run on an Apache web server which is part of a technology stack that utilizes Hypertext Preprocessor (PHP). There are numerous embedded functions that are provided within the PHP language to allow for data extrapolation from various databases. In the case of the dashboards, data are extracted in real time from the Sunquest (v7.3) Laboratory Information Systems Database (InterSystems Cache) via Open Database Connectivity and are refreshed every 2 min. Data are queried from Sunquest using Structured Query Language, the results of which are processed using PHP and rendered in a browser in the form of a dashboard, using Hypertext Markup Language for document definition and Cascading Style Sheets for display presentation. Data are hosted internally by our institution’s web servers and are accessed on a webpage via Microsoft Group Policy shortcuts. Secure Sockets Layer (SSL) certificates were installed on the web server to secure data both at rest and when in transit to client workstations.” |
| Giordanengo, 2019 – 897 [22] | Custom Build | “The dashboard was then built to achieve the objectives described in the scenarios. An agile development process [32] was exclusively used for this task, as evolution, changes, and adaptability were necessary, considering the continuous inputs provided by the workshops. The implementation relied on Java Enterprise Edition 8, Java Server Faces 2.2, and Glassfish 5.” |
| Barbeito, 2019 – 1020 [3] | Tableau | “We used commercially available analytics and data visualization software (Tableau Desktop Professional 10.0, Seattle, WA, USA) to create 4 interactive dashboard prototypes…These dashboard prototypes were iteratively refined based on informal feedback provided by prospective users and made available to each anesthesiology attending provider through the Duke Anesthesiology Department’s intranet webpage.” |
| Tan, 2020 – 1022 [65] | Tableau | “At NYULH, a clinical dashboard was created utilizing Tableau software (Version 2019.2.2) [28] to track the frequency in which each alert fires as well as the number of consults that are ordered as a result of each distinct alert at a departmental level.” |
| Dixit, 2020 – 1187 [12] | Tableau | “Dashboards were developed using Tableau (Version 2020.1) was connected to the relational database.” |
| Kunjan, 2019 – 4820 [29] | Tableau | “A high fidelity dashboard prototype, using sample EHR data from the study CHC, was built using Tableau – a popular BI/data visualization software platform.” |
| Robinson, 2018 – 654 [66] | Tableau | “The intervention was real-time feedback of OR supply cost data to individual surgeons via automated dashboards and monthly reports. An automated Tableau dashboard was created, which utilized data extracted from a “point of use (POU)” cost accounting system and database.” |
| Tyler, 2018 – 663 [52] | Tableau | “The final dashboard, which was built in Tableau version 8.1 (Tableau Software, Seattle, WA), was accessible to all members of the project team via the hospital Intranet.” |
| Huber, 2018 – 665 [67] | Tableau | “We sought to develop an interactive dashboard using a commercially available data visualization tool, Tableau (Tableau, Seattle, WA), so that we could monitor the function of our commercial CDS, assess its clinical impact, and determine potential areas in need of quality improvement…In order to facilitate interaction with these data, a dashboard was created using the Tableau software platform. Tableau is a cloud-based analytics platform used for visualization of relational databases and multidimensional arrays.” |
| Martinez, 2018 – 761 [32] | Tableau | “The e-Dashboard is distributed to the stakeholders using three mechanisms: viewing in meetings when projected, receiving in email form, and signing on to our institutional website (Tableau Software).” |
| Dolan, 2019 – 792 [13] | Tableau | “Beginning in November 2017 the QI project team developed an interactive dashboard using the Tableau business intelligence tool.” |
| Hagaman, 2018 – 794 [68] | Tableau | “For phase 2, an Ongoing Professional Performance Evaluation (OPPE) dashboard was created with Tableau 8.2 (Tableau Software, Seattle, WA) that automatically tracked individual clinician adherence (Figure 1).” |
| Chaparro, 2020 - 1028 [9] | Qlikview | “We implemented a QlikView (Qlik Technologies Inc., King of Prussia, Pennsylvania, United States) visualization tool that displays an interactive dashboard of all BPAs firing within our institution for a rolling 6-month window.” |
| Patel, 2018 – 648 [69] | Qlikview | “The raw data were processed and cleaned automatically on a secure server and then visualised with a QlikView dashboard (Qlik, Radnor, Pennsylvania) hosted by the medical centre.” |
| Connor, 2018 – 744 [70] | Qlikview | “A hospital dashboard was created using a data analytics platform (QlikView, QlikTech International AB) to monitor all orders for blood products placed via the UW Health Systems electronic medical record, Health Link, from October 1, 2015, forward.” |
| Nelson, 2019 – 977 [36] | Qlikview | “A visual analytics interface (Qlikview, QlikTech, Radnor, Pennsylvania, United States) was built to aggregate data from all anesthesia encounters in pediatric radiation oncology at the Children’s Hospital of Philadelphia.” |
| Palin, 2020 – 1115 [38] | R – Shiny Package | "The infrastructure comprises one server for the import of the data and the use of a Virtual Desktop Infrastructure for the analyses, as well as a server to host a web-based dashboard for general practices to log on to (via a self-hosted authentication provider19) and view their practice’s results. Following analyses, summary results were stored in a document oriented database on the HSCN web application server for exclusive use of the data visualizations. The data visualizations were developed using the R Studio 2020 package Shiny, and the ‘Shiny Modules’ design pattern was used to scale from single visualization pages to multi-visualization pages" |
| Van Zijl, 2018 – 697 [71] | R – Shiny Package | “A web-based dashboard was programmed with shiny:Web Application Framework for R, allowing real-time outcome analysis when new patients were included in the data set.” |
| Cassim, 2020 – 1085 [8] | Microsoft Excel + Microstrategy Desktop | "The final temporary table was exported as a Microsoft Excel (Redmond, Washington, United States) worksheet and imported into the MicroStrategy Desktop analytics tool (Providence, Virginia, United States)." |
| Cassim, 2020 – 1086 [72] | Microsoft Excel + Microstrategy Desktop | “Initially, the three measures described above were reported in Microsoft Excel (Redmond, Washington, United States) worksheet format from August 2016 to June 2017.4 Thereafter, from July 2017, an interactive dashboard was developed that reported TAT data for a basket of tests using the Microstrategy Desktop (Tysons, Virginia, United States) analytics tool” |
| Niendam, 2018 – 629 [73] | Ginger.io | "We utilized Ginger.io (Ginger.io, v. 3 2014), an mHealth software comprising a smartphone application (“app”) and clinician Dashboard (see Fig. 1)." |
| Bauer, 2018 – 636 [74] | Ginger.io | "The mobile health platform was furnished by Ginger.io and included a smartphone app (available for iPhone or Android devices) for patients and a web-based provider dashboard" |
| Patel, 2019 – 891 [75] | Microsoft Excel | “A user-friendly Maternity Dashboard was produced in Microsoft Excel 2016. The display of the dashboard was placed in the delivery suite for a monthly review by frontline staff.” |
| Pandya, 2018 – 870 [39] | Microsoft Excel | “We created the visual dashboard in MS Excel and placed it in a central location easily accessible by all members of the obstetric anaesthesia team.” |
| Lenglet, 2019 – 991 [76] | Microsoft Power BI | “Using the KoboCollect application programming interface, a set of clearly defined methods for allowing the communication of data among different applications and platforms, we connected data from the KoboToolbox server to Power BI (Microsoft Corp), a partially proprietary online platform for automated data analysis with an interactive visualization display (dashboard). The application programming interface was used to populate Power BI, and the dashboard was refreshed daily.” |
| Harzand, 2018 – 764 [77] | Moving Analytics | “The intervention was a 12-week home-based CR program delivered via a commercially available smartphone platform (Moving Analytics, Los Angeles, California) that included a patient-facing iOS and Android compatible smartphone app and an integrated hospital facing online dashboard for remote patient monitoring and care coordination by a trained coach.” |
| Kumar, 2018 – 853 [78] | LifeData System | “EP [early psychosis] clients and treatment providers used the LifeData system [18], a mobile technology suite comprising 2 parts: a secure Web-based provider dashboard and the smartphone app RealLife Exp. EP clients responded to individual survey sets, called “LifePaks,” via RealLife Exp and providers viewed these responses on the dashboard” |
| Hoogeveen, 2018 – 4720 [26] | Custom Build + Highcharts | "The GCP was hosted on Microsoft Azure (refer to Azure subscription agreement: https:// azure.microsoft.com/en-us/support/legal/subscriptionagreement/). For the GSD App development, the open source frameworks AngularJS, JavaScript jQuery, and Bootstrap were chosen. A non-commercial license from Highcharts (https://www.highcharts.com) was used to display the graphs in the GSD clinical dashboard." |
| Frymoyer, 2020 – 1088 [19] | Custom Build + InsightRx | "MIPD was operationalized using InsightRX, a commercially available, cloud-based precision dosing platform that functions as a CDS tool...The MIPD CDS tool was integrated into our EHR, which supported the functionality of the tool within the clinical workflow and reduced the burden of providers having to manually enter relevant clinical data from the EHR into the tool. To accomplish, we worked with the vendor to design and develop a custom web interfaceThe integration was accomplished in our EHR (Epic Systems, Verona, WI) via a “Med Management” hyperlink icon that displays within the “Web Resources” tab of a patient’s electronic chart (see Figure 3). Upon clicking the icon, the MIPD CDS tool (Figure 2) is opened in a web browser and a custom application program interface (API) developed by our clinical informatic specialists retrieves all the needed clinical data elements for MIPD from our EHR and compiles them in a JavaScript Object Notation (JSON) file format to be transmitted over a secure (Hypertext Transfer Protocol Secure) connection." |
| Shailam, 2018 – 624 [79] | Logi Info Business Analytics System | “Using a Logi Analytics Logi Info Business Analytics system, the collection of SQL databases managed are queried every 90 seconds to generate an intuitive dashboard view that highlights pediatric studies to be performed on a given day (Fig. 1). The dashboard, published as a web page, is viewed in the pediatric radiology reading room.” |
| Rezaei-Hachesu, 2018 – 666 [41] | Microsoft Power BI + Microsoft Visual Studio + Custom Build | “This module was constructed in Microsoft Visual Studio 2015 with the Windows Presentation Forms, Language Integrated Query (LINQ), and Model-View-Controller (MVC) in a three-tier architecture. Based on the selected architecture, the data access tier, business tier, and user interface (UI) tier were coded for each sprint. The data model was designed and mapped in SQL Server 2012…knowledge extraction algorithms were implemented using Microsoft BI management tools.” |
| Staples, 2020 – 1189 [80] | ORBIT+, Orbit Analytics | “We wanted to replicate the effect of the positive outcomes from the hematology ward feedback on a larger scale to the rest of the hospital. RBC unit ordering data are combined into an institution-wide reporting and analytics tool that allows visualization of product ordering data (ORBIT +Plus, Orbit Analytics).” |
| Kummer, 2019 – 1004 [28] | iNYP Clinical Information System | “In conjunction with New York-Presbyterian (CUIMC’s hospital) and the Department of Biomedical Informatics at CUIMC has a clinical data repository that dates back to 1988, and maintains a web-based, display-only clinical data review platform (i-NewYork Presbyterian [iNYP]; http://inyp.nyp.org/inyp) that is separate from the transactional EHR used by the hospital and the ancillary systems.28 This platform is accessible via single-factor authentication from within the hospital intranet and through dual-factor authentication from outside the hospital intranet. Via an interface, the iNYP Clinical Information System (iNYP) ingests data from the main hospital EHR and admission/discharge/ transfer system, as well as laboratory, radiology, ultrasound, and neurophysiology reporting systems (►Fig. 1). The iNYP platform also contains several aggregative “dashboard” pages that display tailored clinical data to particular clinical user groups. Given that many inpatient consultations at CUIMC are focused on stroke, a vascular neurology-oriented dashboard was conceived, developed, and implemented as a clinical informatics quality improvement project between August and December 2017” |
| Dagliati, 2018 – 642 [11] | Custom Build + Google Charts | “The datamining module components were developed using R and Matlab, and they communicate with the other components exchanging data in JavaScript Object Notation format. The technologies exploited to develop the graphical user interface (GUI) are based on JavaScript, Hypertext Markup Language, and Cascading Style Sheets. Communication occurs through asynchronous JavaScript and Extensible Markup Language requests. The technology used to create all the charts in the GUI was provided by Google Charts.” |
| Graber, 2019 – 1143 [81] | Microsoft Excel + Pyramid Analytics | “The Pyramid Analytics (Kirkland, WA) platform allows for exportation of data into Microsoft Excel and graphs into figures that could be downloaded by stewards for presentation or local manipulation.” |
| Smith, 2020 – 1185 [82] | Stata | “All statistical analysis and visual displays were performed and created using Stata statistical software (Version 15, StataCorp, College Station, TX).” |
| Jung, 2018 – 695 [83] | Decisio Health Inc | “An automated clinical surveillance visualization system (Decisio Health Inc, Houston, TX; www.decisiohealth. com) was implemented within the SICU at the University of Cincinnati Medical Center.” |
| Whidden, 2018 – 698 [61] | Microsoft Excel + Klipfolio | “The data manager compiled the Excel databases from different data clerks, generated queries to correct for data entry errors, and uploaded the data files to the cloud-based application (Klipfolio), upon which the data analysed for all CHWs in this study is based and from which monthly CHW Performance Dashboards were generated for CHWs in the intervention arm.” |

# Supplementary Table 6. All Reported Combinations of Dashboard Delivery Channels

| **Delivery channel combinations** | **Dashboards with reported combination of delivery channels to access dashboard** – n (% of 89 with available data) | **References** |
| --- | --- | --- |
| Website | 33 (37.1%) | [10,17,27,28,31,34,37,38,55–57,59,62,69,71,73,74,76–78,80,84–94] |
| Embedded in EHR | 13 (14.6%) | [2,5,18,20,21,44,45,47,54,60,95–97] |
| Site Intranet | 8 (9.0%) | [3,7,30,52,63,64,67,98] |
| Posted in setting | 7 (7.9%) | [13,42,46,58,75,82,83] |
| Other | 4 (4.5%) | [41,49,99,100] |
| Sent by email | 3 (3.4%) | [43,101,102] |
| Software app, Sent by email | 2 (2.2%) | [8,72] |
| Software app, Posted in setting | 2 (2.2%) | [61,103] |
| Website, Other | 2 (2.2%) | [26,104] |
| Website, Sent by email | 2 (2.2%) | [35,81] |
| Site Intranet, Other | 2 (2.2%) | [33,50] |
| Website, Software app | 2 (2.2%) | [53,105] |
| Embedded in EHR, Site Intranet | 1 (1.1%) | [36] |
| Embedded in EHR, Sent by email | 1 (1.1%) | [106] |
| Embedded in EHR, Posted in setting | 1 (1.1%) | [15] |
| Sent by email, Posted in setting | 1 (1.1%) | [66] |
| Website, Posted in setting | 1 (1.1%) | [79] |
| Embedded in EHR, Website | 1 (1.1%) | [19] |
| Software app, Sent by email, Other | 1 (1.1%) | [6] |
| Sent by email, Site Intranet | 1 (1.1%) | [12] |
| Sent by email, Site Intranet, Other | 1 (1.1%) | [32] |

# Supplementary Table 7. How End-Users were Involved in the Design, Implementation, or Evaluation of Healthcare Dashboards

| Study Author, Publication Year – study ID [Reference] | Details of End-User Involvement |
| --- | --- |
| Ahern, 2020 – 1098 [1] | "Following the development of the CQR summary document template, AH CGU staff developed a traffic light-style dashboard for each registry that allowed benchmarked data to be tracked and visualized over time." |
| Bae, 2020 – 999[2] | “A multidisciplinary task force of physicians, nurses, infection specialists, epidemiologists, and IT experts headed by the CIO was convened to manage and direct changes to health information systems for this effort.” |
| Barbeito, 2019 – 1020 [3] | "These dashboard prototypes were iteratively refined based on informal feedback provided by prospective users and made available to each anesthesiology attending provider through the Duke Anesthesiology Department’s intranet webpage." |
| Barnett, 2019 – 1043[4] | "Visual displays of the data were built by data analysts in partnership with the clinical owners and end-user clinicians. This partnership model is based on codesign principles.” |
| Bersani, 2020 – 1027[5] | "The Patient Safety Learning Laboratory (PSLL) focused on engaging patients, families, and professional care team members in identifying, assessing, and reducing threats to patient safety. With support from the Agency for Healthcare Research and Quality, the PSLL team of researchers, clinicians, health system engineers, technical staff, and stakeholders developed a suite of patient- and provider-facing tools to promote patient safety and patient-centered care. The Patient Safety Dashboard was one of the provider-facing tools developed." |
| Brink, 2020 – 1065[6] | "The prototype was tested in four pilot hospitals by HH testing teams (NSMs, IPC nurses and pharmacists) over a four-month period (February 2016 to May 2016). The pilot provided input to improve the software as well as important learning about the implementation processes that were incorporated in the launch and implementation. This phase was also used to test the summary dashboard for individual hospitals to use for feedback to staff as well as a full set of reports for regional manager and executive leadership review." |
| Burningham, 2020 – 3721[7] | "SQL Server Reporting Services was used in producing the user interface, performance measures, and required visuals and in adding interactive functionality to the dashboard. The team used an agile development approach when designing the user interface, making small iterative changes after obtaining feedback from key stakeholders.25 Initially, the interface was developed based on a set of core audit and feedback elements decided on by the EQUIPPED team, which is composed of pharmacists, epidemiologists, data scientists, and physicians with expertise in geriatrics and medication tolerability." |
| Cassim, 2020 – 1085[8] | “Focus group meetings were arranged with local area and business managers to define a test basket for the dashboard… A mapping table was developed to identify the LIS test sets and items to be reported. For each test, the TAT cut-off was also stipulated. The mapping table was used to guide the data extract.” |
| Chaparro, 2020 - 1028[9] | “In early 2018, we formed a multidisciplinary QI group to review and identify why we had increasing volumes of interruptive alerts. The group included attending physicians from several service areas, a resident physician, a nurse practitioner, and Epic analysts… To identify failings in the current alert ecosystem, the group constructed an Ishikawa diagram regarding all the potential causes excess alerts may be showing to providers. The group then categorized these causes into four main themes: lack of clinical relevance, poorly built alerts, lack of governance, and increasing patient volume/ acuity. Based upon these findings, a key driver diagram was developed to identify targets for interventions (►Fig. 3)… A dedicated interruptive alert team (IAT), consisting of one physician informaticist and two Epic analysts, was established to act as the primary conduit for all interruptive alert build and changes for the Nationwide Children’s Hospital system.” |
| Coventry, 2019 – 898[10] | “The critical innovation in IGR3 was the addition of an online dashboard that was accessible by participants and health advisors. The dashboard was co-designed with patient groups and front-line NHS Health Advisors, based on both Hitachi’s experience of delivering diabetes prevention and wellbeing programmes in Japan and SRFT experience of developing and delivering a telehealth diabetes prevention programme known as Diabetes Care Call.” |
| Dagliati, 2018 – 642[11] | “The GUI was designed to cover 2 different scenarios of use, the first aimed at supporting clinicians and health care practitioners in their day-to-day activities, and the second at facilitating periodic reviews of the ICSM Diabetology service data. For both scenarios, decision support is delivered by a dashboard that presents results through visual analytics solutions. The functionalities of these scenarios, which we refer to as the clinical decision support system (CDSS) scenario and the outcome assessment and research system (ORSS) scenario, are presented in the next 2 paragraphs. Design of the system followed a user-centered technique, involving users and stakeholders in an iterative approach.55,56” |
| Dixit, 2020 – 1187[12] | "Visualization development was driven by a user-centered design process that put the needs of end users at the forefront of design and development.5–7 This design process involved developing prototype visualizations, soliciting feedback from end users, and iteratively improving the visualizations...Our multidisciplinary visualization team used a 5-step process to create and iteratively refine visualization dashboards to support the initial launch and ongoing development of the expansive telehealth program." |
| Dolan, 2019 -792[13] | "As part of a hospital-wide effort to develop a more robust controlled substance diversion prevention program, we formed a core team of individuals that included anesthesiologists, informaticists, and pharmacists. Each of these departments was represented at both staff and director levels. Beginning in November 2017 the QI project team developed an interactive dashboard using the Tableau business intelligence tool." |
| Elm, 2019 - 909[14] | “This study focused on the distinct challenge of a lack of a widely accepted, user-friendly platform to report this type of data to clinicians in an easily understandable interface. CIS-PD was launched to assess the feasibility of use of the FWC app by PD patients over a 6-month period, to evaluate the utility of app-derived data to inform clinical decision making, and to iteratively develop and optimize a dashboard for data reporting with direct input from clinicians over the course of the study.” |
| Field, 2018 – 4496[15] | “The primary project team for each unit consisted of a Clinical Nurse Manager, Informatics Nurse and a Technical Project Manager. The design sessions involved an expanded team, including: Charge Nurses, Residents, Attending Physicians, Clinical Application Development Analysts, Informatics Provider, and Clinical Application Development Manager. One project concept meeting and three design sessions for each unit occurred over a 3-month time period. On an as needed basis, Care Coordination, Respiratory Therapists and Family Service Representatives were also included in the design sessions. The sessions were structured based on the template of the previously developed ICU electronic visibility boards. The combination of the Clinical Application Development Analysts with nursing and providers enabled new ideas to be brought to the design session to help the units generate more clinically relevant solutions. Specifically, the Clinical Application Development Analysts were able to hear about active problems from the nurse's clinical perspective and provide ideas for solutions, with this collaboration resulting in a more usable board.” |
| Findlay, 2020 – 1092[16] | "During all phases of this study, MDT members were consulted through a series of meetings with resulting feedback incorporated into the pilot model of care. Input was actively sought to co-design evidence-based nutrition care pathways and electronic clinical documentation templates, participation in relevant priority setting and education workshops, interviews and focus groups and ultimately to deliver and evaluate the model of care." |
| Fischer, 2020 – 1068[17] | A group of relevant stakeholders comprised the VHA dialysis dashboard committee that selected CPMs for the dashboard, and the technical subcommittee, which was tasked with identifying data in the EHR and utilizing it to power the dashboard data |
| Fletcher, 2018 – 2675[18] | "Programmers at the University of Washington, in partnership with hospitalist physicians, created the dashboard and tested it for stability and usability prior to the study." |
| Frymoyer, 2020 – 1088[19] | "To develop and implement an updated approach for vancomycin management at our hospital, we formed a multidisciplinary team of physicians (Neonatal and Infectious Disease specialists), clinical pharmacists, and clinical informatic specialists." |
| Fuller, 2020 – 1090[20] | "For this study, during the design and development phase, we conducted informal workflow observations on study units and interviews with stakeholders to identify end-user requirements for addressing these gaps by engaging patients and clinicians in discharge preparation while aligning with key organizational priorities: engaging patients to improve patient satisfaction, improving expected discharge date (EDD) documentation in the EHR, and reducing 30-day hospital readmissions"..."As in the concurrent PSLL study [27,29,36], we applied user-centered design principles to refine patient- and clinician-facing intervention components (Figure 1) to ensure that we addressed end-user needs." |
| Fuller, 2020 - 1044[21] | "The dashboard (Fig. 1) was developed using user-centered design principles to aggregate and display real-time clinical information for hospitalized patients. Clinical end-users, as well as nursing and medical leadership, were consulted during the iterative design and development of the display, logic, and workflow." |
| Giordanengo, 2019 – 897[22] | “We organized two facilitated workshops using a participatory design approach [29] involving four of the authors (AGi, AGr, EÅ, and AH), four clinicians (two nurses and two doctors who have worked with patients with diabetes), and two patients with diabetes… The facilitated workshops lasted 3 hours each, and participants were invited to use their own experiences to contribute to the workshops’ primary objectives. The majority group decision–making technique was employed during these sessions. In addition to the facilitated workshops, we organized a total of 11 sessions with open-ended discussion—three focused on mathematical models to use for medical and statistical calculations and involved two computer scientists; four focused on targeting the GUI usability, namely, the information to be displayed, which was attended by one computer scientist and one GUI expert; two focused on a first assessment regarding the medical relevance of the information displayed, which was joined by a computer scientist and a general practitioner; and two focused on the evaluation of the dashboard prototype against the requirements and involved four of the authors (AGi, AGr, EÅ, and AH)…. The prototyping phase consisted of implementing the dashboard to support the given scenarios by using computer-generated data that express the data requirements for the scenarios… The developed prototypes were assessed during the workshops and improved during each iteration of the design process.” |
| Grange, 2020 – 966[23] | “Enterprise IC needed visibility of events, actions, and changes happening across the enterprise to support decision making in the HICS. They provided metric requirements, and ITS created a real-time dashboard (►Fig. 2) of critical metrics including the number of tests by result and facility per day, laboratory turnaround time, current admitted patient counts, counts of personal protective equipment on hand by facility, and telehealth visits per day related to COVID-19.” |
| Hebert, 2018 – 630[24] | “The automated algorithm was initiated and developed using the CDC/NHSN VAE definitions released in January 2013. To translate the paper-based algorithm into a computer-based coded algorithm, an agile software design approach was used. Following this approach, over the course of approximately 6-9 months, there was an intensive, iterative process of information gathering, data field validation, and algorithm validation between the involved IPs (J.S. and J.F.) and IT data analyst (J.D.). Using a series of patients with known VAEs (by ongoing manual chart review surveillance), the algorithm was continuously tested until consistent agreement was reached. Dashboard development: Using the automated algorithm, a VAE report is generated on each ventilated patient by automatically extracting the required data from discrete fields within the EHR, classifying all MV patients using the CDC/NHSN algorithm parameters for VAE surveillance, and populating a simple dashboard used by the IPs to perform VAP surveillance.” |
| Hester, 2019 – 956[25] | "As a fifth QI intervention a pediatric hospitalist (bronchiolitis workgroup lead) and an IT dashboard developer partnered in modifying a vendors analytic dashboard33 for use in bronchiolitis. They determined target patient population based upon previous guidelines/studies,2,34 categorized clinicians (ED vs. observation/inpatient [hereafter referred to as inpatient]), categorized tests/treatments (e.g., medications considered antibiotics), determined display metrics, benchmarks,34 and verified data accuracy over 6 months. The hospitalist monitors the dashboard monthly during bronchiolitis season and works with the IT dashboard developer to resolve data accuracy or display issues." |
| Hoogeveen, 2018 – 4720 [26] | "The GCP was designed during monthly meetings with input from software developers, (parents of) GSD patients, researchers, and healthcare providers from June 2014 till present. The GCP was composed of two web applications; the GSD App for patients and their caregivers and the GSD clinical dashboard for healthcare providers." |
| Jameie, 2019 – 728[27] | “We composed a steering group that advised on design, development, evaluation, and implementation of the platform. Six rehabilitation mentors who were expert in cardiology, physiotherapy, nursing, psychology, and nutrition, two medical informatics specialists and one software engineer were the members of the steering group. We conducted an iterative, user-centered process to review the prototype and optimize the proposed layout… The process of creating and evaluating a telerehabilitation platform included four phases: (1) needs assessment, (2) design the prototype, (3) implement the cardiac telerehabilitation platform, (4) usability evaluation. 2.1. Need Assessment In this phase, we reviewed the scientific literature about cardiac rehabilitation to identify the core components of CR and IF-THEN rules [8-11]. Then we performed a comprehensive need identification by a semi-structured interview with MI survivors and rehabilitation mentors.” |
| Kummer, 2019 – 1004[28] | “One vascular neurologist completing fellowship training in clinical informatics gathered requirements for the dashboard from four attending stroke neurologists to finalize the dashboard's clinical content.” |
| Kunjan, 2019 – 4820[29] | "First, the specific information needs of potential dashboard users were evaluated through contextual inquiry/interviews...Role-specific contextual interviews were carried out at the clinic site. Contextual interview is a critical method in gathering design requirements. It involves probing users about their work while they are performing work-related tasks in their natural environment." |
| Laurent, 2020 – 1172[30] | “In a first step (“end user needs”), we met potential end users and conducted semi-directive interviews to define the end goal. We used the interview material to identify and synthesize issues, and thus identify indicators (variables, measurements, and filters). Each indicator was associated with one or more dashboards. In a second step (“prototyping”), we developed a number of potential solutions by applying good visualization practice relevant to the tool under development (including simple representations, consistent layouts, labels, and date formatting) [33–35] and presented them to end users for appraisal. In the third and last step (“deployment and preliminary evaluation”), the dashboards were implemented and made accessible to end users for everyday use.” |
| Mabirizi, 2018 – 712[31] | "From the start of the implementation, SIAPS identified pharmacists and pharmacy assistants who were interested in using computers and electronic tools and empowered them as early adopters and ambassadors of the tools. The tools were developed with substantial input from pharmacy staff, which created ownership and interest in the success of the tools. Further, tool development took into account existing processes and procedures of collecting and reporting pharmaceutical information." |
| Martinez, 2018 – 761[32] | “In September 2015, an internal workgroup consisting of hospital administrators, data engineers and data scientists was formed to organize and prioritize patient flow metrics in the JHH-CCC, and to build the e-Dashboard to facilitate monitoring and communication to diverse stakeholders… From September to December 2015, the workgroup conducted multiple semi-structured interviews with the hospital leadership and the service and unit managers (e-Dashboard users) to understand the barriers to monitoring operations over time and driving change… To fulfill the needs of the two user groups, we designed the e-Dashboard to show metrics by service, and then the users can drill down into specific units and time spans. From January to September 2016, our work team built the e-Dashboard.” |
| Minton, 2020 – 1165[33] | “We used the larger national data set and after an iterative process with a range of junior doctors to design a simple spreadsheet with 20 questions that could be completed on an ad hoc rolling basis to monitor trends over time and to allow for thematic feedback to teams we focused on the areas of recognition of the dying, communication, treatment escalation planning and the five priorities of care of a dying patient.” |
| Mooney, 2019 – 3016[34] | "We asked providers to comment on the dashboard display system and indicate features that might be useful and then incorporated that feedback in the display design." |
| Mulhall, 2020 – 1030[35] | "The reports were co-designed with physicians who would use the reports, in consultation with experts in adult learning, audit and feedback science, behavioral sciences, and stakeholders representing the long-term care sector." |
| Nelson, 2019 – 977[36] | “We convened a focus group of anesthesiologists, nurse anesthetists, and radiation oncology nurses to discuss the relevant elements of the anesthesia record… We then built a visual analytics tool to display this key data from multiple anesthesia encounters in a single screen. The resulting dashboard was created in accordance with the design heuristic principles of match between system and the real world and aesthetic and minimal design… Usability testing of the dashboard was conducted and compared with the standard EHR interface. The number of clicks and mouse scroll clicks to go through the multiple screens of a single anesthesia record on our existing EHR were counted by a nurse anesthetist and reviewed by a senior attending anesthesiologist (J.A.G.). The same process was repeated using the patient visual analytics dashboard. Click and scroll click counts for each method were then calculated using the number of patients on the actual schedule and the number of prior anesthetic records for each of these patients on a randomly selected clinical day. The number of mouse hovers required to review the dosage of one medication per prior anesthetic record was assessed for the dashboard view.” |
| Ospina-Pinillos, 2018 - 668[37] | “The research and development (R&D) cycle for the codesign and build of the MHeC included several iterative PD phases (Figure 1): PD workshops (phase 1); translation of knowledge and ideas generated during workshops (“knowledge translation”; KT) to produce mockups of webpages (either hand-drawn sketches or wireframes; phase 2); and rapid prototyping and one-on-one consultations with end users, including assessing the usability of the online alpha build of the MHeC (phase 3)… PD workshops were held in two stages with young people and youth health professionals attending separate workshops (stage 1) or a combined workshop (stage 2; Textbox 1). Following each workshop, the knowledge and ideas generated during the workshop were translated to produce mockups of webpages, either as hand-drawn sketches or wireframes… The mockups were then presented at the next workshop, enabling content and broad design ideas to be discussed, critically analyzed, and further developed.” |
| Palin, 2020 – 1115[38] | "An iterative design process was employed to codevelop useful analyses and visualisations with stakeholders, always updating and incorporating end-user feedback." |
| Pandya, 2018 – 870[39] | "We developed three cutoff criteria for each quality metric—a normal range, mild abnormal range, and abnormal range warranting immediate redressal. The quality metrics and cutoff criteria were chosen by a literature review, review of the departmental statistics for the past 5 years and consensus after discussion with members of the unit and obstetricians at the study institute." |
| Rea, 2020 – 3496[40] | "The development of the dashboard was a collaboration between the Business Intelligence department of SCW CSU, the local commissioners and local primary, community and secondary care clinicians who would subsequently use the dashboard." |
| Rezaei-Hachesu, 2018 – 666[41] | “This developmental study follows from previous descriptive studies conducted by our research team that determined the important axes of AMR surveillance systems. A knowledge, attitude, and practice (KAP) study [13,14] was used to propose, design, and describe the architecture of an AMR surveillance system based on business intelligence (BI) indicators. In the current study, an AMR surveillance system for use in NICUs was designed in accordance with the Scrum methodology proposed by Hicks and Foster… The results of the focus group discussion held in the previous KAP study are shown in Fig. 1. We determined seven major AMR surveillance topics in eight managerial axes, as follows: surveillance methods in AMR, priority specimens, priority samples, priority pathogens and microbial agents, test methods and reporting scheduling, and recommended datasets and reporting protocols/tools.” |
| Romero-Brufau, 2018 – 777[42] | "The project approach was to have a multidisciplinary core team that met two hours per week, and included industrial engineers at the postdoc and faculty level from the Center for the Science of Healthcare Delivery; an IT developer/liaison; and representatives from the clinical practice: radiology fellows, attending radiologists, radiology nursing personnel, radiology technologists, and administrators. This allowed for a truly multidisciplinary approach to decision making." |
| Ruff, 2018 – 626[43] | "The CRAR was designed with input from and for use by the dental clinical care team to increase patient receipt of evidence based care, reduce caries incidence, abate disease progression, and improve patient care experiences and health out comes through joint patient and provider engagement.. Stakeholders and content experts – including organizational leadership, clinicians, IS specialists, and QI experts – were engaged as cross-functional teams working in concert to achieve the project aims (Supporting Information Figure 1): (a) sponsors visibly supported the project conceptually and through resource allocation; (b) core team members engaged in project conceptualization, development, and implementation; and (c) pilot end users from affiliate practices tested implementation and provided feedback. These teams were purposely designed to enable an iterative development process with significant user input such that end use was central in all development efforts.” |
| Scheinfeld, 2020 – 1100 [44] | “In collaboration with the radiology IT division we created and implemented a 2-tiered real-time dashboard to facilitate operational workflow. This allowed us to track overall emergency department patient census, ordered but not yet performed imaging studies, and performed but unread imaging studies. The capability of clicking to obtain information on specific studies was also incorporated. We describe our experience of how this information has improved our workflow, staffing, and trainee education.” |
| Schleyer, 2019 – 896[45] | “During a two-day study period in March 2017, we observed seven physicians over 17 hours delivering emergency care at Eskenazi Health and Indiana University Health (IUH) Methodist Hospital, the two busiest emergency departments (>100,000 patient visits/year) in the city of Indianapolis. In a total of 70 patient encounters observed, CareWeb was accessed twice, despite its potential utility10. Based on interviews with physicians, we identified several barriers to HIE use, such as technical difficulties, time delays, uncertainty about the presence of information as well as its expected relevance and having to leave the EHR to access CareWeb… The Dashboard then communicates with our FHIR-on-INPC Server and searches for five data elements important to managing patients with the chief complaint of chest pain (last EKG, cardiology note, discharge summary, cardiac catheterization report, and echo, stress, and nuclear medicine tests). These data elements were identified by IUH Methodist ED physicians as most important for managing patients with the chief complaint of chest pain.” |
| Schmidt, 2019 – 733[46] | "The final phases of the initial stage focused on distilling the findings from all activities into a prototype system of a patient monitoring dashboard. Through incremental collaboration with clinicians, a functional prototype was implemented and evaluated in a pilot study involving 18 nurses and 50 patients [10]. The participating nurses were also asked to assess the system using the System Usability Scale [11], and results indicated that the overall design philosophy of the system made it easy to understand and use." |
| Sheen, 2020 – 951[47] | "In late 2016, a glycemic management team was formed, which integrated physicians from various medical service departments and information technology experts, in order to develop and implement a hospital-wide glycemic management program. We designed an electronic glucose dashboard that analyzed and monitored all hospitalized inpatients using POC glucose systems as well as plasma glucose data obtained from the hospital’s biochemistry laboratory." |
| Sinvani, 2020 – 3493[48] | “A representative from HM [hospital medicine] and geriatrics worked with data analytics (Krasnoff Quality Management Institute) on creating a data dashboard… Clinicians worked alongside analytics and IT teams to establish data validity... To ensure completeness, the EMR pull included the use of billing codes as first-level inclusion followed by a provider generated EMR label. A real-time dashboard including all variables of interest was created. The dashboard allowed for ongoing discussion during steering committee meetings as well as a way to share progress with leadership and frontline staff.” |
| Stachelek, 2020 – 1080[49] | “Five specific metrics of physician noncompliance with radiation TP workflow were identified as high priority by a multidisciplinary operations team consisting of leadership from therapy, physics, and dosimetry, in addition to the clinical director." |
| Taber, 2019 – 940[50] | “The current system was developed using an iterative process based on our previous research. Patient focus groups, surveys, and pilot studies were conducted and applied to develop a system that was patient centric, acceptable to patients, and could provide optimal comprehensive monitoring without being overly intrusive or difficult to use on a day-to-day basis... After preliminary focus group sessions, a prototype mHealth application was developed and demonstrated to 99 renal transplant recipients.. The mHealth app was refined based on this demonstration, a follow-up survey, and provider-based feedback. We also conducted a 60-patient interventional pilot study to assess the benefit of using mHealth to monitor home-based blood pressure and glucose values... Preliminary system validation to ensure that accurate data were captured and shared by the patient, the mHealth app, and the portal occurred prior to implementation of the system within the ongoing clinical trial… Investigators also changed medications in the EHR and sent notifications about hospitalizations, ER visits, and medication changes to ensure that all alerts were functioning as intended. Data queries from the EHR were also validated by investigators through detailed manual chart abstraction of dummy patients’ data. Calculations occurring within the system for means and threshold limits were validated manually prior to implementation of the production-level system. As issues and errors were found during the validation process, they were brought to the attention of the system developers.” |
| Trinh, 2019 – 942[51] | "Inpatient pharmacy management developed the dashboard by defining productive and essential nonproductive work using the integrated health record and manual time stamps." |
| Tyler, 2018 – 663[52] | “Our 40-person, multidisciplinary project team included hospitalists, ED and/or UC providers, bedside nurses, respiratory therapists, clinical application specialists, pharmacists, and process-improvement specialists… A multidisciplinary kickoff meeting was conducted to understand the problem and develop interventions (‍Fig 1). The project team reviewed data from previous seasons and compared institutional performances to national benchmarks.‍ 4–6,15 Next, team members worked in small groups to brainstorm problem statements, key drivers that were unique to each resource, project aims, and proposed interventions… The project team used a dynamic data dashboard for real-time data collection and analysis. The dashboard allowed all project team members, regardless of their QI training or background, to monitor general trends in use over time that were specific to their areas of interest.” |
| Van Citters, 2020 – 1164[53] | "We employed user-centred design [18] to develop a CF dash- board that could support patient-clinician partnerships and display patient’s concerns, treatments, and self-reported outcomes alongside clinical, experiential, and functional information. An interdisciplinary design team worked within a larger idealized design project, using established methods [10] . The team met weekly from July to October 2015 and included 21 participants (1 adult with CF, 4 parents of children with CF, 7 programme directors/physicians, 5 coordinators/nurses, 3 re- searchers, and 1 site-level registry coordinator) from 2 adult and 3 paediatric CF programmes. The team formalized project aims, identified functionality and data elements, and planned for rapid-cycle testing. Appendix A shows the dashboard design, build, and testing timeline.” |
| Wang, 2020 – 1180[54] | “The Division of Rheumatology created an Opioid Working Group to address its goals of optimizing treatment of pain while lowering the risk of harm. This Group was informed by a broader Opioid Task Force of the University of Pennsylvania Health System (UPHS). The Working Group consisted of a Rheumatology Physician Lead, the Rheumatology Division Administrator, the Musculoskeletal & Rheumatology Service Line Director of Quality, a Rheumatology nurse, a Pain Medicine physician, and a clinical pharmacist… UPHS created an Opioid Dashboard page that was integrated into the electric health record (EHR) system (Epic Systems Corporation, Verona, WI) which recorded the process measures above for each provider. This dashboard allowed collection and presentation of data from the institutional level, to the practice location, down to the individual provider level. Every provider was able to look up his or her own data and those of anyone else within UPHS.” |
| Webers, 2019 – 968[55] | "In 2014 and 2015, rheumatologists (experts in the field of SpA), nurses and two experienced patient research partners were consulted on the design and content of SpA-Net."..."After the initial development phase, SpA-Net was evaluated in a test environment during multiple rounds of internal and external testing in 2015 and 2016. These rounds were aimed at both improving different aspects of the system and bug-testing. Results from testing were reported monthly to the development team to ensure rapid cycles of improvement… Part of the implementation strategy was engaging those who have to record data.23 To motivate rheumatologists and stimulate dynamic refinement of SpA-Net, staff meetings were organised every 2 months to evaluate the usability of SpA-Net in practice, discuss bugs encountered, demonstrate updated system features and provide feedback to care providers on the use of SpA-Net. After every meeting, feedback from staff was communicated to the development team." |
| Williams, 2018 – 706 [56] | "The initial design of the dashboards user interface was created following an iterative process which was informed by short interviews with key stakeholders involving six GPs, seven clinical commissioning group (CCG) pharmacists and one member of our patient and public involvement group. Prototype dashboard designs were reviewed by the stakeholders during interview and feedback sought. This feedback was incorporated into the design for the next dashboard iteration." |
| Woo, 2019 – 884a[57] | "Early on in our process, we sought input from all stakeholders, including transfusion service staff and supervisors, laboratory IT programmers, and transfusion medicine physicians. Constructive suggestions from transfusion service staff, in particular, were critical in designing and implementing an easy to use dashboard that displayed accurate, real-time, and clinically relevant data, for efficiently managing blood product inventory and blood product cooler status. The dashboards were designed in collaboration with laboratory informatics and our transfusion service." |
| Woo, 2019 – 884b[57] | "Early on in our process, we sought input from all stakeholders, including transfusion service staff and supervisors, laboratory IT programmers, and transfusion medicine physicians. Constructive suggestions from transfusion service staff, in particular, were critical in designing and implementing an easy to use dashboard that displayed accurate, real-time, and clinically relevant data, for efficiently managing blood product inventory and blood product cooler status." |
| Yoo, 2018 – 868 [58] | "The multidisciplinary Happy Emergency Room Team included 6 physicians, 4 nurses, 1 administrator, 2 quality improvement team members, 2 consultants, and 2 designers. While hospital staff provided inputs, consultants and designers tried to see things from patients’ perspective, thus balancing the conclusion. Several rounds of discussions and debates took place before the first dashboard principle and design feature was created." |

# Supplementary Table 8. Reports of Formative Usability Testing

| Study Author, Publication Year – Study ID [Reference] | Usability Testing Details |
| --- | --- |
| Burningham, 2020 – 3721[7] | "Once an early-release version of the dashboard was developed, 2 VA EQUIPPED implementation sites were provided printed screenshots to obtain their feedback. The dashboard interface was then further refined based on the provided feedback." |
| Dixit, 2020 - 1187[12] | "Visualization development was driven by a user-centered design process that put the needs of end users at the forefront of design and development.^5–7^ This design process involved developing prototype visualizations, soliciting feedback from end users, and iteratively improving the visualizations...With the variety of stakeholder and end user needs, our visualization team divided into pairs consisting of a lead developer, driving dashboard development, and a support developer, handling data issues or providing additional context around each telehealth platform or program. These pairs would create prototype dashboards, starting with a basic layout of data feeds in a development environment then rapidly iterating with end users to ensure the dashboards met their needs. They would also share these iterations with the broader visualization team to solicit feedback on the design and highlight any data discrepancies between dashboards. After stakeholder and visualization team approval, dashboards would be checked by 1 member of the visualization team to ensure proper functionality and labeling before being uploaded to a production environment." |
| Dolan, 2019 - 792[13] | “As part of a hospital-wide effort to develop a more robust controlled substance diversion prevention program, we formed a core team of individuals that included anesthesiologists, informaticists, and pharmacists. Each of these departments was represented at both staff and director levels. The team’s aim was to develop a QI initiative focusing on reducing controlled substance documentation discrepancy in the OR. Beginning in November 2017 the QI project team developed an interactive dashboard using the Tableau business intelligence tool. We presented the prototype dashboard to our monthly departmental meeting and held several informal co-creation discussions in which the target audience of anesthesiologists provided feedback that helped improve our initial design.” |
| Elm, 2019 – 909[14] | “Feedback on ways to make the dashboard easier to interpret and support clinical decision making were obtained from five clinician focus group sessions and resulted in ten dashboard updates during the course of the study, with about 4–6 clinicians attending each focus group. This included the creation of separate displays for ePRO and sensor-derived data display, the expansion of the Y-axis in certain displays for easier data comprehension, the addition of markers for medication intake across ePROs and sensor-derived data displays, updates to descriptive text and the addition of “info” buttons describing each display” |
| Field, 2018 - 4496[15] | "System Validation testing was completed by the Clinical Application Development Analysts team. A final meeting occurred with the Clinical Nurse Manager, Informatics Nurse and the Clinical Application Development Analysts team for design validation prior to implementation. The project nurses, Clinical Nurse Manager and Informatics Nurse had the ability to test the functionality in a production domain for one week prior to implementation." |
| Fischer, 2020 - 1068[17] | “The resultant web-based dashboard design was iteratively reviewed by human factors experts who applied usability heuristics and recommended format changes [26]…The implementation phase of VHA dialysis dashboard included pilot testing, a coordinated series of national rollout calls, and user acceptance testing. First, several VA dialysis facilities volunteered for pilot testing which involved validating eligible patient identification and extracted laboratory and medication data displayed on the dashboard. This process was critical in improving and refining the use of electronically abstracted data (e.g., administrative codes, laboratory values, etc.) for CPM calculation and patient identification…The pilot testing also allowed users to provide feedback regarding the web application for data entry.” |
| Fletcher, 2018 - 2675[18] | "Programmers at the University of Washington, in partnership with hospitalist physicians, created the dashboard and tested it for stability and usability prior to the study" |
| Frymoyer, 2020 - 1088[19] | "The user-interface of the MIPD CDS tool, which was optimized based on user testing at Stanford LPCH and other medical institutions, is shown in Figure 2...Several clinical pharmacists and members of the multidisciplinary implementation team had previously participated in the vendor’s end-user beta testing of the MIPD CDS tool during development of the design, user interface, and functionality of the tool. Prior to clinical use, quality control of the EHR integration was performed in approximately 25 patients over a three month beta testing phase to confirm the accuracy and completeness of patient data transmission from the EHR to the MIPD CDS tool." |
| Fuller, 1090 – 2020 [20] | “Key refinements were identified through multiple iterations of the original checklist within our research team (in part based on our experience with a transitions study funded by the Patient-Centered Outcomes Research Institute), 2 sessions with our hospital’s patient and family advisory council, and a short pilot in which we administered a paper-based prototype to a convenience sample of 10 hospitalized patients and requested feedback…The intervention went live in December 2017, starting with a 1-month wash-in period in which we debugged various technical components.” |
| Gardner, 2019 – 976 [59] | “From the beginning of this project, ramifications to the end users and the PA-PSRS database were considered with each proposed change…The selection of new data fields was determined by whether their inclusion imposed a major burden on data collection or the data interface, met the goal of the project, and/or filled gaps in falls-related information (i.e., an expanded list of specific falls prevention strategies). Collecting utilization data created the biggest challenge for determining the scope of the unit-level portion of the project. Program testing was performed by Authority staff and several hospital PA-PSRS users to identify any potential problems with the system changes before implementation.” |
| Giordanengo, 2019 - 897[22] | “An iterative design process was used, and a workgroup selected relevant information to be displayed, modelled real-life situations using the dashboard with a simulation-type scenario approach, and then implemented multiple dashboard prototypes which were assessed by stakeholders during workshops. Feedback provided during workshops informed dashboard revision, until a final prototype was deemed suitable. Once the final prototype was completed, clinicians participated in usability testing, completing questionnaires based on the System Usability Scale and the Computer System Usability Questionnaire.” |
| Hester, 2019 - 956[25] | “Organizational leaders were granted dashboard access; feedback from early users was collected informally over 1 month and resulted in minor changes. Issues with accuracy of individual hospitalist data related to resident order entry led to limitation of individual log-ins to ED clinicians and organizational leaders.” |
| Hoogeveen, 2018 – 4720 [26] | "Features and functionality were reviewed and issues on usability were managed, processed, and documented with the use of a Jira issue tracker from Atlassian® by software developers, researchers, and healthcare providers." |
| Jameie, 2019 – 728 [27] | “The system usability scale (SUS) was used to rate the system in both alpha and beta testing. In alpha testing, the steering group (six rehab mentors; two medical informatics specialists; one software engineer) interacted w/ the system and completed the SUS questionnaire. In the beta test, three medical informatics specialists; three cardiologists; and fifteen post-MI patients provided feedback on the system.” |
| Kunjan,2019 – 4820 [29] | "Finally, a high fidelity prototype of the dashboard was assessed, tested with users and later redesigned iteratively, based on feedback in the design. About 1 hour was allocated for each individual session with the following break-down of time: 30 minutes for Contextual Interviewing, 20 minutes for the Card Sorting activity, and 10 minutes for the Prototype Dashboard evaluation....After a demonstration of the prototype dashboard, participants were questioned regarding the various interaction features including: preferred user interface elements (including input controls, navigational, and informational components); preferred visual design (typography, images, and colors); and preferred mode of delivery of the dashboard (static reports via email vs. interactive visualizations delivered from a web portal)." |
| Laurent, 2020 – 1172[30] | “Different visual representations of indicators and different layouts, formats, styles, etc. were created initially and presented to future end-users for feedback throughout development. The most liked versions of each were selected for inclusion in the final dashboard.” |
| Mooney, 2019 – 3016 [34] | "We asked providers to comment on the dashboard display system and indicate features that might be useful and then incorporated that feedback in the display design." |
| Nelson, 2019 - 977[36] | "Usability testing of the dashboard was conducted and compared with the standard EHR interface. The number of clicks and mouse scroll clicks to go through the multiple screens of a single anesthesia record on our existing EHR were counted by a nurse anesthetist and reviewed by a senior attending anesthesiologist (J.A.G.). The same process was repeated using the patient visual analytics dashboard. Click and scroll click counts for each method were then calculated using the number of patients on the actual schedule and the number of prior anesthetic records for each of these patients on a randomly selected clinical day. The number of mouse hovers required to review the dosage of one medication per prior anesthetic record was assessed for the dashboard view." |
| Ospina-Pinillos, 2018 - 668[37] | "Employing a think-aloud protocol [72], participants were observed as they navigated the MHeC and responded to questions posed by the researcher about the main components of the MHeC; responses were recorded by the observer. The initial effectiveness of the system was then assessed by asking participants to complete 3 usability tasks: (1) create an account and login; (2) find the “need help now” button; and (3) book an appointment. Task completion time was recorded to assess the efficiency of the system. No instructions or clues were provided, and comments in relation to navigation were recorded." |
| Paulson, 2020 – 1059 [60] | “The remote VQT RN was included in simulations and debriefs, giving the teams an opportunity to practice the workflow together and exchange feedback in a safe and supportive environment. Conducting workflow practice runs two weeks prior to project launch—an integral training component— allowed the local team to test the workflow in advance of going live.” |
| Schleyer, 2019 – 896 [45] | “During the development of the Dashboard, we compared the traditional method of retrieving information from the INPC through CareWeb to doing so using the Dashboard. The Dashboard reduced the number of clicks from 50 to 6, and search time from 3 minutes to 10 seconds, in a laboratory experiment.” |
| Schmidt, 2019 – 733 [46] | "The final phases of the initial stage focused on distilling the findings from all activities into a prototype system of a patient monitoring dashboard. Through incremental collaboration with clinicians, a functional prototype was implemented and evaluated in a pilot study involving 18 nurses and 50 patients [10]. The participating nurses were also asked to assess the system using the System Usability Scale [11], and results indicated that the overall design philosophy of the system made it easy to understand and use." |
| Taber, 2019 – 940 [50] | “A preliminary system validation was completed that involved the investigators (clinical pharmacists / potential end users) testing the dashboard and mhealth application to identify and rectify any issues with usability or data accuracy. Additionally, a prototype mhealth app was developed and demonstrated to 99 renal transplant recipients for feedback and revisions, and a 60-patient interventional pilot study was conducted to assess the benefit of using mhealth to monitor bp and glucose.” |
| Webers, 2019 – 968 [55] | "After the initial development phase, SpA-Net was evaluated in a test environment during multiple rounds of internal and external testing in 2015 and 2016. These rounds were aimed at both improving different aspects of the system and bug-testing. Results from testing were reported monthly to the development team to ensure rapid cycles of improvement." |
| Whidden, 2018 – 698 [61] | “All eight supervisors and 150 CHWs participated in the current study: 148 CHWs were study participants providing written informed consent, while two CHWs pretested the Dashboard.” |
| Williams, 2018 – 706 [56] | “The initial design of the dashboards user interface was created following an iterative process which was informed by short interviews with key stakeholders involving six GPs, seven clinical commissioning group (CCG) pharmacists and one member of our patient and public involvement group. Prototype dashboard designs were reviewed by the stakeholders during interview and feedback sought. This feedback was incorporated into the design for the next dashboard iteration.” |

# Supplementary Table 9. Dashboard Metrics Informed by Payor, Government, or Professional Guideline Standards

| **Dashboard Purpose*** | **Dashboards with metrics informed by payor, government or professional guidelines (total n=43) – n (% of 43)** | **[References]** |
| --- | --- | --- |
| Performance monitoring (n=20) | 13 (30.2%) | [6–8,13,17,33,35,38,68,72,76,86,102] |
| Care coordination, Direct patient care (n=16) | 4 (9.3%) | [27,34,55,77] |
| Population management, Performance monitoring (n=9) | 4 (9.3%) | [4,16,54,97] |
| Population management, Direct patient care (n=14) | 4 (9.3%) | [24,41,104,107] |
| Utilization tracking, Performance monitoring (n=12) | 4 (9.3%) | [25,52,67,81] |
| Resource management, Utilization tracking, Performance monitoring (n=5) | 3 (7.0%) | [30,75,80] |
| Population management (n=5) | 3 (7.0%) | [89,98,108] |
| Population management, Care coordination (n=3) | 1 (2.3%) | [109] |
| Resource management, Utilization tracking (n=5) | 1 (2.3%) | [32] |
| Performance monitoring, support training and education (n=2) | 1 (2.3%) | [64] |
| Direct patient care (n=3) | 1 (2.3%) | [83] |
| Facilitate and support use of clinical quality registries (n=1) [1] | 1 (2.3%) | [1] |
| Population management, Utilization tracking, Performance monitoring | 1 (2.3%) | [43] |
| Population management, Direct patient care, Performance monitoring | 1 (2.3%) | [40] |
| Population management, Financial tracking, Utilization tracking | 1 (2.3%) | [110] |

| Study Author, Publication Year – Study ID | Dashboard Intended Purpose | Details on How Payor, Government, or Professional Guideline Standards Impacted Dashboard Metrics |
| --- | --- | --- |
| Ahern, 2020 – 1098 [1] | Facilitate and support use of clinical quality registries | “The National Safety and Quality Health Service Standards (NSQHS)2 require that health services have systems to monitor variation in practice against expected standards, and identify unwarranted clinical variation to inform clinical practice improvements… The ACSQHC has provided guidance on CQR development in Australia over the past decade through the publication of key documents, including the Operating Principles and Technical Standards for Clinical Quality Registries (2008), the Framework for Australian Clinical Quality Registries (2014) and the Prioritised list of Clinical Domains for Clinical Quality Registry development (2016)… In line with these national and state-based clinical governance expectations, health service managers are increasingly seeking clinical registry information… The objectives of this initiative were to: (1) identify clinician and unit participation in clinical registries at an Australian quaternary health service; (2) investigate opportunities to engage health service clinicians to share clinical registry information within the organisation; and (3) integrate CQR reporting within the health service clinical governance framework in order to maximise the value of CQR information for health service quality improvement… In particular, participation in clinical registries supports the National Accreditation Standard’s requirement for health services to monitor variation, provide feedback to clinicians and use this information to improve safety and quality.” |
| Anderson, 2019 – 1052 [104] | Population management + direct patient care | “Daily spontaneous awakening trials (SATs) and spontaneous breathing trials (SBTs), as well as sedation minimization protocols, reduce the duration of mechanical ventilation, tracheostomy rates, ICU and hospital length of stay (LOS), and mortality. Consequently, sedation minimization and ventilator liberation protocols are key components of the Society of Critical Care Medicine’s Assess, Prevent, and Manage Pain, Both SAT and SBT, Choice of analgesia and sedation, Delirium: Assess, Prevent, and Manage, Early mobility and Exercise, and Family engagement and empowerment (ABCDEF) bundle and ICU Liberation initiatives, and the American Thoracic Society/ American College of Chest Physicians Clinical Practice Guidelines. Despite their evidence base and inclusion in professional society guidelines, implementation is inconsistent, with some studies revealing only 40–44% compliance performing daily SATs. Audits at our institution similarly revealed that SBT assessments were inconsistent across ICUs, knowledge of our institution’s SBT eligibility criteria was incomplete, extubation delays were frequently related to over-sedation despite sedation minimization strategies, and that manual review of these processes limited real-time feedback… We designed a novel software platform, the Awakening and Breathing Coordination (ABC) Application, which continuously screens patients for opportunities to minimize sedation and speed ventilator liberation, and is coupled with a dashboard and text-message alerts to inform bedside providers when an opportunity is identified.” |
| Barnett, 2019 – 1043 [4] | Population management + performance monitoring | “The Australian Commission for Safety and Quality in Health Care has created the National Safety and Quality Health Service standards that all hospitals must address in order to remain accredited. These standards are endorsed by the Australian Health Minister’s Advisory Council and include safety and quality indicators such as pressure injuries, falls and cardiac arrests. Accreditation qualifies the hospital to receive funding. It is performed on a 2-yearly cycle, with accreditors examining performance across the 10 standards and assessing adequacy… A current state analysis was completed for each of the 10 national standards. This included compliance reporting requirements for the national safety standards, mandatory reporting requirements at both state and national levels, evidence-based practice (including literature review and published clinical guidelines), cataloguing individual ieMR data elements and linked systems, current reporting and data sources within the organisation and documentation and workflow practices associated with reporting requirements…Ten national standard dashboards were developed with data displays designed to be accurate, relevant, accessible and easily consumed by the end-user for application to clinical practice… The hospital used these products in parallel with traditional Australian Council of Healthcare Standards preparations for the accreditation visit in 2017. To our knowledge, this was the first time streaming clinical analytics for the 10 safety and quality standards have been used in an Australian health facility and contributed to the successful reaccreditation of the hospital.” |
| Brink, 2020 – 1065 [6] | Performance monitoring | “The collated results of the initial World Health Organization (WHO) Hand Hygiene Self-Assessment Framework (HHSAF) measurement tool acted collectively as an institutional diagnostic tool that served as baseline compliance to the indicators… The HH intervention was designed to improve HH compliance through change in practices by targeting of behavioural determinants contained within the WHO HHSAF. Such intrinsic determinants include knowledge (training and education), awareness (evaluation and feedback), and self-efficacy (use of role models such as unit managers to demonstrate proper HH behaviour).” |
| Burningham, 2020 – 3721 [7] | Performance monitoring | “The EQUIPPED dashboard ELT process was adapted from an existing dashboard system's backend architecture: the VA Geriatric Scholars program's PIM [potentially inappropriate medications] dashboard. Both EQUIPPED and the Geriatric Scholars program are informed by the American Geriatrics Society (AGS) Beers Criteria for medications that should be avoided in patients 65 years or older to identify PIMs. EQUIPPED uses the AGS Beers Criteria category 1 PIMs for all older adults to serve as a marker of prescribing quality to evaluate the effect of the program on prescribing behavior….Alternative therapeutic options for identified PIMs supported by the National Committee for Quality Assurance and the Pharmacy Quality Alliance have also been added to the medication dimension table.” |
| Cassim, 2020 – 1085 [8] | Performance monitoring | “At a national level, the corporate data warehouse (CDW) of the NHLS collated global TAT data from over 266 testing laboratories based on predetermined, annual performance plan cut-offs. National TAT cut-offs are set by expert committees of different pathology disciplines with final confirmation from senior management before implementation. These cut-offs are set with provisions for all levels of service laboratory: from low-volume laboratories with limited test repertoires to high-volume testing laboratories with extensive test offerings, including specialised testing, such as viral load testing… Data is aggregated and compared to the national cut-off for each test in the dashboard presented here. However, individual laboratories have established locally-relevant TAT cut-offs for emergency and routine contexts depending on the level of care (primary versus tertiary).” |
| Cassim, 2020 – 1086 [72] | Performance monitoring | “Turn-around time (TAT) is an important performance indicator of laboratory efficiency to deliver patient results.1 In the South African National Health Laboratory Services, ad hoc mean TAT reports were previously produced for laboratory managers. These TAT reports assessed performance based on the National Health Laboratory Service global annual performance plan (APP) TAT cutoffs specific for individual tests…Initially, the three measures described above were reported in Microsoft Excel (Redmond, Washington, United States) worksheet format from August 2016 to June 2017.4 Thereafter, from July 2017, an interactive dashboard was developed that reported TAT data for a basket of tests using the Microstrategy Desktop (Tysons, Virginia, United States) analytics tool…The aim of this study was to report on the impact of an interactive dashboard that provides weekly information about TAT and enables laboratory and senior managers to monitor TAT and identify problematic areas for corrective action.” |
| Dolan 2019 – 792 [13] | Performance monitoring | “The Controlled Substances Act (CSA) requires accurate accounting of controlled substance transactions by inpatient pharmacies, to assist in preventing and detecting controlled substance diversion. In 2015 Massachusetts General Hospital was required to pay $2.3 million in a settlement to the federal government and to develop a corrective action plan for failing to comply with the Act. In addition to prosecution under the CSA, hospitals may be prosecuted for fraudulent billing by regulatory federal and state entities if they appear to be over-accounting for medications used. The American Society of Health-System Pharmacists has developed guidelines for preventing diversion of controlled substances. Each health care organization is encouraged to develop a process that complies with state and federal laws and regulations, but despite this, a 15% discrepancy rate between dispensed and administered controlled substances is reported in the literature… The team’s aim was to develop a QI initiative focusing on reducing controlled substance documentation discrepancy in the OR. The initial goal was to reduce the controlled substance documentation discrepancy rate by 20% within 2 years and was inspired by previously published projects at Dupont Nemours Children’s Hospital (using a dashboard to track controlled substance documentation discrepancies)13 and Jefferson University (using a software program to reconcile the AMDS to the anesthesia EMR).” |
| Durojaiye, 2018 – 686 [64] | Performance monitoring + support training and education | “On the basis of the xAPI framework and using open-source software to extend their existing infrastructure, the authors developed a Web-based dashboard that provides residents with a more holistic view of their educational experience. The dashboard was designed around the ACGME radiology milestones and provides real-time feedback to residents using various assessment metrics derived from multiple data sources.” |
| Ebbens, 2019 – 910 [108] | Population management | “Medication reconciliation in transitions of care is known to prevent medication errors. The Dutch guideline ‘Medication accuracy at transition in care’ states that an accurate medication overview is required at each transition point of care… Medication reconciliation was performed by a medical attendant who is familiar with the medication of these nephrology patients and received specific training to use the tool ‘Medical Dashboard’ to perform medication reconciliation… Medication discrepancies were defined as any difference in medications, dose, frequency or route between the medication in the hospital electronic patient record and the result of the medication reconciliation recorded in the ‘Medical Dashboard’… All identified medication discrepancies were considered unintended, because medication reconciliation took place before the medical specialist could prescribe any new medication that could be considered as an intended discrepancy. For this reason, the medication discrepancies were defined as medication transfer errors (MTE). The MTE were classified into three categories: omission (observed to be in use but not recorded in the electronic patient record); commission (not observed to be in use, but documented in the electronic patient record) and change in dose, frequency, or route. Potential harm of the MTE was assessed using the National Coordinating Council on Medical Reporting and Prevention (NCC-MERP) classification system.” |
| Findlay, 2020 -1092 [16] | Population management + performance monitoring | "The HNC [head and neck cancer] EBGs [evidence-based guidelines] were developed according to recognised evidence synthesis methods and the Nutrition Care Model, comprised of three domains: Appropriate Access to Care (Nutrition Screening, Nutrition Assessment); Quality Nutrition Care (Goals, Prescription, Implementation); and Nutrition Evaluation and Monitoring (Measure and Evaluate Outcomes)… The Nutrition Care Dashboard served to highlight nutrition care processes and clinical outcomes and was integrated into the existing HNC Radiation Oncology list for discussion at the weekly MDT meetings. The Nutrition Care Dashboard was nominated by the MDT as a preferred intervention and was phased into meetings over several weeks through an iterative co-design process to incorporate feedback from members. A key design element included a traffic light color-coded flagging system to highlight the change from baseline in both degree of critical weight loss (<5%, ≥5 to <10% and ≥10%) and nutritional status as defined by the Scored Patient-Generated Subjective Global Assessment (Scored  PG-SGA) denoting those patients who were well nourished (A), moderately malnourished (B) or severely malnourished (C).” |
| Fischer, 2020 – 1068 [17] | Performance monitoring | “Quality measurement for chronic dialysis care is well established in the U.S. Beginning in the 1990’s, the Centers for Medicare and Medicaid Services (CMS) required ESRD Networks to monitor and improve the quality of dialysis care. Over the past decade, CMS has iteratively developed and refined a variety of clinical performance measures (CPMs) for ESRD for public reporting (e.g., Dialysis Facility Compare) and value-based purchasing (e.g., Quality Incentive Program) using clinical practice guidelines as an initial framework, but extending beyond consensus guidelines… Selecting ESRD CPMs for inclusion in the VHA dialysis dashboard consisted of an 8-month-long transparent, stakeholder-driven process. The first several meetings focused on establishing the principles to guide CPM selection… The subcommittee agreed to adopt CPMs from those already established and promulgated as mature measures by leading organizations instead of developing and vetting measures de-novo because these measures had already gained acceptance, would afford benchmarking, and lastly because of internal resource constraints. An environmental scan revealed five key sources for the potential ESRD CPMs: CMS, American Medical Association Physician Consortium for Performance Improvement (AMA-PCPI), Kidney Care Quality Alliance (KCQA) and organizations that review, endorse, and adopt measures (National Quality Forum (NQF) and Ambulatory Care Quality Alliance (AQA). A total of 78 measures from these organizations were identified and they spanned broad ESRD clinical domains such as mortality, hospitalization, dialysis adequacy, vascular access, anemia, bone and mineral metabolism, infection, immunization, transplant, and end-of-life planning (Supplementary Table 1).” |
| Graber, 2019 – 1143 [81] | Utilization tracking + performance monitoring | “Antimicrobial stewards have long lacked the ability to compare their antibiotic usage to either national norms or to comparable facilities. In this regard, the development of standardized antimicrobial administration ratios (SAARs) within the antimicrobial use (AU) option of the National Health Safety Network (NHSN) by the Centers for Disease Control and Prevention (CDC) has been a major advance. These reports provide facility-level measures of days of therapy per 1000 patient-days present (DOT/1000 DP) and utilize indirect standardization techniques to represent antimicrobial use data as observed to expected ratios. However, the NHSN reports do not provide bases on which an institution may compare its antimicrobial use to similar facilities nor demarcate antimicrobial use according to specific diagnoses or across the temporal course of therapy from initiation of empiric therapy through deescalation and subsequent discharge…To address this information gap, we extended previous projects that extracted inpatient antimicrobial use data from the Veterans Affairs’ (VA’s) Corporate Data Warehouse to develop a suite of interactive graphic tools that provide stewards with in-depth facility-level reports of antibiotic use. Antimicrobial use at the dashboard user’s (eg, steward’s) facility can be compared to all VA facilities or user-selected facilities of similar complexity levels, with plots of the system-wide variability of antimicrobial use… The first iteration of the antimicrobial graphic tools consisted of a web-based dashboard with 3 interactive modules that showed overall trends in antimicrobial DOT/1000 DP comparison of a single facility’s SAARs to other facilities and proportion of patients receiving specific antibiotics at each of the CCC intervals for PUS diagnoses. Stewards had the ability to track their facility’s antimicrobial use (overall, by class of drug, by SAAR category, by individual agent) according to month, quarter, or year stratified by ward type (medical/surgical ward vs intensive care unit). The second module showed the facility’s SAARs on a bar graph compared to other VA facilities that were sharing NHSN AU data, stratified by VA facility complexity.” |
| Hagaman, 2018 – 794 [68] | Performance monitoring | “We analyzed the implementation of a preoperative documentation standardization intervention in Vanderbilt’s Preoperative Evaluation Clinic (VPEC) and its impact outside VPEC. A phased intervention consisted of clinician education with monthly feedback, followed by the development of a compliance dashboard and inclusion in Ongoing Professional Performance Evaluation system by VPEC…OPPE dashboard used to track clinician adherence to the preoperative documentation standard. Dashboard reports case counts by providers and ASA, use of standardized HPI in documentation, and HPI on failed cases. Access given to VPEC clinicians during phase 2 of intervention. OPPE, Ongoing Professional Performance Evaluation; ASA, American Society of Anesthesiologists physical status classification system; HPI, history of present illness.” |
| Harzand, 2018 – 764 [77] | Care coordination + direct patient care | “The intervention was a 12-week home-based CR program delivered via a commercially available smartphone platform (Moving Analytics, Los Angeles, California) that included a patient-facing iOS and Android compatible smartphone app and an integrated hospital facing online dashboard for remote patient monitoring and care coordination by a trained coach. The coach was a cardiology physician assistant. The platform (Figure 1) delivered an exercise-based CR program using clinical protocols based on MULTIFIT, a case management system for secondary prevention in patients with CAD developed by Stanford University investigators. MULTIFIT has been previously shown to significantly improve low-density lipoprotein-cholesterol levels, functional capacity, and rates of smoking cessation over usual care.” |
| Hebert, 2018 – 630 [24] | Population management + direct patient care | “Prior to January 2013, the Center for Disease Control and Prevention/National Healthcare Safety Network (CDC/NHSN) surveillance definition for VAP included subjective interpretations of radiographic changes on chest films and the presence of signs and symptoms of pneumonia. In 2013, a new definition, termed ventilator associated event (VAE), was released by the CDC/NHSN which now requires only objective data (Fig 1). It included 4 tiers for VAE: ventilator-associated condition (VAC), infection-related ventilator associated complication (IVAC), possible VAP, and probable VAP. The latter 2 categories are now combined into possible ventilator associated pneumonia (PVAP) per the NHSN device-associated module from January 2015, last modified in January 2017…In this article we report on the implementation of an automated surveillance process for VAEs at a large, tertiary care, academic medical center using the CDC/NHSN VAE definitions in place as of 2014.” |
| Hertzke, 2018 – 633 [86] | Performance monitoring | “Using patients cared for by hospitalists from July 2010 through June 2014, we linked billing data across each hospitalization to assign “ownership” of patient care based on the type, timing, and number of charges associated with each hospitalization (referred to as “provider day weighted”). These metrics were presented to providers via a dashboard that was updated quarterly with their performance (relative to their peers)…Specific metrics included American College of Chest Physicians (ACCP)–compliant venous thromboembolism (VTE) prophylaxis,7 observed-to-expected length of stay (LOS) ratio, percentage of discharges per day, discharges before 3 pm, depth of coding, patient satisfaction, readmissions, communication with the primary care provider, and time to signature for discharge summaries (Table 1). Appropriate prophylaxis for VTE was calculated by using an algorithm embedded within the computerized provider order entry system, which assessed the prescription of ACCP-compliant VTE prophylaxis within 24 hours following admission. This included a risk assessment, and credit was given for no prophylaxis and/or mechanical and/or pharmacologic prophylaxis per the ACCP guidelines… Depth of coding was defined as the number of coded diagnoses submitted to the Maryland Health Services Cost Review Commission for determining payment and was viewed as an indicator of the thoroughness of provider documentation…. Readmission rates were defined as same-hospital readmissions divided by the total number of patients discharged by a given provider, with exclusions based on the Centers for Medicare and Medicaid Services hospital-wide, all-cause readmission measure.” |
| Hester, 2019 – 956 [25] | Utilization tracking + performance monitoring | “First, we joined a national bronchiolitis QI collaborative, and second gathered a CM bronchiolitis workgroup….Fourth, (February 2016) we published a local modification of the AAP 2014 bronchiolitis guideline and a companion order-set to our intranet and EHR (Cerner). Following local QI interventions there were anecdotal improvements but data delays emerged as a key barrier to success; data requested in March 2016 was delivered in August 2016. As data turnaround was crucial for targeted interventions we sought an alternate method to obtain data. Workgroup members had used operational dashboards (implemented 2011) and felt a clinical dashboard might improve bronchiolitis data procurement. As a fifth QI intervention a pediatric hospitalist (bronchiolitis workgroup lead) and an IT dashboard developer partnered in modifying a vendor’s analytic dashboard for use in bronchiolitis. They determined target patient population based upon previous guidelines/studies, categorized clinicians (ED vs. observation/inpatient [hereafter referred to as inpatient]), categorized tests/treatments (e.g., medications considered “antibiotics”), determined display metrics, benchmarks, and verified data accuracy over ~6 months.” |
| Huber, 2018 – 665 [67] | Utilization tracking + performance monitoring | “In response to mandates included in the Protecting Access to Medicare Act of 2014 (PAMA 2014), clinical decision support (CDS) tools for imaging orders are being widely implemented by health systems in the USA. These tools use the American College of Radiology appropriateness criteria (ACR AC or appropriateness criteria), which are consensus guidelines rating a variety of possible imaging studies for a given clinical indication. These guidelines are derived by panels of radiologists and other medical specialists. Studies are rated 1–3 for “usually not appropriate”, 4-6 for “may be appropriate”, and 7–9 for “usually appropriate”… Fig. 1 Dashboard layout in Tableau. The pane at the top right displays summary data describing the total number of exams, appropriateness score, and a timeline displaying study volume by score over time. In the middle of the dashboard, the data are displayed in terms of ordering department. The total number of studies falling into each appropriateness category is displayed as well as the percentage of the total. Finally, the bottom two panes provide information regarding specific types of exams. On the left is a breakdown of the most common exams with their appropriateness scores, so the user can search for high-yield issues that affect frequent orders. The total number of studies of each appropriateness category is displayed as well. On the right is a cloud map of studies of which more than 25% were scored as low utility (appropriateness score 1–3, per ACR appropriateness criteria). This can help identify problematic exams that are receiving excessively high frequency of low scores which could be due to technical reasons or which may warrant education in clinical areas.” |
| Jameie, 2019 – 728 [27] | Care coordination + direct patient care | “Based on standard of British Association for Cardiovascular Prevention and Rehabilitation (BACPR), the core component of cardiac rehabilitation includes, health behavioral change, long term strategies, lifestyle risk factor management (including physical activity, healthy eating, Tobacco cessation and relapse prevention), nutritional counseling, psychosocial health, medical risk management, education, audit and evaluation. This framework was used as the basis of our study… Overall, the cardiac telerehabilitation platform consisted of three components: (1) an Android-based smartphone application for the patient, (2) a rule-based expert system that collects and processes the patient data entries, and (3) a dashboard for rehabilitation mentors… For pre and post-exercise biometric measurement, the different rules were defined based on scientific literature. These rules were verified by clinicians. We used the Karvonen Formula to Calculate the Target Heart Rate. The adjusted ideal body weight formula was used in body weight rule set to reduce patient body weight as much as possible. We used two set of rules for interpretation and calculating the score of patient answers to both DASS questionnaire and PSQI questionnaire.” |
| Jung, 2018 – 695 [83] | Direct patient care | “The Surviving Sepsis Campaign was launched in 2004 and provided guidelines for the diagnosis and treatment of severe sepsis and septic shock.4 The Third International Consensus Definitions for Sepsis and Septic Shock defines sepsis as a serious blood infection and associated acute organ dysfunction as outlined by the Sequential Organ Failure Assessment (SOFA) score: vasopressors, mechanical ventilation, elevated creatinine, elevated total bilirubin, thrombocytopenia, and elevated lactate.5-7 The most recent guidelines were published in 2016 and emphasize early fluid resuscitation, source control, and administration of intravenous antibiotics… In July 2017, an automated sepsis screen score (SSS) was activated within the clinical surveillance system and made viewable on the bedside display system. The SSS is a numerical score based on prior validated definitions and is composed of heart rate, body temperature, respiratory rate, and white blood cell count.18 Each value is assigned a value, which is then totaled to provide the SSS (Table 1)…The visible SSS was implemented into the bedside clinical surveillance system in July 2017. When comparing patients admitted to SICU beds with a bedside display only installed, the inclusion of the visible SSS was associated with a significant reduction in the time to antibiotic administration and decreased ICU and overall hospital LOS.” |
| Khanna, 2019 – 1029 [110] | Population management + financial tracking + utilization tracking | “A total of 48 practices with 109 practice sites participate in the Garden Practice Transformation Network in Maryland (GPTN-Maryland) to work together toward practice transformation and readiness for the Quality Payment Program implemented by the Centers for Medicare & Medicaid Services. Practice-specific data are collected in GPTN-Maryland by practices themselves and by practice transformation coaches, and are provided by the Centers for Medicare & Medicaid Services. These data are overwhelming to practices when presented piecemeal or together, a barrier to practices taking action to ensure progress on the transformation spectrum. The GPTN-Maryland team therefore created a practice transformation analytics dashboard as a tool to present data that are actionable in care redesign.” |
| Laurent, 2020 – 1172 [30] | Resource management, utilization tracking + performance monitoring | “Dashboards were implemented and made accessible for everyday use via the medical center’s network. After a period of use, end user feedback on the dashboard platform was collected as a system usability score (range 0 to 100). Seventeen themes (corresponding to 29 questions and 42 indicators) were identified. After prioritization and feasibility assessment, 10 dashboards were ultimately implemented and deployed. The dashboards variously addressed the unit’s overall activity, compliance with guidelines on intraoperative hemodynamics, ventilation and monitoring, and documentation of the anesthesia procedure…Specifically, in reference to current guidelines and recent literature, key indicators identified by end-users included the incidence of hypotension [40], ventilatory settings and related monitored variables (particularly driving pressure) [41], fluid administration [42] and blood transfusion [43]” |
| Lenglet, 2019 – 991 [76] | Performance monitoring | “IMPORTANCE Hand hygiene adherence monitoring and feedback can reduce health care–acquired infections in hospitals. Few low-cost hand hygiene adherence monitoring tools exist in low-resource settings.  OBJECTIVE To pilot an open-source application for mobile devices and an interactive analytical dashboard for the collection and visualization of health care workers’ hand hygiene adherence data.  DESIGN, SETTING, AND PARTICIPANTS This prospective multicenter quality improvement study evaluated preintervention and postintervention adherence with the 5 Moments for Hand Hygiene, as suggested by the World Health Organization, among health care workers from April 23 to May 25, 2018. A novel data collection form, the Hand Hygiene Observation Tool, was developed in opensource software and used to measure adherence with hand hygiene guidelines among health care workers in the inpatient therapeutic feeding center and pediatric ward of Anka General Hospital, Anka, Nigeria, and the postoperative ward of Noma Children’s Hospital, Sokoto, Nigeria. Qualitative data were analyzed throughout data collection and used for immediate feedback to staff. A more formal analysis of the data was conducted during October 2018.” |
| Martinez, 2018 – 761 [32] | Resource management + utilization tracking | “National and regional accrediting bodies including the National Committee for Quality Assurance, the Joint Commission on the Accreditation of Health Care Organizations, and the Health Services Cost Review Commission request that hospitals prove the clinical quality and cost effectiveness of the care they provide [37–41]. In addition to some of the metrics requested by the previous accrediting bodies, an extra set of operational and financial metrics were included in the eDashboard based on the strategic goals of our institution… Nonetheless, some of the metrics included in the eDashboard were accountability measures endorsed by national and regional accrediting bodies including The Joint Commission, supported by evidence, and associated with improved patient outcomes.” |
| Minton, 2020 – 1165 [33] | Performance monitoring | “We are a palliative care team hospital based in a teaching hospital that sees 1700 deaths approximately per year. We are active in quality improvement around end-of-life care but have found the requirements of the national end-of-life audit to be very onerous. While the overall recommendations can be used at a regional level there is little specific data that can be used for continuous quality improvement. We felt a locally designed tool would provide this and usefully supplement the national data set. Methods We used the larger national data set and after an iterative process with a range of junior doctors to design a simple spreadsheet with 20 questions that could be completed on an ad hoc rolling basis to monitor trends over time and to allow for thematic feedback to teams we focused on the areas of recognition of the dying, communication, treatment escalation planning and the five priorities of care of a dying patient. We also gave an overall impression of end of life based on the Royal College of Physicians’ structured judgement reviews… We have produced a dashboard of our main findings (see figure 1). The main findings are related to length of stay and recognition of dying is heavily skewed towards admission with most people dying within 5 days of it being recognised showing some variability in the dying process as we would expect.” |
| Mooney, 2019 – 3016 [34] | Care coordination + direct patient care | "The SCH system provides a DSS embedded with the data dashboard (Fig. 2). The DSS provides follow-up symptom assessment and intervention strategies based on published guidelines from several professional organizations. The primary source for the DSS development was the National Comprehensive Cancer Network Supportive Care Guidelines. Other sources included the Clinical Practice Guidelines for the Prevention and Treatment of Mucositis from the Multidisciplinary Association for Supportive Care in Cancer, the Oncology Nursing Society Putting Evidence Into Practice Resources program, the Physician Data Query resources provided by the National Cancer Institute, and Cancer Care Ontario Symptom Management Guidelines." |
| Mulhall, 2020 – 1030 [35] | Performance monitoring | “Since 2011, Health Quality Ontario (HQO) has been designated as the provincial lead on the quality of healthcare in Ontario with a mandate to report to the public, organizations, government, and healthcare providers on health system performance…The passage of the Long-Term Care Homes Act 2007 began the process of enhancing the ability to understand and improve quality in Ontario’s LTC homes. Implementation of this new legislation was coupled with the requirement that HQO report on quality in the homes. In addition, a reliable new source of relevant data was implemented first partially and then fully by 2010. The Resident Assessment Instrumente Minimum Data Set 2.0 (RAI-MDS 2.0) was introduced to standardize the assessment and care planning of residents in LTC homes. HQO currently reports 7 indicators of quality in LTC homes on its public website” |
| Newberry, 2019 – 1105 [107] | Population management + direct patient care | “Intervention efforts began with the development of a multidisciplinary task force consisting of cardiologists, gastroenterologists, pharmacy leads, and nursing specialists. This task force confirmed the need for a standardized guideline for acid suppression therapy to improve provider prescribing habits and ensure an evidence-based approach to care delivery. Through a series of consensus meetings, which analyzed published literature and national society recommendations, a guideline was created for the unit.5,6… An electronic dashboard was created in late February 2017 that risk-stratified patients based on prespecified guideline criteria (Figure 3). The dashboard interfaced with the existing electronic medical record (EMR) (Eclipsys Sunrise Clinical Manager Enterprise) to pull information in real time so that it was available during rounding.” |
| Palin, 2020 – 1115 [38] | Performance monitoring | "To help optimise antimicrobial use in primary care, the 2016/17 CQUIN aimed to reduce antibiotic consumption and encouraged a prescribing review within 72hours of commencing antibiotic treatment. QP measures for 2018/2019 also aimed to reduce AMR by targeting: (1) a reduction in gram negative blood stream infections, (2) a reduction of inappropriate antibiotic prescribing for urinary tract infections (UTI) in primary care and (3) a sustained reduction of inappropriate prescribing in primary care, based on the UK government targets of halving inappropriate prescribing by 2020/2021." |
| Patel, 2018 – 771 [89] | Population management | “Primary care physicians in the 2 intervention arms were emailed a link to an automated online dashboard listing their patients who met national guidelines for statin therapy but had not been prescribed this medication. The dashboard included relevant patient information, and for each patient, PCPs were asked to make an active choice to prescribe atorvastatin, 20 mg, once daily, atorvastatin at another dose, or another statin or not prescribe a statin and select a reason… The email provided the PCP with a link to a dashboard on a secure website that listed each of these patients along with their age, sex, and the following data as available from the EHR: 10-year ASCVD risk score, most recent LDL-C level, other lipid levels (ie, total cholesterol, high-density lipoprotein, and triglycerides), body mass index, history of smoking and any form of clinical ASCVD (eg, myocardial infarction or stroke), liver function tests (normal or slightly elevated), and medical record number (for EHR lookup if warranted). The PCPs were asked to review the list of patients within 1 week and use the dashboard to select whether or not to prescribe each patient a statin. The dashboard provided an overview of the study, a link to the American College of Cardiology/ American Heart Association guidelines, and options for selecting statin dosage.” |
| Patel, 2019 – 891 [75] | Resource management, utilization tracking + performance monitoring | “We sought to develop and implement a Maternity Dashboard to improve the quality of health care at the ground level… The selection of quality indicators was based on the prototype of clinical outcomes from the Royal College of Obstetricians and Gynecologists. The Maternity Dashboard team adapted local parameters and used preselected general parameters, based on clinical observations, to develop the dashboard.” |
| Rea, 2020 – 3496 [40] | Population management + direct patient care + performance monitoring | “The National Diabetes Audit (NDA) provides an annual snapshot of the quality of care received by people with diabetes…The Oxfordshire diabetes dashboard is based on the NDA and provides a monthly view of care processes received and treatment targets achieved by individual practice, Locality and primary care network compared to clinical commissioning group (CCG) average, national average and top CCG decile.” |
| Rezaei-Hachesu, 2018 – 666 [41] | Population management + direct patient care | “Introduction: Neonatal intensive care units (NICUs) have complex patients in terms of their diagnoses and required treatments. Antimicrobial treatment is a common therapy for patients in NICUs. To solve problems pertaining to empirical therapy, antimicrobial stewardship programs have recently been introduced. Despite the success of these programs in terms of data collection, there is still inefficiency in terms of analyzing and reporting the data. Thus, to successfully implement these stewardship programs, the design of antimicrobial resistance (AMR) surveillance systems is recommended as a first step. As a result, this study aimed to design an AMR surveillance system for use in the NICUs in northwestern Iranian hospitals to cover these information gaps.  Methods: The recommended system is compatible with the World Health Organization (WHO) guidelines. The business intelligence (BI) requirements were extracted in an interview with a product owner (PO) using a valid and reliable checklist.” |
| Rostata-Pesola, 2020 – 1162 [98] | Population management | “The purpose of this quality improvement project was to try to increase screening rates for HCV in the target population. One-time HCV screening for “baby boomers” is now required by New York State law (Appendix A, Supplemental Digital Content 1, http://links.lww.com/- JAANP/A42). The investigators implemented a provider education plan within a Veterans Affairs hospital clinic to improve the proportion of patients screened… The project was a pre and postintervention design. After a period of three months to establish baseline HCV screening proportions in several clinics, two types of interventions were implemented. One intervention was a targeted provider education for HCV screening presented as a lecture. A second intervention was the lecture and addition of an interactive hepatitis C screening dashboard. The providers were given both access and navigation training. Screening proportions were determined before and after intervention.” |
| Ruff, 2018 – 626 [43] | Population management + utilization tracking + performance monitoring | “ADP [American Dental Partners, Inc.] developed CRA [caries risk assessment] tools informed by nationally recognized models (5,18-21) and customizable risk-based caries management protocols (CMPs) grounded in evidence-based guidelines (5,18,20,22). Both were developed with significant clinician input, within the broader construct of the group’s quality assurance functions, to enable customization of the tools for the providers in the context of their clinical workflow. The CRAR incorporates CRA tools and CMPs as clinical decision support tools integrated within the EDR.. Dashboard indicator specification The selected indicators reflect the clinical care protocols and were prioritized as most relevant and actionable among a broader set considered. The indicators are intended for use as a coherent set and address patients’ registration status, CRA classification, receipt of routine and preventive care, caries related treatment and management, and resource use (Table 2). Specifications were developed for each indicator to define measurement.” |
| Smalley, 2019 – 1144 [102] | Performance monitoring | “In alignment with state guidelines, opioid prescription policies were implemented into the EMR as follows: a maximum of 30 mg of MEDD was mandated, all controlled substances required an associated diagnosis on the written prescription, and documentation of an exception to the acute pain prescribing rules were required either on the prescription or within the EMR. State legislation limited acute pain episodes to maximum of a seven-day supply of opioids in adults, and a five-day supply in pediatrics… As part of the intervention, data was collected on reasons for clinician override of active alerts, overall prescribing preferences of clinicians for acute complaints seen in the ED, and data on clinician outliers. Clinicians were then given access and direct feedback regarding their prescribing practices through use of an electronic dashboard. Direct feedback was a two-part process. First, monthly dashboards accessible to all clinicians tracked each clinician’s number of total opioid prescriptions, number of prescriptions exceeding a three-day supply (defined as 12 doses), number of prescriptions exceeding 30 MEDD, and the number of non-formulary prescriptions. Clinicians were sent an email with a direct link to the dashboard once per month where they could review their own metrics and compare to the healthcare system as a whole. Clinicians who were outliers for 2 months received direct, active feedback and coaching in person regarding their prescribing habits.” |
| Srivastava, 2019 – 890 [109] | Population management + care coordination | “Introduction: Centers for Disease Control and Prevention Diabetes Prevention Program recognition requires successful program completion by a cohort of at least five people with prediabetes. Such programs have generally been “in-person” and provided by a qualified coach from a recognized program. A cohort of 10 patients with prediabetes was enrolled in a physician’s office to use the cloud-based Type II Diabetes Prevention Module in an effort to achieve recognition. Module use was supported by the physician and a qualified coach. The purpose of this article is to evaluate Module performance relative to behavior stages associated with long-term behavior modification.  Methods: The Module employs a web application supporting diabetes prevention education and a mobile application that is an electronic diary and virtual coach. A dashboard allows an efficient review of user performance and the ability to send users notifications of support from the user’s coach or physician.” |
| Staples, 2020 – 1189 [80] | Resource management, utilization tracking + performance monitoring | Per table 1 - Appropriateness of blood transfusions was based on NICE guidelines  “Data for the blood product orders for RBC units and PLT units for hematology ward patients are generated by using the Cerner Discern Analytics (Millennium, Cerner Corporation) reporting tool and analyzed for compliance with the local guidelines for blood product transfusion for hematology patients. A presentation is compiled each month that incorporates these data with education and updates relevant for blood product ordering…Access to reporting dashboards is given to clinical staff to view by clinical specialty and additional dashboards are available showing further details including the clinical diagnosis at the time of the order being placed and order level-details including patient details, ordering clinician details, date, time, clinical details, and the justification for the transfusion provided at the time of the blood order.” |
| Twohig, 2019 – 729 [97] | Population management + performance monitoring | "To represent quality, we chose colorectal cancer (CRC) screening rates and percentage of patients with diabetes who had an A1c greater than 9% or no A1c in the past year. To be up to date on colorectal cancer screening, patients needed to have had a colonoscopy in the last 10 years or a fecal immunochemical test done in the last one year. We chose these two metrics out of many possibilities because they were clinically meaningful, able to be impacted by clinicians, aligned with value-based contracts such as our Medicare Accountable Care Organization, and we perform below national benchmarks as a system." |
| Tyler, 2018 – 663 [52] | Utilization tracking + performance monitoring | “The American Academy of Pediatrics published bronchiolitis clinical practice guidelines in 2014 recommending against the routine use of bronchodilators, chest radiographs, or respiratory viral testing in children with a clinical diagnosis of bronchiolitis. Our aim in this project was to align care with the American Academy of Pediatrics clinical practice guidelines by decreasing the overuse of these interventions. This study included patients who were admitted to a non-ICU setting with a primary or secondary diagnosis of bronchiolitis. The team used a multidisciplinary kickoff event to understand the problem and develop interventions, including sharing provider-specific data and asking providers to sign a pledge to reduce use. We used a novel, real-time data dashboard to collect and analyze data.” |
| Wang, 2020 – 1180 [54] | Population management + performance monitoring | “The Division of Rheumatology created an Opioid Working Group to address its goals of optimizing treatment of pain while lowering the risk of harm…Process measures were selected based on the CDC Guideline for Prescribing Opioids for Chronic Pain, Commonwealth of Pennsylvania guidelines, and New Jersey state law. These measures included the percentage of patients receiving chronic opioid prescriptions (> 3 prescriptions in a 12-month period) through the Division of Rheumatology who: 1) had an active opioid agreement within 12 months of their most recent opioid prescription (a Pennsylvania-specific guideline and New Jersey law); 2) had a visit with the provider within 3 months of their most recent opioid prescription; 3) had a urine drug screen within 12 months of their most recent opioid prescription; and 4) had an active concurrent benzodiazepine prescription [8,10,11]. In addition, the total number of opioid tablets prescribed per month was documented, with the expectation that increased compliance with these process measures would lead to a decrease in this number. We focused on Pennsylvania opioid prescribing guidelines and New Jersey opioid prescribing law because the vast majority of our patients’ taking opioids fill their prescriptions in one of these two states…UPHS created an Opioid Dashboard page that was integrated into the electric health record (EHR) system (Epic Systems Corporation, Verona, WI) which recorded the process measures above for each provider.” |
| Webers, 2019 – 968 [55] | Care coordination + direct patient care | "Domains and instruments were selected from existing Assessment of SpondyloArthritis international Society/Outcome Measures in Rheumatology (ASAS/ OMERACT) and Group for Research and Assessment of Psoriasis and Psoriatic Arthritis/OMERACT (GRAPPA/ OMERACT) sets,19 20 and several other disease-specific as well as generic domains and instruments were added." |

# Supplementary Table 10. Theoretical Frameworks Used to Guide Design, Implementation, or Evaluation of Healthcare Dashboards

| Study Author, Publication Year – Study ID | Theoretical Framework(s) | How Framework(s) Were Applied |
| --- | --- | --- |
| Ahern, 2020 – 1098 [1] | National Model Clinical Governance Framework | The dashboard was designed to align with the National Model Clinical Governance Framework national standards for supporting effective governance and improving safety and quality of health care. Specifically, the dashboard provides a clear visual display of clinical quality registry use and reporting to support integration of registry reporting into routine workflow and maximize the value of existing health data. |
| Barbeito, 2019 – 1020 [3] | Human Centered Design Framework | A human centered design framework was utilized to conduct post-implementation summative usability testing through “think aloud” usability evaluations with attending anesthesiologists. |
| Begum, 2020 – 1101 [84] | Grounded Theory Approach | The grounded theory approach was used to guide qualitative research at every stage, including data collection, coding, and analysis. |
| Chaparro, 2020 – 1028 [9] | Institute for Healthcare Improvement’s Model for Improvement | The Institute for Healthcare Improvement’s Model for Improvement was leveraged to inform the root cause analysis, rapid cycle testing, and selection of interventions and implementation strategies to reduce interruptive alert burden for all providers at Nationwide Children’s Hospital in Columbus, Ohio. |
| Dixit, 2020 – 1187 [12] | A human factors-based theoretical model of situation awareness | A human factors-based theoretical model of situation awareness informed the conceptualization, user needs assessment, and iterative development of dashboards to support telehealth initiatives  at the level of the telehealth management team, operational leadership, and executive leadership. |
| Dolan, 2019 – 792 [13] | - Theory of Change  - Logic Model Framework | The multi-level quality improvement project to improve controlled substance documentation discrepancies was based on the Theory of Change improvement framework and utilized a logic model framework approach to guide the iterative improvement and re-evaluation process. |
| Durojaiye, 2018 – 686 [64] | Experience Application Programming Interface (xAPI) Framework | The radiology resident dashboard was designed around ACGME milestones and the xAPI framework was the reference framework for mapping actions to relevant data sources and learning types and for implementing the dashboard to support resident training and education. |
| Findlay, 2020 – 1092 [16] | Consolidated Framework for Implementation Research | A multi-level implementation plan informed by CFIR was used to support the pre-treatment clinic and nutrition care dashboard interventions. The CFIR informed implementation strategies included use of clinical practice change strategies, multidisciplinary teams, integrated care, information technology, audit and feedback, staff education and support, and opinion leader engagement. |
| Fischer, 2020 – 1068 [17] | Technology Acceptance Model | To support dashboard maintenance and perform a summative usability assessment, the dashboard development team surveyed end-users to evaluate perceived ease of use and usefulness of the dashboard, with the questionnaire developed around the constructs outlined in the Technology Acceptance Model. |
| Fortuna, 2018 – 657 [85] | - Grounded Theory Approach  - Bio-Psychosocial-Spiritual Framework  - Social Justice Framework | The qualitative analysis of peer care notes entered onto the peer care management dashboard was informed by the grounded theory method. In addition, certified peer specialists used a bio-psychosocial-spiritual framework to guide their practice and encourage a focus on multi-level determinants of mental and physical health and a social justice framework to enrich the program through increased advocacy and assessments of unmet patient needs. |
| Fuller, 2020 – 1090 [20] | Reach, Effectiveness, Adoption, Implementation, and Maintenance (RE-AIM) Framework | The RE-AIM framework was used to identify relevant issues related to implementation, feasibility, and acceptability of health interventions. The framework informed the mixed-methods evaluation of the use, adoption, and perceived usability of the patient-centered discharge toolkit intervention which included integration of a bedside display of expected discharge date, discharge checklist, patient education, and a clinician-facing dashboard. |
| Jeffries, 2018 – 700 [111] | Normalization Process Theory | Qualitative interviews assessing physician and pharmacist perceptions of the medication safety dashboard were scheduled, coded, and thematically analyzed based on the principles of normalization process theory. |
| Martinez, 2018 – 761 [32] | Donabedian Model | The project team identified key performance indicators for the patient flow dashboard through interviews with unit and service level management and hospital leadership. The indicators were then organized into categories based on the Donabedian model of patient safety management, which the authors note to be a preferred framework of the Agency for Healthcare Research & Quality. |
| Mulhall, 2020 – 1030 [35] | - RAND/UCLA Appropriateness Method  - Clinical Performance Feedback Intervention Theory | Topics and indicators for the dashboard reports were selected using a modified Delphi process based on the RAND/UCLA Appropriateness Method. Dashboard development also aligned closely with the CP-FIT theory of audit and feedback. |
| Ni, 2019 – 899 [88] | Human Factors Engineering Framework | A human-factors engineering framework which involved an iterative user-centered design process to develop the dashboard and design the recruitment workflow and automated patient screening process was utilized. |
| Offodile, 2020 – 1058 [96] | Theory of Behavioral Economics | The project team leveraged the theory of behavioral economics to develop non-financial incentives, including dashboard views of performance data with peer comparisons, to prompt and incentivize surgeons to improve supply-chain management and reduce unnecessary operating room costs through modification of ordering behavior. |
| Patel, 2018 – 648 [69] | Programme Theory | The authors and project team reference prior work to develop a programme theory to identify factors which promote or hinder implementation of audit and feedback tools or the desired behavior change. The programme theory mapping informed development and implementation of the dashboard and STAT round education interventions. |
| Patel, 2018 – 771 [89] | Active Choice Principles | The active choice method which involves prompting an immediate decision between alternate choices to address delays in decision making informed the development and design of the automated dashboard of patients eligible for statin therapy. |
| Rezaei-Hachesu, 2018 – 666 [41] | Scrum for Research (SCORE) Framework | The NICU antimicrobial resistance dashboard was designed based on Scrum methodology, with key requirements identified through interviews conducted with a reliable and valid checklist and user evaluations, implementation of weekly sprints and daily meetings with content experts and use of summary reports to document development team discussions. |
| Rogal, 2020 – 1121 [92] | - Expert Recommendations for Implementation Research (ERIC) Framework  - Glasgow et al.’s Analytical Framework | The project team surveyed VA facilities nation-wide to assess each facilities use of 68 implementation strategies in the ERIC taxonomy in response to 1 of 2 versions of a policy-notice mandating case-reviews for patients prescribed opioids and at high risk for adverse outcomes and identified on the STORM dashboard. Additionally, organizational factors for each facility were examined in line with Glasgow et al.’s analytic framework to account for facility rurality and staffing or culture variables that may promote or impede quality improvement efforts. |
| Ryskina, 2019 – 1148 [106] | Social Comparison Feedback | Social comparison feedback principles were incorporated into the personalized utilization dashboard that was shared with resident physicians in an attempt to reduce low value laboratory test ordering. |
| Schleyer, 2019 – 896 [45] | - Unified Theory of Acceptance and Use of Technology Model (UTAUT)  - DeLone and McLean Information System Success Model | The mixed-methods evaluation of the chest pain dashboard which integrated health information exchange data into the EHR system in an emergency department was guided by the UTAUT and D&M IS success model frameworks to assess dashboard acceptance and conceptualize dashboard success. |
| Srivastava, 2019 – 890 [109] | Stages of Change Model | The stages of change model informed the design and implementation of the patient facing diabetes prevention mhealth tool and the diabetes coach and physician supported dashboard interventions. |
| Stachelek, 2020 – 1080 [49] | Disruptive Behavior Pyramid Theory | The dashboard was developed based on the Disruptive Behavior Pyramid Theory to regularly provide personalized performance data to each physician with a step-wise plan for leadership-delivered feedback and performance improvement planning when providers failed to meet quarterly compliance goals. |

# References

1. Ahern S, Feiler R, Sdrinis S. Maximising the value of clinical registry information through integration with a health service clinical governance framework: a case study. Aust Health Rev Publ Aust Hosp Assoc Australia; 2020;44(3):421–426. doi: 10.1071/AH19004

2. Bae YS, Kim KH, Choi SW, Ko T, Jeong CW, Cho B, Kim MS, Kang E. Information Technology-Based Management of Clinically Healthy COVID-19 Patients: Lessons From a Living and Treatment Support Center Operated by Seoul National University Hospital. J Med Internet Res Canada; 2020;22(6):e19938. doi: 10.2196/19938

3. Barbeito A, Segall N. Development and usability testing of an audit and feedback tool for anesthesiologists. JAMIA Open United States; 2019;2(1):29–34. doi: 10.1093/jamiaopen/ooy054

4. Barnett A, Winning M, Canaris S, Cleary M, Staib A, Sullivan C. Digital transformation of hospital quality and safety: real-time data for real-time action. Aust Health Rev Publ Aust Hosp Assoc Australia; 2019;43(6):656–661. doi: 10.1071/AH18125

5. Bersani K, Fuller TE, Garabedian P, Espares J, Mlaver E, Businger A, Chang F, Boxer RB, Schnock KO, Rozenblum R, Dykes PC, Dalal AK, Benneyan JC, Lehmann LS, Gershanik EF, Bates DW, Schnipper JL. Use, Perceived Usability, and Barriers to Implementation of a Patient Safety Dashboard Integrated within a Vendor EHR. Appl Clin Inform Germany; 2020;11(1):34–45. doi: 10.1055/s-0039-3402756

6. Brink AJ, Messina AP, Maslo C, Swart K, Chunnilall D, van den Bergh D, Netcare Antimicrobial Stewardship and Infection Prevention Study Alliance. Implementing a multi-faceted framework for proprietorship of hand hygiene compliance in a network of South African hospitals: leveraging the Ubuntu philosophy. J Hosp Infect England; 2020;104(4):404–413. doi: 10.1016/j.jhin.2019.11.004

7. Burningham Z, Jackson G, Kelleher J, Stevens M, Morris I, Cohen J, Maloney G, Vaughan C. The Enhancing Quality of Prescribing Practices for Older Veterans Discharged From the Emergency Department (EQUIPPED) Potentially Inappropriate Medication Dashboard: a Suitable Alternative to the In-Person Academic Detailing and Standardized Feedback Rep. 2020; doi: 10.1016/j.clinthera.2020.02.013

8. Cassim N, Tepper ME, Coetzee LM, Glencross DK. Timely delivery of laboratory efficiency information, Part I: Developing an interactive turnaround time dashboard at a high-volume laboratory. Afr J Lab Med South Africa; 2020;9(2):947. doi: 10.4102/ajlm.v9i2.947

9. Chaparro JD, Hussain C, Lee JA, Hehmeyer J, Nguyen M, Hoffman J. Reducing Interruptive Alert Burden Using Quality Improvement Methodology. Appl Clin Inform Germany; 2020;11(1):46–58. doi: 10.1055/s-0039-3402757

10. Coventry P, Bower P, Blakemore A, Baker E, Hann M, Li J, Paisley A, Gibson M. Satisfaction with a digitally-enabled telephone health coaching intervention for people with non-diabetic hyperglycaemia. NPJ Digit Med England; 2019;2(101731738):5. doi: 10.1038/s41746-019-0080-6

11. Dagliati A, Sacchi L, Tibollo V, Cogni G, Teliti M, Martinez-Millana A, Traver V, Segagni D, Posada J, Ottaviano M, Fico G, Arredondo MT, De Cata P, Chiovato L, Bellazzi R. A dashboard-based system for supporting diabetes care. J Am Med Inform Assoc JAMIA England; 2018;25(5):538–547. doi: 10.1093/jamia/ocx159

12. Dixit RA, Hurst S, Adams KT, Boxley C, Lysen-Hendershot K, Bennett SS, Booker E, Ratwani RM. Case Report: Rapid Development of Visualization Dashboards to Enhance Situation Awareness of COVID-19 Telehealth Initiatives at a Multi-Hospital Healthcare System. J Am Med Inform Assoc JAMIA England; 2020;(b92, 9430800). doi: 10.1093/jamia/ocaa161

13. Dolan JE, Lonsdale H, Ahumada LM, Patel A, Samuel J, Jalali A, Peck J, DeRosa JC, Rehman M, Varughese AM, Fernandez AM. Quality Initiative Using Theory of Change and Visual Analytics to Improve Controlled Substance Documentation Discrepancies in the Operating Room. Appl Clin Inform Germany; 2019;10(3):543–551. doi: 10.1055/s-0039-1693688

14. Elm JJ, Daeschler M, Bataille L, Schneider R, Amara A, Espay AJ, Afek M, Admati C, Teklehaimanot A, Simuni T. Feasibility and utility of a clinician dashboard from wearable and mobile application Parkinson’s disease data. NPJ Digit Med England; 2019;2(101731738):95. doi: 10.1038/s41746-019-0169-y

15. Field M, Fong K, Shade C. Use of Electronic Visibility Boards to Improve Patient Care Quality, Safety, and Flow on Inpatient Pediatric Acute Care Units. J Pediatr Nurs 2018;41:69–76. doi: 10.1016/j.pedn.2018.01.015

16. Findlay M, Rankin NM, Shaw T, White K, Boyer M, Milross C, De Abreu Lourenco R, Brown C, Collett G, Beale P, Bauer JD. Best Evidence to Best Practice: Implementing an Innovative Model of Nutrition Care for Patients with Head and Neck Cancer Improves Outcomes. Nutrients Switzerland; 2020;12(5). doi: 10.3390/nu12051465

17. Fischer MJ, Kourany WM, Sovern K, Forrester K, Griffin C, Lightner N, Loftus S, Murphy K, Roth G, Palevsky PM, Crowley ST. Development, implementation and user experience of the Veterans Health Administration (VHA) dialysis dashboard. BMC Nephrol England; 2020;21(1):136. doi: 10.1186/s12882-020-01798-6

18. Fletcher G.S., Aaronson B.A., White A.A., Julka R. Effect of a Real-Time Electronic Dashboard on a Rapid Response System. J Med Syst 2018;42(1). doi: 10.1007/s10916-017-0858-5

19. Frymoyer A, Schwenk HT, Zorn Y, Bio L, Moss JD, Chasmawala B, Faulkenberry J, Goswami S, Keizer RJ, Ghaskari S. Model-Informed Precision Dosing of Vancomycin in Hospitalized Children: Implementation and Adoption at an Academic Children’s Hospital. Front Pharmacol Switzerland; 2020;11(101548923):551. doi: 10.3389/fphar.2020.00551

20. Fuller TE, Pong DD, Piniella N, Pardo M, Bessa N, Yoon C, Boxer RB, Schnipper JL, Dalal AK. Interactive Digital Health Tools to Engage Patients and Caregivers in Discharge Preparation: Implementation Study. J Med Internet Res Canada; 2020;22(4):e15573. doi: 10.2196/15573

21. Fuller TE, Garabedian PM, Lemonias DP, Joyce E, Schnipper JL, Harry EM, Bates DW, Dalal AK, Benneyan JC. Assessing the cognitive and work load of an inpatient safety dashboard in the context of opioid management. Appl Ergon England; 2020;85(cyz, 0261412):103047. doi: 10.1016/j.apergo.2020.103047

22. Giordanengo A, Arsand E, Woldaregay AZ, Bradway M, Grottland A, Hartvigsen G, Granja C, Torsvik T, Hansen AH. Design and Prestudy Assessment of a Dashboard for Presenting Self-Collected Health Data of Patients With Diabetes to Clinicians: Iterative Approach and Qualitative Case Study. JMIR Diabetes Canada; 2019;4(3):e14002. doi: 10.2196/14002

23. Grange ES, Neil EJ, Stoffel M, Singh AP, Tseng E, Resco-Summers K, Fellner BJ, Lynch JB, Mathias PC, Mauritz-Miller K, Sutton PR, Leu MG. Responding to COVID-19: The UW Medicine Information Technology Services Experience. Appl Clin Inform Germany; 2020;11(2):265–275. doi: 10.1055/s-0040-1709715

24. Hebert C, Flaherty J, Smyer J, Ding J, Mangino JE. Development and validation of an automated ventilator-associated event electronic surveillance system: A report of a successful implementation. Am J Infect Control United States; 2018;46(3):316–321. doi: 10.1016/j.ajic.2017.09.006

25. Hester G, Lang T, Madsen L, Tambyraja R, Zenker P. Timely Data for Targeted Quality Improvement Interventions: Use of a Visual Analytics Dashboard for Bronchiolitis. Appl Clin Inform Germany; 2019;10(1):168–174. doi: 10.1055/s-0039-1679868

26. Hoogeveen IJ, Peeks F, de Boer F, Lubout CMA, de Koning TJ, te Boekhorst S, Zandvoort R-J, Burghard R, van Spronsen FJ, Derks TGJ. A preliminary study of telemedicine for patients with hepatic glycogen storage disease and their healthcare providers: from bedside to home site monitoringKeywords. J Inherit Metab Dis 2018;41(6):929–936. doi: 10.1007/s10545-018-0167-2

27. Jameie S, Haybar H, Aslani A, Saadat M. Development and Usability Evaluation of Web-Based Telerehabilitation Platform for Patients After Myocardial Infarction. Stud Health Technol Inform Netherlands; 2019;261(ck1, 9214582):68–74.

28. Kummer BR, Willey JZ, Zelenetz MJ, Hu Y, Sengupta S, Elkind MSV, Hripcsak G. Neurological Dashboards and Consultation Turnaround Time at an Academic Medical Center. Appl Clin Inform Germany; 2019;10(5):849–858. doi: 10.1055/s-0039-1698465

29. Kunjan K, Doebbeling B, Toscos T. Dashboards to Support Operational Decision Making in Health Centers: A Case for Role-Specific Design. Int J Hum-Comput Interact 2019;35(9):742–750. doi: 10.1080/10447318.2018.1488418

30. Laurent G, Moussa MD, Cirenei C, Tavernier B, Marcilly R, Lamer A. Development, implementation and preliminary evaluation of clinical dashboards in a department of anesthesia. J Clin Monit Comput Netherlands; 2020;(9806357, cyw). doi: 10.1007/s10877-020-00522-x

31. Mabirizi D, Phulu B, Churfo W, Mwinga S, Mazibuko G, Sagwa E, Indongo L, Hafner T. Implementing an Integrated Pharmaceutical Management Information System for Antiretrovirals and Other Medicines: Lessons From Namibia. Glob Health Sci Pract United States; 2018;6(4):723–735. doi: 10.9745/GHSP-D-18-00157

32. Martinez DA, Kane EM, Jalalpour M, Scheulen J, Rupani H, Toteja R, Barbara C, Bush B, Levin SR. An Electronic Dashboard to Monitor Patient Flow at the Johns Hopkins Hospital: Communication of Key Performance Indicators Using the Donabedian Model. J Med Syst United States; 2018;42(8):133. doi: 10.1007/s10916-018-0988-4

33. Minton O, Ede C, Bass S, Tavabie S, Bourne A, Hiresche A. Hospital deaths dashboard: care indicators. BMJ Support Palliat Care England; 2020;(101565123). doi: 10.1136/bmjspcare-2020-002223

34. Mooney K., Whisenant M.S., Beck S.L. Symptom Care at Home: A Comprehensive and Pragmatic PRO System Approach to Improve Cancer Symptom Care. Med Care 2019;57((Mooney K., kathi.mooney@nurs.utah.edu) Huntsman Cancer Institute, University of Utah, 10 South 2000 East, Salt Lake City, UT, United States(Whisenant M.S.) Department of Symptom Research, University of Texas, MD Anderson Cancer Center, Houston, TX, Unite):S66–S72. doi: 10.1097/MLR.0000000000001037

35. Mulhall CL, Lam JMC, Rich PS, Dobell LG, Greenberg A. Enhancing Quality Care in Ontario Long-Term Care Homes Through Audit and Feedback for Physicians. J Am Med Dir Assoc United States; 2020;21(3):420–425. doi: 10.1016/j.jamda.2019.11.017

36. Nelson O, Sturgis B, Gilbert K, Henry E, Clegg K, Tan JM, Wasey JO, Simpao AF, Galvez JA. A Visual Analytics Dashboard to Summarize Serial Anesthesia Records in Pediatric Radiation Treatment. Appl Clin Inform Germany; 2019;10(4):563–569. doi: 10.1055/s-0039-1693712

37. Ospina-Pinillos L, Davenport TA, Ricci CS, Milton AC, Scott EM, Hickie IB. Developing a Mental Health eClinic to Improve Access to and Quality of Mental Health Care for Young People: Using Participatory Design as Research Methodologies. J Med Internet Res Canada; 2018;20(5):e188. doi: 10.2196/jmir.9716

38. Palin V, Tempest E, Mistry C, van Staa TP. Developing the infrastructure to support the optimisation of antibiotic prescribing using the learning healthcare system to improve healthcare services in the provision of primary care in England. BMJ Health Care Inform England; 2020;27(1). doi: 10.1136/bmjhci-2020-100147

39. Pandya ST, Chakravarthy K, Vemareddy A. Obstetric anaesthesia practice: Dashboard as a dynamic audit tool. Indian J Anaesth India; 2018;62(11):838–843. doi: 10.4103/ija.IJA_346_18

40. Rea R.D., Lumb A., Tan G.D., Owen K., Thanabalasingham G., Latif A., Swan P., Scott J., Jones D., Gillott E., Smith R.H. Using data to improve the care of people with diabetes across Oxfordshire. Pract Diabetes 2020;37(1):27–31. doi: 10.1002/pdi.2257

41. Rezaei-Hachesu P, Samad-Soltani T, Yaghoubi S, GhaziSaeedi M, Mirnia K, Masoumi-Asl H, Safdari R. The design and evaluation of an antimicrobial resistance surveillance system for neonatal intensive care units in Iran. Int J Med Inf Ireland; 2018;115(ct4, 9711057):24–34. doi: 10.1016/j.ijmedinf.2018.04.007

42. Romero-Brufau S, Kostandy P, Maass KL, Wutthisirisart P, Sir M, Bartholmai B, Stuve M, Pasupathy K. Development of data integration and visualization tools for the Department of Radiology to display operational and strategic metrics. AMIA Annu Symp Proc AMIA Symp United States; 2018;2018(101209213):942–951.

43. Ruff JC, Herndon JB, Horton RA, Lynch J, Mathwig DC, Leonard A, Aravamudhan K. Developing a caries risk registry to support caries risk assessment and management for children: A quality improvement initiative. J Public Health Dent United States; 2018;78(2):134–143. doi: 10.1111/jphd.12253

44. Scheinfeld MH, Feltus W, DiMarco P, Rooney K, Goldman IA. The Emergency Radiology Dashboard: Facilitating Workflow With Realtime Data. Curr Probl Diagn Radiol United States; 2020;49(4):231–233. doi: 10.1067/j.cpradiol.2020.02.013

45. Schleyer TKL, Rahurkar S, Baublet AM, Kochmann M, Ning X, Martin DK, Finnell JT, Kelley KW, FHIR Development Team, Schaffer JT. Preliminary evaluation of the Chest Pain Dashboard, a FHIR-based approach for integrating health information exchange information directly into the clinical workflow. AMIA Jt Summits Transl Sci Proc AMIA Jt Summits Transl Sci United States; 2019;2019(101539486):656–664.

46. Schmidt T, Brabrand M, Lassen AT, Wiil UK. Design and Evaluation of a Patient Monitoring Dashboard for Emergency Departments. Stud Health Technol Inform Netherlands; 2019;264(ck1, 9214582):788–792. doi: 10.3233/SHTI190331

47. Sheen Y-J, Huang C-C, Huang S-C, Huang M-D, Lin C-H, Lee I-T, Lin S-Y, Sheu WH-H. IMPLEMENTATION OF AN ELECTRONIC DASHBOARD WITH A REMOTE MANAGEMENT SYSTEM TO IMPROVE GLYCEMIC MANAGEMENT AMONG HOSPITALIZED ADULTS. Endocr Pract Off J Am Coll Endocrinol Am Assoc Clin Endocrinol United States; 2020;26(2):179–191. doi: 10.4158/EP-2019-0264

48. Sinvani L., Goldin M., Roofeh R., Idriss N., Goldman A., Klein Z., Mendelson D.A., Carney M.T. Implementation of Hip Fracture Co-Management Program (AGS CoCare: Ortho®) in a Large Health System. J Am Geriatr Soc 2020;((Sinvani L.; Goldin M.; Roofeh R.; Idriss N.) Division of Hospital Medicine, Department of Medicine, Northwell Health, Manhasset, NY, United States(Roofeh R.; Carney M.T., mcarney@northwell.edu) Division of Geriatrics and Palliative Medicine, Department o). doi: 10.1111/jgs.16483

49. Stachelek GC, McNutt T, Thompson CB, Smith K, DeWeese TL, Song DY. Improvements in Physician Clinical Workflow Measures After Implementation of a Dashboard Program. Pract Radiat Oncol United States; 2020;10(3):151–157. doi: 10.1016/j.prro.2019.11.014

50. Taber DJ, Pilch NA, McGillicuddy JW, Mardis C, Treiber F, Fleming JN. Using informatics and mobile health to improve medication safety monitoring in kidney transplant recipients. Am J Health-Syst Pharm AJHP Off J Am Soc Health-Syst Pharm England; 2019;76(15):1143–1149. doi: 10.1093/ajhp/zxz115

51. Trinh LD, Wong JT, Sullivan PA, Nguyen LH, Pham YT. Impact of a chemotherapy workload and productivity dashboard on pharmacy technician turnover. Am J Health-Syst Pharm AJHP Off J Am Soc Health-Syst Pharm England; 2019;76(13):992–997. doi: 10.1093/ajhp/zxz081

52. Tyler A, Krack P, Bakel LA, O’Hara K, Scudamore D, Topoz I, Freeman J, Moss A, Allen R, Swanson A, Bajaj L. Interventions to Reduce Over-Utilized Tests and Treatments in Bronchiolitis. Pediatrics United States; 2018;141(6). doi: 10.1542/peds.2017-0485

53. Van Citters AD, Gifford AH, Brady C, Dunitz JM, Elmhirst M, Flath J, Laguna TA, Moore B, Prickett ML, Riordan M, Savant AP, Gore W, Jian S, Soper M, Marshall BC, Nelson EC, Sabadosa KA. Formative evaluation of a dashboard to support coproduction of healthcare services in cystic fibrosis. J Cyst Fibros Off J Eur Cyst Fibros Soc Netherlands; 2020;(101128966). doi: 10.1016/j.jcf.2020.03.009

54. Wang EJ, Helgesen R, Johr CR, Lacko HS, Ashburn MA, Merkel PA. A Targeted Program in an Academic Rheumatology Practice to Improve Compliance with Opioid Prescribing Guidelines for the Treatment of Chronic Pain. Arthritis Care Res United States; 2020;(101518086). doi: 10.1002/acr.24354

55. Webers C, Beckers E, Boonen A, van Eijk-Hustings Y, Vonkeman H, van de Laar M, van Tubergen A. Development, usability and acceptability of an integrated eHealth system for spondyloarthritis in the Netherlands (SpA-Net). RMD Open England; 2019;5(1):e000860. doi: 10.1136/rmdopen-2018-000860

56. Williams R, Keers R, Gude WT, Jeffries M, Davies C, Brown B, Kontopantelis E, Avery AJ, Ashcroft DM, Peek N. SMASH! The Salford medication safety dashboard. J Innov Health Inform England; 2018;25(3):183–193. doi: 10.14236/jhi.v25i3.1015

57. Woo JS, Suslow P, Thorsen R, Ma R, Bakhtary S, Moayeri M, Nambiar A. Development and Implementation of Real-Time Web-Based Dashboards in a Multisite Transfusion Service. J Pathol Inform India; 2019;10(101528849):3. doi: 10.4103/jpi.jpi_36_18

58. Yoo J, Jung KY, Kim T, Lee T, Hwang SY, Yoon H, Shin TG, Sim MS, Jo IJ, Paeng H, Choi JS, Cha WC. A Real-Time Autonomous Dashboard for the Emergency Department: 5-Year Case Study. JMIR MHealth UHealth Canada; 2018;6(11):e10666. doi: 10.2196/10666

59. Gardner LA, Bray PJ, Finley E, Sterner C, Ignudo TL 3rd, Stauffer CL, Kincaid SA, Marella WM. Standardizing Falls Reporting: Using Data From Adverse Event Reporting to Drive Quality Improvement. J Patient Saf United States; 2019;15(2):135–142. doi: 10.1097/PTS.0000000000000204

60. Paulson SS, Dummett BA, Green J, Scruth E, Reyes V, Escobar GJ. What Do We Do After the Pilot Is Done? Implementation of a Hospital Early Warning System at Scale. Jt Comm J Qual Patient Saf Netherlands; 2020;46(4):207–216. doi: 10.1016/j.jcjq.2020.01.003

61. Whidden C, Kayentao K, Liu JX, Lee S, Keita Y, Diakite D, Keita A, Diarra S, Edwards J, Yembrick A, Holeman I, Samake S, Plea B, Coumare M, Johnson AD. Improving Community Health Worker performance by using a personalised feedback dashboard for supervision: a randomised controlled trial. J Glob Health Scotland; 2018;8(2):020418. doi: 10.7189/jogh.08.020418

62. Burns JL, Hasting D, Gichoya JW, McKibben B 3rd, Shea L, Frank M. Just in Time Radiology Decision Support Using Real-time Data Feeds. J Digit Imaging United States; 2020;33(1):137–142. doi: 10.1007/s10278-019-00268-2

63. Choi HH, Clark J, Jay AK, Filice RW. Minimizing Barriers in Learning for On-Call Radiology Residents-End-to-End Web-Based Resident Feedback System. J Digit Imaging 2018;31(1):117–123. doi: 10.1007/s10278-017-0015-1

64. Durojaiye AB, Snyder E, Cohen M, Nagy P, Hong K, Johnson PT. Radiology Resident Assessment and Feedback Dashboard. Radiogr Rev Publ Radiol Soc N Am Inc United States; 2018;38(5):1443–1453. doi: 10.1148/rg.2018170117

65. Tan A, Durbin M, Chung FR, Rubin AL, Cuthel AM, McQuilkin JA, Modrek AS, Jamin C, Gavin N, Mann D, Swartz JL, Austrian JS, Testa PA, Hill JD, Grudzen CR, Group Authorship: Corita R. Grudzen on behalf of the PRIM-ER Clinical Informatics Advisory Board. Design and implementation of a clinical decision support tool for primary palliative Care for Emergency Medicine (PRIM-ER). BMC Med Inform Decis Mak England; 2020;20(1):13. doi: 10.1186/s12911-020-1021-7

66. Robinson JR, Carter NH, Gibson C, Brinkman AS, Van Arendonk K, Speck KE, Danko ME, Jackson GP, Lovvorn HN 3rd, Blakely ML. Improving the value of care for appendectomy through an individual surgeon-specific approach. J Pediatr Surg United States; 2018;53(6):1181–1186. doi: 10.1016/j.jpedsurg.2018.02.081

67. Huber TC, Krishnaraj A, Monaghan D, Gaskin CM. Developing an Interactive Data Visualization Tool to Assess the Impact of Decision Support on Clinical Operations. J Digit Imaging United States; 2018;31(5):640–645. doi: 10.1007/s10278-018-0065-z

68. Hagaman DH, Ehrenfeld JM, Terekhov M, Kla KM, Hamm J, Brumley M, Wanderer JP. Compliance Is Contagious: Using Informatics Methods to Measure the Spread of a Documentation Standard From a Preoperative Clinic. J Perianesthesia Nurs Off J Am Soc PeriAnesthesia Nurses United States; 2018;33(4):436–443. doi: 10.1016/j.jopan.2016.08.016

69. Patel S, Rajkomar A, Harrison JD, Prasad PA, Valencia V, Ranji SR, Mourad M. Next-generation audit and feedback for inpatient quality improvement using electronic health record data: a cluster randomised controlled trial. BMJ Qual Saf England; 2018;27(9):691–699. doi: 10.1136/bmjqs-2017-007393

70. Connor JP, Raife T, Medow JE, Ehlenfeldt BD, Sipsma K. The blood utilization calculator, a target-based electronic decision support algorithm, increases the use of single-unit transfusions in a large academic medical center. Transfusion (Paris) United States; 2018;58(7):1689–1696. doi: 10.1111/trf.14637

71. van Zijl FVWJ, Versnel S, van der Poel EF, Baatenburg de Jong RJ, Datema FR. Use of Routine Prospective Functional and Aesthetic Patient Satisfaction Measurements in Secondary Cleft Lip Rhinoplasty. JAMA Facial Plast Surg United States; 2018;20(6):488–494. doi: 10.1001/jamafacial.2018.0876

72. Cassim N, Coetzee LM, Tepper MEE, Perelson L, Glencross DK. Timely delivery of laboratory efficiency information, Part II: Assessing the impact of a turn-around time dashboard at a high-volume laboratory. Afr J Lab Med South Africa; 2020;9(2):948. doi: 10.4102/ajlm.v9i2.948

73. Niendam TA, Tully LM, Iosif A-M, Kumar D, Nye KE, Denton JC, Zakskorn LN, Fedechko TL, Pierce KM. Enhancing early psychosis treatment using smartphone technology: A longitudinal feasibility and validity study. J Psychiatr Res England; 2018;96(jtj, 0376331):239–246. doi: 10.1016/j.jpsychires.2017.10.017

74. Bauer AM, Iles-Shih M, Ghomi RH, Rue T, Grover T, Kincler N, Miller M, Katon WJ. Acceptability of mHealth augmentation of Collaborative Care: A mixed methods pilot study. Gen Hosp Psychiatry United States; 2018;51(fnk, 7905527):22–29. doi: 10.1016/j.genhosppsych.2017.11.010

75. Patel MS, Rathi B, Tashfeen K, Yarubi MA. Development and Implementation of Maternity Dashboard in Regional Hospital for Quality Improvement at Ground Level: A Pilot Study. Oman Med J Oman; 2019;34(3):194–199. doi: 10.5001/omj.2019.38

76. Lenglet A, van Deursen B, Viana R, Abubakar N, Hoare S, Murtala A, Okanlawon M, Osatogbe J, Emeh V, Gray N, Keller S, Masters P, Roolvink D, Davies J, Hickox K, Fotso A, Bil K, Ikenna Nwankwo C, Ahmad B, Caluwaerts A, Lessard I, Dimeglio S, Malou N, Kanapathipillai R, McRae M, Wong S, Hopman J. Inclusion of Real-Time Hand Hygiene Observation and Feedback in a Multimodal Hand Hygiene Improvement Strategy in Low-Resource Settings. JAMA Netw Open United States; 2019;2(8):e199118. doi: 10.1001/jamanetworkopen.2019.9118

77. Harzand A, Witbrodt B, Davis-Watts ML, Alrohaibani A, Goese D, Wenger NK, Shah AJ, Zafari AM. Feasibility of a Smartphone-enabled Cardiac Rehabilitation Program in Male Veterans With Previous Clinical Evidence of Coronary Heart Disease. Am J Cardiol United States; 2018;122(9):1471–1476. doi: 10.1016/j.amjcard.2018.07.028

78. Kumar D, Tully LM, Iosif A-M, Zakskorn LN, Nye KE, Zia A, Niendam TA. A Mobile Health Platform for Clinical Monitoring in Early Psychosis: Implementation in Community-Based Outpatient Early Psychosis Care. JMIR Ment Health Canada; 2018;5(1):e15. doi: 10.2196/mental.8551

79. Shailam R, Botwin A, Stout M, Gee MS. Real-Time Electronic Dashboard Technology and Its Use to Improve Pediatric Radiology Workflow. Curr Probl Diagn Radiol United States; 2018;47(1):3–5. doi: 10.1067/j.cpradiol.2017.03.002

80. Staples S, Salisbury RA, King AJ, Polzella P, Bakhishli G, Staves J, Murphy MF. How do we use electronic clinical decision support and feedback to promote good transfusion practice. Transfusion (Paris) United States; 2020;(wdn, 0417360). doi: 10.1111/trf.15864

81. Graber CJ, Jones MM, Goetz MB, Madaras-Kelly K, Zhang Y, Butler JM, Weir C, Chou AF, Youn SY, Samore MH, Glassman PA. Decreases in antimicrobial use associated with multihospital implementation of electronic antimicrobial stewardship tools. Clin Infect Dis Off Publ Infect Dis Soc Am United States; 2019;(a4j, 9203213). doi: 10.1093/cid/ciz941

82. Smith AJF, Ahern SP, Shermock KM, Feller TT, Hill JD. Assessment of impact of daily huddles and visual displays on medication delivery timeliness in a large academic medical center. Am J Health-Syst Pharm AJHP Off J Am Soc Health-Syst Pharm England; 2020;(9503023, cbh). doi: 10.1093/ajhp/zxaa210

83. Jung AD, Baker J, Droege CA, Nomellini V, Johannigman J, Holcomb JB, Goodman MD, Pritts TA. Sooner is better: use of a real-time automated bedside dashboard improves sepsis care. J Surg Res United States; 2018;231(k7b, 0376340):373–379. doi: 10.1016/j.jss.2018.05.078

84. Begum T, Khan SM, Adamou B, Ferdous J, Parvez MM, Islam MS, Kumkum FA, Rahman A, Anwar I. Perceptions and experiences with district health information system software to collect and utilize health data in Bangladesh: a qualitative exploratory study. BMC Health Serv Res England; 2020;20(1):465. doi: 10.1186/s12913-020-05322-2

85. Fortuna KL, Storm M, Aschbrenner KA, Bartels SJ. Integration of Peer Philosophy into a Standardized Self-Management Mobile Health Intervention. Psychiatr Q United States; 2018;89(4):795–800. doi: 10.1007/s11126-018-9578-3

86. Herzke CA, Michtalik HJ, Durkin N, Finkelstein J, Deutschendorf A, Miller J, Leung C, Brotman DJ. A Method for Attributing Patient-Level Metrics to Rotating Providers in an Inpatient Setting. J Hosp Med United States; 2018;13(7):470–475. doi: 10.12788/jhm.2897

87. Jeffries M, Gude WT, Keers RN, Phipps DL, Williams R, Kontopantelis E, Brown B, Avery AJ, Peek N, Ashcroft DM. Understanding the utilisation of a novel interactive electronic medication safety dashboard in general practice: a mixed methods study. BMC Med Inform Decis Mak England; 2020;20(1):69. doi: 10.1186/s12911-020-1084-5

88. Ni Y, Bermudez M, Kennebeck S, Liddy-Hicks S, Dexheimer J. A Real-Time Automated Patient Screening System for Clinical Trials Eligibility in an Emergency Department: Design and Evaluation. JMIR Med Inform Canada; 2019;7(3):e14185. doi: 10.2196/14185

89. Patel MS, Kurtzman GW, Kannan S, Small DS, Morris A, Honeywell SJ, Leri D, Rareshide CAL, Day SC, Mahoney KB, Volpp KG, Asch DA. Effect of an Automated Patient Dashboard Using Active Choice and Peer Comparison Performance Feedback to Physicians on Statin Prescribing: The PRESCRIBE Cluster Randomized Clinical Trial. JAMA Netw Open United States; 2018;1(3):e180818. doi: 10.1001/jamanetworkopen.2018.0818

90. Reback CJ, Runger D, Fletcher JB, Swendeman D. Ecological momentary assessments for self-monitoring and counseling to optimize methamphetamine treatment and sexual risk reduction outcomes among gay and bisexual men. J Subst Abuse Treat United States; 2018;92(kai, 8500909):17–26. doi: 10.1016/j.jsat.2018.06.005

91. Reszel J, Dunn SI, Sprague AE, Graham ID, Grimshaw JM, Peterson WE, Ockenden H, Wilding J, Quosdorf A, Darling EK, Fell DB, Harrold J, Lanes A, Smith GN, Taljaard M, Weiss D, Walker MC. Use of a maternal newborn audit and feedback system in Ontario: a collective case study. BMJ Qual Saf England; 2019;28(8):635–644. doi: 10.1136/bmjqs-2018-008354

92. Rogal SS, Chinman M, Gellad WF, Mor MK, Zhang H, McCarthy SA, Mauro GT, Hale JA, Lewis ET, Oliva EM, Trafton JA, Yakovchenko V, Gordon AJ, Hausmann LRM. Tracking implementation strategies in the randomized rollout of a Veterans Affairs national opioid risk management initiative. Implement Sci IS England; 2020;15(1):48. doi: 10.1186/s13012-020-01005-y

93. Stranne J, Axen E, Franck-Lissbrant I, Fransson P, Franlund M, Hugosson J, Khatami A, Koss-Modig K, Lodding P, Nyberg M, Stattin P, Bratt O. Single institution followed by national implementation of systematic surgical quality control and feedback for radical prostatectomy: a 20-year journey. World J Urol Germany; 2020;38(6):1397–1411. doi: 10.1007/s00345-019-02887-4

94. Weiss D, Dunn SI, Sprague AE, Fell DB, Grimshaw JM, Darling E, Graham ID, Harrold J, Smith GN, Peterson WE, Reszel J, Lanes A, Walker MC, Taljaard M. Effect of a population-level performance dashboard intervention on maternal-newborn outcomes: an interrupted time series study. BMJ Qual Saf England; 2018;27(6):425–436. doi: 10.1136/bmjqs-2017-007361

95. Heaton HA, Russi CS, Monroe RJ, Thompson KM, Koch KA. Telehealth dashboard: leverage reporting functionality to increase awareness of high-acuity emergency department patients across an enterprise practice. BMJ Health Care Inform England; 2019;26(1). doi: 10.1136/bmjhci-2019-100093

96. Offodile AC 2nd, Sen AP, Holtsmith S, Escalante J, Park A, Terrell J, Bassett R, Perrier ND. Harnessing Behavioral Economics Principles to Promote Better Surgeon Accountability for Operating Room Cost: A Prospective Study. J Am Coll Surg United States; 2020;230(4):585–593. doi: 10.1016/j.jamcollsurg.2019.12.013

97. Twohig PA, Rivington JR, Gunzler D, Daprano J, Margolius D. Clinician dashboard views and improvement in preventative health outcome measures: a retrospective analysis. BMC Health Serv Res England; 2019;19(1):475. doi: 10.1186/s12913-019-4327-3

98. Rostata-Pesola H, Olender L, Leon N, Aytaman A, Hoffman R, Pesola GR. Hepatitis C virus screening at a Veterans Administration Hospital in New York City. J Am Assoc Nurse Pract United States; 2020;(101600770). doi: 10.1097/JXX.0000000000000393

99. Birdas TJ, Rozycki GF, Dunnington GL, Stevens L, Liali V, Schmidt CM. “Show Me the Data”: A Recipe for Quality Improvement Success in an Academic Surgical Department. J Am Coll Surg United States; 2019;228(4):368–373. doi: 10.1016/j.jamcollsurg.2018.12.013

100. Jaimini U, Thirunarayan K, Kalra M, Venkataraman R, Kadariya D, Sheth A. “How Is My Child’s Asthma?” Digital Phenotype and Actionable Insights for Pediatric Asthma. JMIR Pediatr Parent Canada; 2018;1(2):e11988. doi: 10.2196/11988

101. O’Reilly-Shah VN, Easton GS, Jabaley CS, Lynde GC. Variable effectiveness of stepwise implementation of nudge-type interventions to improve provider compliance with intraoperative low tidal volume ventilation. BMJ Qual Saf England; 2018;27(12):1008–1018. doi: 10.1136/bmjqs-2017-007684

102. Smalley CM, Willner MA, Muir MR, Meldon SW, Borden BL, Delgado FJ, Fertel BS. Electronic medical record-based interventions to encourage opioid prescribing best practices in the emergency department. Am J Emerg Med United States; 2019;(aa2, 8309942). doi: 10.1016/j.ajem.2019.158500

103. Matsumoto S, Koyama H, Nakahara I, Ishii A, Hatano T, Ohta T, Tanaka K, Ando M, Chihara H, Takita W, Tokunaga K, Hashikawa T, Funakoshi Y, Kamata T, Higashi E, Watanabe S, Kondo D, Tsujimoto A, Furuta K, Ishihara T, Hashimoto T, Koge J, Sonoda K, Torii T, Nakagaki H, Yamasaki R, Nagata I, Kira J-I. A Visual Task Management Application for Acute Ischemic Stroke Care. Front Neurol Switzerland; 2019;10(101546899):1118. doi: 10.3389/fneur.2019.01118

104. Anderson BJ, Do D, Chivers C, Choi K, Gitelman Y, Mehta SJ, Panchandam V, Gudowski S, Pierce M, Cereda M, Christie JD, Schweickert WD, Gabrielli A, Huffenberger A, Draugelis M, Fuchs BD. Clinical Impact of an Electronic Dashboard and Alert System for Sedation Minimization and Ventilator Liberation: A Before-After Study. Crit Care Explor United States; 2019;1(10):e0057. doi: 10.1097/CCE.0000000000000057

105. Prabhu A, Agarwal U, Tripathy JP, Singla N, Sagili K, Thekkur P, Sarin R. “99DOTS”techno-supervision for tuberculosis treatment - A boon or a bane? Exploring challenges in its implementation at a tertiary centre in Delhi, India. Indian J Tuberc India; 2020;67(1):46–53. doi: 10.1016/j.ijtb.2019.08.010

106. Ryskina K, Jessica Dine C, Gitelman Y, Leri D, Patel M, Kurtzman G, Lin LY, Epstein AJ. Effect of Social Comparison Feedback on Laboratory Test Ordering for Hospitalized Patients: A Randomized Controlled Trial. J Gen Intern Med United States; 2018;33(10):1639–1645. doi: 10.1007/s11606-018-4482-y

107. Newberry C, Saha A, Siddique SM, Metz DC, Domenico C, Choi K, Gitelman E, Mehta S. A Novel Clinical Decision Support System for Gastrointestinal Bleeding Risk Stratification in the Critically Ill. Jt Comm J Qual Patient Saf Netherlands; 2019;45(6):440–445. doi: 10.1016/j.jcjq.2019.01.001

108. Ebbens MM, Errami H, Moes DJAR, van den Bemt PMLA, van der Boog PJM, Gombert-Handoko KB. Prevalence of medication transfer errors in nephrology patients and potential risk factors. Eur J Intern Med Netherlands; 2019;70(9003220):50–53. doi: 10.1016/j.ejim.2019.09.003

109. Srivastava P, Verma A, Geronimo C, Button TM. Behavior stages of a physician- and coach-supported cloud-based diabetes prevention program for people with prediabetes. SAGE Open Med England; 2019;7(101624744):2050312119841986. doi: 10.1177/2050312119841986

110. Khanna N, Gritzer L, Klyushnenkova E, Montgomery R, Dark M, Shah S, Shaya F. Practice Transformation Analytics Dashboard for Clinician Engagement. Ann Fam Med United States; 2019;17(Suppl 1):S73–S76. doi: 10.1370/afm.2382

111. Jeffries M, Keers RN, Phipps DL, Williams R, Brown B, Avery AJ, Peek N, Ashcroft DM. Developing a learning health system: Insights from a qualitative process evaluation of a pharmacist-led electronic audit and feedback intervention to improve medication safety in primary care. PloS One United States; 2018;13(10):e0205419. doi: 10.1371/journal.pone.0205419
